# Sequenced Care Pathway vs Pain Navigator Pathway for Veterans With Low Back Pain: The AIM-Back Cluster Randomized Clinical Trial

**DOI:** 10.1001/jamanetworkopen.2026.4421

**Published:** 2026-04-02

**Authors:** Steven Z. George, Cynthia J. Coffman, Rebecca North, Trevor A. Lentz, Courtni France, Ashley Choate, Corey B. Simon, Chad E. Cook, Francis J. Keefe, Kelli D. Allen, Adam P. Goode, Heather King, Jennifer Naylor, Lindsay A. Ballengee, Tyler L. Cope, Joseph Leo Brothers, Ivonne Guzman, Travis Linton, Catherine Stanwyck, Christa Tumminello, Susan N. Hastings

**Affiliations:** 1 Duke Clinical Research Institute, Department of Orthopaedic Surgery, Duke University, Durham, North Carolina; 2 Duke Clinical Research Institute, Department of Population Health Sciences, Duke University, Durham, North Carolina; 3 Center of Innovation to Accelerate Discovery and Practice Transformation, Durham Veterans Affairs Health Care System Health Services Research, Durham, North Carolina; 4 Department of Biostatistics and Bioinformatics, Duke University School of Medicine, Durham, North Carolina; 5 Center for the Study of Aging and Human Development, Duke University School of Medicine, Durham, North Carolina; 6 Duke Clinical Research Institute, Center for Aging, Department of Orthopaedic Surgery, Duke University, Durham, North Carolina; 7 Department of Psychiatry and Behavioral Sciences, Duke University, Durham, North Carolina; 8 Division of Rheumatology, Department of Medicine, University of North Carolina at Chapel Hill; 9 Division of General Internal Medicine, Department of Population Health Sciences, Duke University, Durham, North Carolina; 10 Durham Veterans Affairs Health Care System, Mental Illness Research Education Clinical Center, Duke University, Durham, North Carolina; 11 Duke Clinical Research Institute, Durham, North Carolina; 12 Department of Population Health Sciences, Duke University, Durham, North Carolina; 13 Department of Rehabilitation Services, Duke University, Durham, North Carolina; 14 Division of General Internal Medicine, Department of Medicine, Duke University School of Medicine, Durham, North Carolina; 15 Department of Population Health Sciences, Center for the Study of Aging and Human Development, Duke University School of Medicine, Durham, North Carolina

## Abstract

**Importance:**

Low back pain (LBP) is a leading cause of disability, and there is limited evidence from clinical practice to support the effectiveness of alternative care models.

**Objective:**

To compare a sequenced care pathway (SCP) with a pain navigator pathway (PNP) for patients with LBP.

**Design, Setting, and Participants:**

In this embedded cluster randomized clinical trial, 19 primary care clinics in the Veterans Health Administration were randomized to deliver 1 of 2 multimodal guideline-supported care pathways, with primary outcomes assessed in their electronic health records (EHRs) at 3 months. Between February 8, 2021 (first enrolled), and January 31, 2024 (last enrolled), 1817 participants were referred by primary care clinicians and attended an initial AIM-Back trial visit. A subset of 799 participants consented to complete additional questionnaires for secondary analyses (March 8, 2021 [first survey collected], to January 10, 2025 [final secondary outcome collected by survey]).

**Interventions:**

The SCP included pain education and modulation, physical activity coaching, risk stratification, and psychologically informed physical therapy. The PNP included shared decision-making and facilitated referrals to nondrug treatments.

**Main Outcomes and Measures:**

Pain interference and physical function were coprimary outcomes, assessed with the Patient-Reported Outcomes Measurement Information Systems 4-item Short Forms (PROMIS-SF; potential score range for pain interference, 41.6-75.6, where lower scores indicated less interference with daily activities due to pain; and potential score range for physical function, 22.5-57.0, where higher scores indicated higher physical functioning during daily activities). Secondary EHR outcomes included sleep disturbance and National Institutes of Health pain intensity, and survey outcomes included the coprimary outcomes and additional measures of pain, function, and quality of life. Analysis was performed in the intent-to-treat population.

**Results:**

There were 1817 enrolled participants (SCP, 811; PNP, 1006; mean [SD] age, 53.0 [15.7] years; 1597 men [87.9%]). At 3 months, 461 of 811 patients (56.8%) in the SCP group and 537 of 1006 (53.4%) in the PNP group had analyzable primary outcomes. The estimated baseline mean PROMIS-SF score was 63.2 points (97.5% CI, 62.7-63.6 points) for pain interference and 37.1 points (97.5% CI, 36.7-37.4 points) for physical function. The 3-month mean PROMIS-SF score for pain interference was 60.5 points (97.5% CI, 59.7-61.3 points) in the SCP group and 61.1 points (97.5% CI, 60.4-61.8 points) in the PNP group. The 3-month mean PROMIS-SF score for physical function was 39.1 points (97.5% CI, 38.4-39.7 points) in the SCP group and 38.5 points (97.5% CI, 37.8-39.1 points) in the PNP group. There was no SCP superiority, with estimated 3-month differences of −0.6 points (97.5% CI, −1.6 to 0.4 points) for pain interference and 0.6 points (97.5% CI, −0.3 to 1.5 points) for physical function. There were no pathway differences in secondary outcomes.

**Conclusion and Relevance:**

In this cluster randomized trial, the SCP was not superior for the primary outcomes of pain interference and physical function. Future research should consider designs that optimize pathway adherence, assess the effectiveness in other settings, and investigate patient-level factors indicative of a favorable response to the SCP or PNP.

**Trial Registration:**

ClinicalTrials.gov Identifier: NCT04411420

## Key Points

**Question:**

Is a sequenced care pathway superior to a pain navigation care pathway for improving pain interference and physical function outcomes for individuals seeking primary care for low back pain?

**Findings:**

In this cluster randomized clinical trial investigating the effectiveness of different nondrug care pathways in 1817 participants, the 3-month differences in improvements (sequenced care vs navigated care) in pain interference (−0.6 points) and physical function (0.6 points) were not statistically significant.

**Meaning:**

This trial suggests no advantage of a sequenced care pathway vs a care pathway that navigates individuals with low back pain to commonly used nondrug treatments (eg, physical therapy, yoga, tai chi, chiropractic, or acupuncture).

## Teaser

This cluster randomized clinical trial compares a sequenced care pathway with a pain navigator pathway for patients with low back pain for improving pain interference and physical function outcomes.

## Introduction

Globally, the individual and societal burden of chronic low back pain (LBP) is substantial, and reducing this burden is a top health care priority.^1,2^ Specific to the Improving Veteran Access to Integrated Management of Back Pain (AIM-Back) trial, chronic LBP is a leading cause of disability among US veterans. Nondrug treatments for acute and chronic LBP have been endorsed by multiple entities, including the Centers for Disease Control and Prevention,^3^ National Academy of Medicine,^4^ and World Health Organization.^5^ Although these practice guidelines support nondrug treatment of LBP, there remains an urgent need for effectiveness studies.^6^

Collectively, these factors motivated the AIM-Back trial,^7^ conducted as part of the US Department of Veterans Affairs–US Department of Defense–National Institutes of Health (NIH) Pain Management Collaboratory.^8^ AIM-Back differentiated itself from existing clinical trials included in a 2025 Cochrane Review by investigating the effectiveness of 2 alternate care models: a sequenced care pathway (SCP) and a pain navigator pathway (PNP).^9^ Both pathways were multimodal and shared a goal of increasing access to guideline-supported clinical practices. The SCP included in-person and telehealth visits for pain education and modulation, physical activity coaching, risk stratification, and psychologically informed physical therapy, while the PNP, delivered via telehealth, included shared decision-making and facilitated referral to nondrug treatments. Because the SCP involved structured delivery of guideline-supported treatments, we hypothesized that it would have superior 3-month pain interference and physical function outcomes compared with the PNP.

## Methods

### Study Design

The AIM-Back trial was a cluster randomized, practice-embedded (ie, care delivered by clinical staff and data collected during routine visits) clinical trial conducted in 19 clinics in the Veterans Health Administration (VHA) (see trial protocol and statistical analysis plan in Supplement 1). We adhered to the 2025 Consolidated Standards of Reporting Trials (CONSORT) reporting guideline and the CONSORT Extension reporting guideline for cluster randomized trials^10,11^ to report clearly and completely on the design, conduct, analysis, and interpretation of AIM-Back. The trial started enrollment on February 8, 2021, and ended enrollment on January 31, 2024. Clinics ended enrollment when recruitment goals were exceeded or at the end of the trial recruitment period. Additional study details and rationale for care pathways have been published previously.^7,12,13^ The study received approval from the Duke Health institutional review board and the Durham Veterans Affairs Health Care System institutional review board. Participating clinics had agreed to deliver their assigned pathway as standard care; thus, individual-level consent was not required for trial enrollment. AIM-Back had no study-related harms.

### Patient and Public Involvement and Protocol Changes

The care pathways were reviewed and modified based on input from a veteran research engagement panel as well as other partners.^14^ Trials results were reported to this panel for guidance on future LBP care initiatives. AIM-Back updated its data analysis plan prior to completing enrollment.^15^

### Participants

Veterans aged 18 years or older and seeking outpatient care for LBP in a participating primary care clinic were eligible for referral to AIM-Back. Referring clinicians received training on eligibility criteria to ensure consistency. Clinicians were instructed not to refer patients receiving or referred for hospice or palliative care or lacking a telephone number. For the primary analysis intent-to-treat population, referred patients were identified as enrolled participants in the program if they attended the initial AIM-Back visit (baseline) for the clinic-randomized pathway. At the time of AIM-Back referral, veterans were asked if they would be willing to be contacted to complete additional surveys outside the electronic health record (EHR) collection; oral informed consent was provided for these surveys. These data were collected to provide supplemental outcomes and longer-term follow-up beyond 3 months.

### Randomization and Masking

Clinics were recruited by members of the AIM-Back research team (T.A.L., A.C., and T.L.C.) in 2 blocks from February 1 to September 1, 2020, and from March 1 to December 1, 2021. Clinics were eligible to participate in AIM-Back if they agreed to provide clinical personnel to deliver either of the care pathways, did not share clinicians, and had seen between 800 and 5000 unique patients who received a diagnosis of LBP in the previous year.^7^ The lead biostatistician (C.J.C.) used a covariate-constrained randomization^16^ in 2 blocks, with 10 clinics in the first block (randomized September 2020) and 9 clinics in the second block (randomized December 2021). See eAppendix 1 in Supplement 2 for a description of the randomization covariates. Randomized clinics were paired for staggered training and deployment of the randomized pathways by members of the implementation team (T.A.L., C.F., A.C., C.B.S., C.E.C., and T.L.C.). Treating clinicians, study staff members supporting program implementation, and statisticians were not blinded to randomization to allow for cleaning data, pulling variables specific to a given pathway, and developing reports to monitor pathway data quality during the trial. Survey assessors were blinded to care pathway randomization when contacting veterans by telephone for data collection.

### Procedures

During onboarding, clinics were presented with virtual synchronous training modules, a training manual, access to an informational webpage that hosted resources for implementation, and patient flyers for marketing the program. Clinicians were instructed on how to inform their patients about the pathway during their usual clinical care. Throughout the trial, efforts to engage primary care clinicians included sending weekly emails with enrollment goal updates, joining monthly calls to troubleshoot implementation, and performing site visits at facilities that were behind enrollment targets to further engage in-clinic partners.^12,17^

### Care Pathways

The SCP’s core components were pain modulation, physical activity instruction, risk stratification, and psychologically informed physical therapy, if indicated. The SCP provided physical therapy services locally and training in behavioral activation and pain coping skills via telephone (eTable 1 in Supplement 2). The PNP’s core components were shared decision-making, care coordination, and facilitation of referrals to nondrug services. The PNP involved a health care navigator trained by AIM-Back team members in the current recommended treatment guidelines for LBP (eTable 1 in Supplement 2). The PNP involved remote delivery, with most interactions (≥98%) occurring by telephone. Clinicians who served as pain navigators included 10 physical therapists, 4 nurses, 2 chiropractors, and 1 each for occupational therapy, pain medicine, and whole health coach.

### Data Sources

The AIM-Back trial had data entered through 2 sources: (1) the computerized patient record system, the VHA Health Care System EHR in AIM-Back–specific templates by clinicians, and extracted from the Corporate Data Warehouse (CDW), and (2) REDCap^18^ by blinded AIM-Back research staff for the consented subset via telephone survey. Patient-reported outcomes were collected using AIM-Back–specific EHR templates. These outcomes were documented by clinicians at baseline and during subsequent clinical care visits. All outcome data collection occurred during routine appointments, with no additional visits scheduled solely for data collection.

### Primary Outcomes

Patient-Reported Outcomes Measurement Information Systems 4-item Short Forms (PROMIS-SF) scores for pain interference and physical function (potential score range for pain interference, 41.6-75.6, where lower scores indicated less interference with daily activities due to pain; and potential score range for physical function, 22.5-57.0, where higher scores indicated higher physical functioning during daily activities) were collected by clinicians at baseline and 3-month follow-up. For veterans who did not have 3-month PROMIS-SF outcomes scores in the EHR, the PROMIS-SF data from surveys were used if available within the appropriate time window^15^ (eFigure 1 in Supplement 2).

### Secondary Outcomes

PROMIS-SF sleep disturbance scores and NIH pain intensity were collected in the EHR as secondary outcomes. The 12-month opioid outcomes extracted from pharmacy refill data in CDW will be presented in a separate article focusing on health care utilization. For those consenting to surveys, PROMIS-SF outcomes (pain interference, physical function, and sleep disturbance), pain intensity (PEG [pain intensity, interference with enjoyment of life, and interference with general activity]), pain catastrophizing (2 items from the NIH Task Force), self-efficacy (PSEQ-2 [Pain Self-Efficacy Questionnaire–2]), quality of life (EQ-5D-5L [EuroQoL 5-dimension 5-level]), depressed mood (PHQ-2 [Patient Health Questionnaire–2]), and alcohol use (AUDIT-C [Alcohol Use Disorders Identification Test–Consumption screener for problem drinking]) were collected at baseline and 3-, 6-, and 12-month follow-up. See eTable 2 in Supplement 2 for additional details.

### Covariates

Demographic characteristics (age, sex, and race and ethnicity [Black or African American, Hispanic, White, multiracial or other race or ethnicity (American Indian or Alaska Native, Asian, Native Hawaiian or Pacific Islander)]) and Care Assessment Need (CAN) scores prior to referral date (closest) were extracted from the CDW. The CAN score,^19^ a comorbidity measure, is a risk percentile based on the estimated risk for hospital admission or death within 1 year calculated weekly for all eligible veterans (range, 0 [lowest risk] to 99 [highest risk]). Chronic pain status was operationally defined by 2 items from the Graded Chronic Pain Scale Revised questionnaire^20^ collected in the EHR. Race and ethnicity data were collected for the following reasons: (1) we were interested in assessing the representativeness by age, sex, and race and ethnicity for those enrolled in the trial versus those seeking care at the clinic (these data are part of a different publication investigating representativeness of those enrolled in the AIM-Back trial); (2) randomization occurred at the group level, so this information was needed to assess cluster balance after randomization, especially given the geographic distribution of participating clinics; and (3) these variables were included a priori in our primary analysis as proxy measures of sociodemographic status.

### Statistical Analysis

Oversight was provided by a data safety monitoring board convened by the NIH. Additional details on our analysis are provided in eAppendix 1 in Supplement 1.

With 16 clinics (8 PNP, 8 SCP), 105 patients per clinic with baseline pathway visits (1680 patients), and a type I error of 2.5% (to account for coprimary outcomes), we had 90% power to detect medium to large standardized Cohen *d* effect size differences from 0.30 to 0.50 in the primary outcomes across the range of assumed intraclass correlations coefficients (ICCs) from 0.01 to 0.05 (to account for clustering of outcome within clinics).^19^ Assuming SDs of 8 and 10, respectively, these medium to large effect size differences translate to differences of 2.4 to 5.0 points for either PROMIS-SF pain interference or physical function between arms. Sample size calculations were based on the net difference between arms across baseline and 3-month follow-up,^21^ with 0.50 correlation assumed over time, 20% attrition rate, and adjustment for the randomization covariates. With 53 patients per clinic participating in the survey study (n = 848; approximately 50% of those participating in AIM-Back pathway), we had 90% power to detect effect size differences from 0.33 to 0.55 for both primary outcomes.

Analyses were conducted on an intent-to-treat basis; participants were assigned to the randomized pathway of the clinic to which the referring clinician was assigned. Analyses were conducted separately for EHR-measured and survey-measured outcomes; to facilitate pathway comparison, survey outcomes were analyzed only from the survey participants who were also enrolled in the trial (n = 799). All analyses were conducted with SAS software, version 9.4 (SAS Institute Inc) or R software, version 4.4.0 (R Project for Statistical Computing).

Hierarchical linear mixed-effects models were used for all outcomes, with patients nested within clinics and baseline and follow-up outcomes in the response vector.^22^ The fixed effects in the model included follow-up time indicators and treatment-by-time interaction indicators, as well as the clinic-level randomization covariates and prespecified patient-level covariates (age, sex, race and ethnicity, chronic pain status, and CAN score). This model assumes that study arms have equal baseline mean values. Mean or mode imputation by clinic was conducted for missing CAN score and race and ethnicity, as appropriate.^23,24^ Random effects for clinics and clinics by time were included to account for clustering of patients within the clinic and an unstructured covariance for the patient-level covariance structure. Estimated treatment mean values and differences at follow-up times with associated 97.5% CIs for coprimary outcomes and 95% CIs for secondary outcomes and *P* values are presented. Sensitivity analyses were conducted among enrolled participants (n = 1817) to investigate (1) treatment difference using all time points (both EHR and survey; range, 1-8 per patient), (2) missing data, and (3) referral bias. Statistical tests were 2-sided. For coprimary outcomes, *P* < .025 was considered statistically significant; for secondary outcomes, *P* < .05 considered statistically significant

## Results

**Figure 1.**
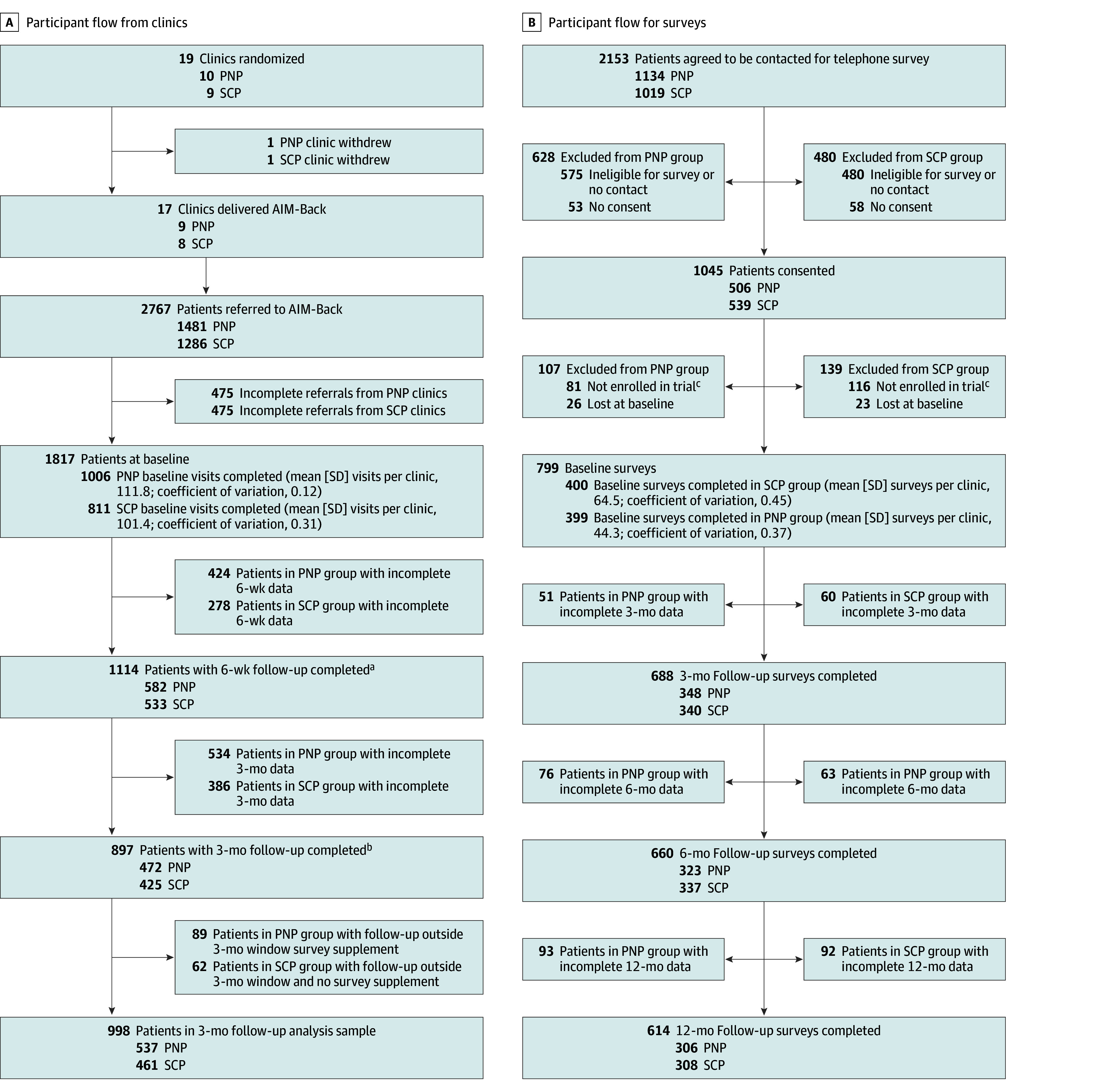
Participant Flow Diagrams A, Participant flow from clinics in Improving Veteran Access to Integrated Management of Back Pain (AIM-Back) trial. B, Participant flow for survey participants in AIM-Back trial. ^a^There were 35 veterans in the pain navigator pathway (PNP) arm with multiple 6-week follow-up visits (32 with 2 visits, 3 with 3 visits) and 18 veterans in the sequenced care pathway (SCP) arm with multiple 6-week follow-up visits (17 with 2 visits, 1 with 3 visits). ^b^There were 8 veterans with two 3-month follow-up visits in the PNP arm and 1 veteran with two 3-month follow-up visits in the SCP arm. ^c^These veterans completed surveys for the trial but did not receive the clinical intervention.

The study enrolled 1817 participants (mean [SD] age, 53.0 [15.7] years; 1597 men [87.9%] and 220 women [12.1%]; 541 Black or African American [29.8%], 95% Hispanic [5.2%], 1208 White [66.5%], and 68 other race or ethnicity or multiracial [3.7%]) (Table 1). Nineteen clinics agreed to participate in AIM-Back and were randomized (10 PNP; 9 SCP) between February 2020 and December 2021. Two clinics, 1 in each arm, withdrew prior to launching AIM-Back and were excluded from all analyses (Figure 1A). A mean (SD) of 13.7 (8.2) clinicians per clinic were identified during recruitment. Patients with LBP across clinics over the 6-month period prior to clinic enrollment had a mean (SD) age of 61.3 (2.0) years, a mean (SD) pain intensity of 4.2 (0.7) (range, 0-10, where higher scores indicate greater pain), and mean (SD) rates of any opioid use of 17.7% (5.9%) (Table 1).

**Table 1.** Characteristics of Clinics and Enrolled Participants at Baseline

Characteristic	Overall	PNP	SCP
**Clinic characteristics**			
Clinics, No.	17	9	8
Clinic type, No. (%)			
Main medical center	8 (47.1)	5 (55.6)	3 (37.5)
Community	9 (52.9)	4 (44.4)	5 (62.5)
No. of clinicians, mean (SD)	13.7 (8.2)	13.6 (7.1)	13.9 (9.7)
Age of patients with LBP, mean (SD)^a^	61.3 (2.0)	61.4 (2.0)	61.1 (2.1)
Pain intensity (range, 0-10), mean (SD)^a^	4.2 (0.7)	4.2 (0.8)	4.2 (0.5)
Opioid use, mean (SD), %^a^^,^^b^	17.7 (5.9)	18.2 (7.6)	17.1 (3.3)
**Patient characteristics** ^c^			
Patients, No.	1817	1006	811
Age, mean (SD), y	53.0 (15.7)	52.7 (15.9)	53.4 (15.4)
Age categories, No. (%)			
<50 y	766 (42.2)	435 (43.2)	331 (40.8)
50-64 y	562 (30.9)	296 (29.4)	266 (32.8)
65-74 y	330 (18.2)	192 (19.1)	138 (17.0)
≥75 y	159 (8.8)	83 (8.3)	76 (9.4)
Sex, No. (%)			
Female	220 (12.1)	110 (10.9)	110 (13.6)
Male	1597 (87.9)	896 (89.1)	701 (86.4)
Race, No. (%)^d^			
Black or African American	541 (29.8)	233 (23.2)	308 (38.0)
Other or multiracial^e^	68 (3.7)	44 (4.4)	24 (3.0)
White	1208 (66.5)	729 (72.5)	479 (59.1)
Hispanic ethnicity, No. (%)^d^	95 (5.2)	59 (5.9)	36 (4.4)
High-impact chronic pain, No. (%)	1194 (65.7)	660 (65.6)	534 (65.8)
Chronic LBP, No. (%)^f^	1672 (92.0)	921 (91.6)	751 (92.6)
Opioid use, No. (%)	179 (9.9)	109 (10.8)	70 (8.6)
Chronic opioid use, No. (%)	64 (3.5)	36 (3.6)	28 (3.5)
Benzodiazepine use, No. (%)	89 (4.9)	48 (4.8)	41 (5.1)
Chronic benzodiazepine use, No. (%)	38 (2.1)	16 (1.6)	22 (2.7)
1-y CAN Score, mean (SD)	46.1 (30.1)	47.3 (30.7)	44.6 (29.3)
PTSD, No. (%)	425 (23.4)	215 (21.4)	210 (25.9)
Area Deprivation Index, mean (SD)	57.7 (22.9)	58.4 (23.4)	56.8 (22.3)

Abbreviations: CAN, Care Assessment Need; LBP, low back pain; PNP, pain navigator pathway; PTSD, posttraumatic stress disorder; SCP, sequenced care pathway.

^a^ Assessed patients with LBP were seen at participating clinics in 6 months prior to clinic enrollment.

^b^ Clinic-level percentage at time of enrollment.

^c^ Missing characteristics imputed by site: 1-year CAN Score (mean), 51 missing (41 PNP, 10 SCP); race and ethnicity (mode), 36 missing (27 PNP, 9 SCP).

^d^ Information on race and ethnicity was collected from the electronic health record, which was entered by clinical staff during a routine visit.

^e^ Other races included American Indian or Alaska Native, Asian, Native Hawaiian or Pacific Islander.

^f^ Chronicity as defined by the National Institutes of Health.

Between February 1, 2021, and January 18, 2024, there were 2767 veterans referred (1481 PNP; 1286 SCP) to the AIM-Back pathways (Figure 1A). Of those referred, 1817 (65.7%) enrolled, with 1006 of 1481 (67.9%) in the PNP group and 811 of 1286 (63.1%) in the SCP group. Of the 2767 referrals, 2153 (77.8%) agreed to be contacted for surveys, and 1045 veterans consented, with 996 completing baseline surveys, 799 (399 PNP; 400 SCP) of whom were enrolled in the trial (Figure 1B). Baseline characteristics of enrolled survey participants^25^ are summarized in eTable 3 in Supplement 2.

Adherence metrics for the SCP group are reported in Table 2,^26^ and adherence metrics for the PNP group are reported in Table 3. Overall, 582 of 1006 veterans (57.9%) in the PNP group and 532 of 811 (65.6%) in the SCP group completed the 6-week follow-up session, and 472 (46.9%) in the PNP group and 425 (52.4%) in the SCP group completed the 3-month follow-up visit. There were 252 veterans (154 PNP; 98 SCP) without 3-month EHR outcome data who had survey data collected in the appropriate window, which increased the yield to 537 of 1006 veterans (53.4%) in the PNP group and 461 of 811 veterans (56.8%) in the SCP group with analyzable primary outcomes at 3 months (Figure 1A; eFigure 1 in Supplement 2). There were no differences in baseline characteristics between those providing 3-month EHR outcome and those that did not (eTable 4 in Supplement 2). Among the 799 enrolled participants consenting to telephone surveys, follow-up rates were 87.2% (348 of 399) in the PNP group and 85.0% (340 of 400) in the SCP group at 3 months, 81.0% (323 of 399) in the PNP group and 84.3% (337 of 400) in the SCP group at 6 months, and 76.7% (306 of 399) in the PNP group and 77.0% (308 of 400 in the SCP group) at 12 months (Figure 1B).

**Table 2.** Adherence Metrics for Delivery of Sequenced Care Pathway^**a**^

Session and adherence metric	Value
**Initial visit**	
Visit attended, No. (%)	811 (100)
≥1 Pain modulation (massage, manual therapy, TENS) and/or pain neuroscience education sessions completed, No./total No. (%)	745/811 (91.9)
**Physical activity coaching**	
Visits (remote delivery), mean (SD), No.	2.4 (1.9)
Participants with ≥1 visits, No./total No. (%)	625/811 (77.1)
**6-wk Follow-up^b^**	
Visits attended, No./total No. (%)	532/811 (65.6)
≥1 Pain modulation and/or pain neuroscience education sessions completed, No./total No. (%)	336/532 (63.2)
STarT Back screening^c^ as medium or high risk, No./total No. (%)	377/532 (70.9)
**Psychologically informed practice^d^**	
Visit (remote delivery), mean (SD), No.	1.9 (2.0)
Participants with ≥1 visits, No./total No. (%)	218/377 (57.8)
**3-mo Follow-up^e^**	
Visits attended, No./total No. (%)	425/811 (52.4)

Abbreviations: STarT Back, Subgroups of Targeted Treatment for Back Pain; TENS, transcutaneous electrical nerve stimulation.

^a^ Mean (SD) number of physical therapy sessions for those with at least 1 session: 3.1 (1.5); mean (SD) number of psychologically informed practice sessions for those with at least 1 PiP: 3.3 (1.6).

^b^ There were 18 patients with multiple 6-week follow-up visits in the sequenced care pathway arm (17 with 2 visits, 1 with 3 visits).

^c^ Evaluated using the STarT Back tool.^26^

^d^ Psychologically informed practice sessions for 377 veterans stratified to high or medium risk; there were an additional 7 veterans with missing 6-week follow-up (n = 6) or stratified to low risk (n = 1) that received at least 1 psychologically informed practice call.

^e^ There was 1 veteran with two 3-month follow-up visits.

**Table 3.** Adherence Metrics for Delivery of Pain Navigator Pathway

Session and adherence metric	No. with metric/total No. eligible (%)
**Initial (baseline)**	
Visits, Total No.	1006 (100)
≥1 Referrals issued to a service	955/1006 (94.9)
Multiple referrals to a service	423/955 (44.3)
Referral type^a^	
PT, chiropractic, or acupuncture^b^	842/955 (84.5)
PT	527/955 (55.1)
Chiropractic	356/955 (37.3)
Acupuncture	284/955 (29.7)
Yoga or tai chi	137/955 (14.3)
Multidisciplinary pain clinic	62/955 (6.5)
Other (eg, pain school, massage therapy, whole health)	161/955 (16.8)
**6-wk Follow-up^c^**	
Visits	582/1006 (57.9)
≥1 Referrals issued to a service	413/582 (70.8)
Multiple referrals to a service	142/413 (34.3)
Referral type^a^	
PT, chiropractic, or acupuncture^b^	341/413 (82.5)
PT	210/413 (50.8)
Chiropractic	126/413 (30.5)
Acupuncture	108/413 (26.2)
Yoga or tai chi	44/413 (10.7)
Multidisciplinary pain clinic	13/413 (3.1)
Other (eg, pain school, massage therapy, whole health)	78/413 (18.9)
**3-mo Follow-up^d^**	
Visits	472/1006 (46.9)
Self-reported receipt of pain navigator recommended services (VA or non-VA)	381/472 (80.7)
Multiple services received	192/381 (50.4)
Service type	
PT, chiropractic, or acupuncture^b^	347/381 (91.2)
PT	229/381 (60.1)
Chiropractic	158/381 (41.5)
Acupuncture	86/381 (22.7)
Yoga or tai chi	33/381 (8.9)
Multidisciplinary pain clinic referral	27/381 (7.1)
Other	63/381 (16.5)

Abbreviations: PT, physical therapy; VA, Department of Veterans Affairs.

^a^ Less than 5% of patients with referrals to cognitive behavioral therapy, counseling, or biofeedback; recreation therapy; occupational therapy; exercise program; or MOVE! Weight Management Program.

^b^ Top 3 services referred by pain navigators across clinics.

^c^ There were 35 veterans with multiple 6-week follow-up visits in pain navigator pathway arm (32 with 2 visits, 3 with 3 visits).

^d^ There were 8 veterans with two 3-month follow-up visits.

### Primary Outcomes

**Figure 2.**
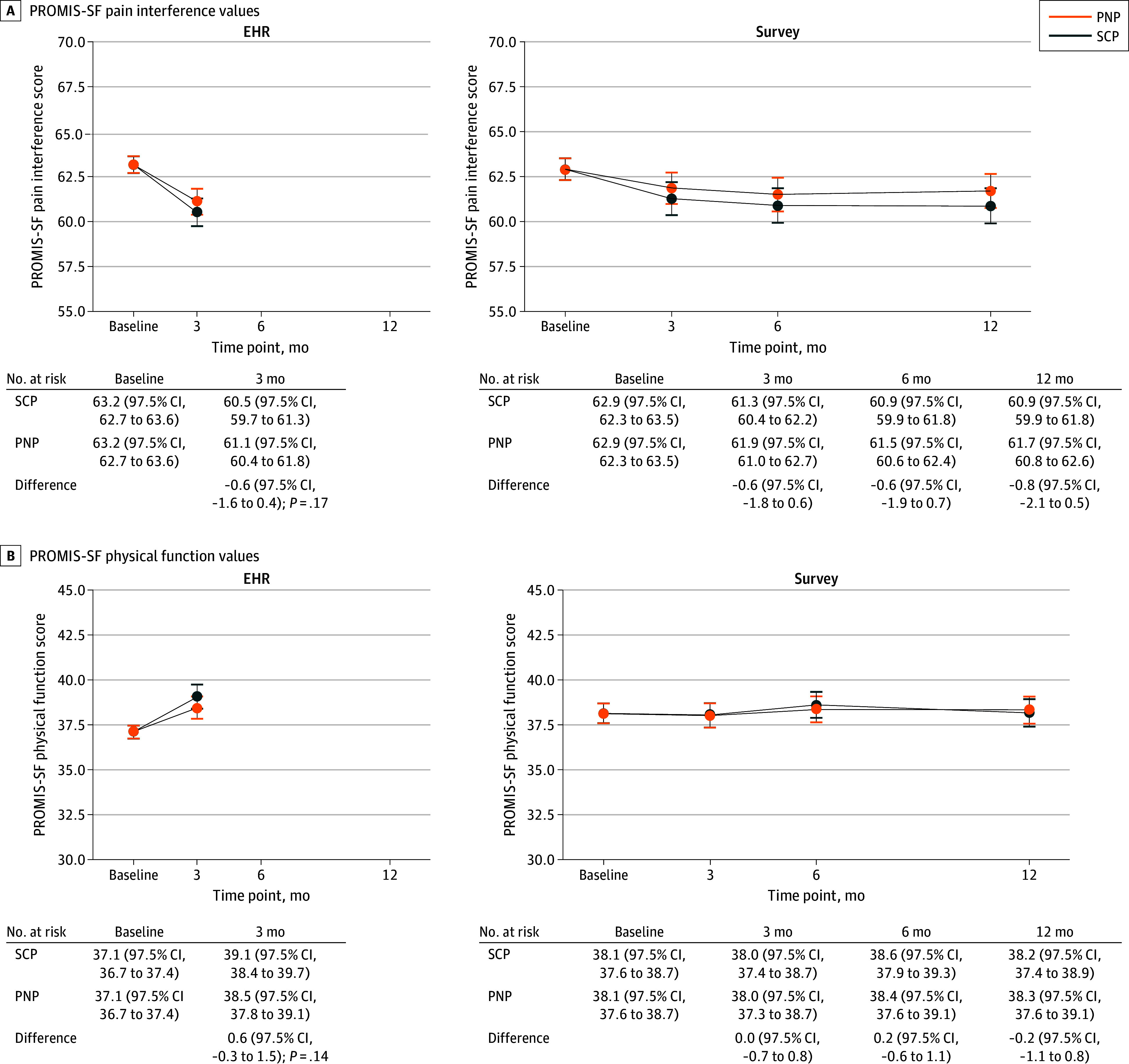
Line Graphs of Pain Interference and Physical Function Patient-Reported Outcomes Measurement Information Systems 4-item Short Forms (PROMIS-SF) Outcomes Estimated mean values and mean difference at follow-up time points for PROMIS-SF pain interference (A) and PROMIS-SF physical function (B) outcomes for electronic health record (EHR) data from enrolled participants (primary; n = 1817) and survey participants (n = 799) from hierarchical linear mixed-effects models. The observed range of values for PROMIS-SF pain interference scores was from 41.6 to 75.6; the observed range of values for PROMIS-SF physical function scores was from 22.9 to 56.9. Error bars indicate 97.5% CIs.

There were no differences between pathways in the primary PROMIS-SF outcomes at 3 months (n = 1817); the estimated differences were −0.6 points (97.5% CI, −1.6 to 0.4 points; *P* = .17) for the SCP vs PNP for pain interference and 0.6 points (97.5% CI, −0.3 to 1.5 points; *P* = .14) for physical function (Figure 2). The estimated baseline mean pain interference PROMIS-SF score was 63.2 points (97.5% CI, 62.7-63.6 points) in the SCP and PNP groups, with 3-month mean values of 60.5 points (97.5% CI, 59.7-61.3 points) in the SCP group and 61.1 points (97.5% CI, 60.4-61.8 points) in the PNP group. The estimated baseline mean for physical function was 37.1 points (97.5% CI, 36.7-37.4 points) in the SCP and PNP groups, with 3-month mean values of 39.1 points (97.5% CI, 38.4-39.7 points) in the SCP group and 38.5 points (97.5% CI, 37.8-39.1 points) in the PNP group. The ICC was 0.0 for pain interference (could not estimate random effect for clinic and time) and 0.008 for physical function. The within-group difference was −2.6 points (97.5% CI, −3.3 to −1.9 points) for pain interference and 2.0 points (97.5% CI, 1.3-2.7 points) for physical function for the SCP group, and it was for pain interference was −2.1 points (97.5% CI, −2.7 to −1.4 points) for pain interference and 1.4 points (97.5% CI, 0.7-2.0 points) for physical function for the PNP group. These differences translate to effect size differences in the moderate range (0.4-0.5) for SCP and small to moderate range (0.3-0.4) for PNP. Results of sensitivity analyses using all time points, multiple imputation, and adjusting for referral bias were similar to the primary analysis (eFigures 2a, 2b, 3, and 4 in Supplement 2).

### Secondary Outcomes

There were no differences between pathways in EHR-collected secondary outcomes at 3 months; the estimated differences were −0.6 points (95% CI, −1.8 to 0.6 points) for sleep disturbance and −0.3 (95% CI, −0.7 to 0.04) for NIH pain intensity (eFigure 5 in Supplement 2). Among survey participants (n = 799), there were no differences between pathways in PROMIS-SF pain interference or physical function (Figure 2) or in any other secondary survey outcomes at follow-up time points (eTable 5 in Supplement 2).

## Discussion

We hypothesized greater effects of the SCP because of its structured delivery of guideline-supported treatments.^7^ However, the SCP was not superior to the PNP for the 3-month coprimary outcomes, nor at any additional follow-up times. Participants in both pathways experienced improvements in the lower range of clinical importance for the primary outcome measures.^27,28^ The 2025 Cochrane review of LBP care models including 48 trials (only 14 from the US) found small but not clinically relevant differences favoring alternative care models (eg, management of care processes or use of information technology).^6^ AIM-Back is one of the first trials to our knowledge to eschew usual care as a comparator and, as such, provides foundational data on comparative effectiveness. AIM-Back investigated 2 care models in an “A vs B” trial (ie, 2 different treatment approaches), a design that is lacking in the literature but is vital for informing health systems on different care delivery options.^29^ The SCP has the advantage of limiting variability in care, while its primary disadvantage is that it disrupts existing clinical workflows. The PNP has the advantage of offering a patient-centered approach through its flexibility in care options but the disadvantage of needing numerous nondrug options to refer for services. AIM-Back findings indicate that navigator options in the PNP had comparable outcomes with those of the structured approach in the SCP and may be viable for testing effectiveness for other common pain conditions.

### Strengths and Limitations

The AIM-Back trial has some strengths, such as covariate-constrained randomization and pragmatic elements, including broad enrollment criteria, recruitment from a diverse group of clinical sites, high uptake of the clinical pathways, use of existing clinical staff to deliver the care pathways, and inclusion of survey participants to gather additional data. The trial also has some limitations. A primary limitation was low follow-up rates for the primary outcomes. This was likely a reflection of capturing data during routine care, as participants consenting to survey completion followed up at much higher rates (≥85% at 3 months). Despite the lower follow-up rate, we have confidence in the credibility of AIM-Back findings for several reasons. First, participants with or without EHR follow-up data were similar. Second, our power calculations assumed a loss to follow-up rate of 20% and enrolling 1680 participants across 16 clinics; we enrolled 1817 across 17 clinics with a loss to follow-up rate of 45%. In our power calculation, we assumed larger ICCs and SDs than observed, which may balance out the higher loss to follow-up. Furthermore, the estimated effect size differences for both outcomes were below 0.14, with the 97.5% CI bound translating to an effect size of 0.34 on the lower 0.30 to 0.50 range that we were powered to detect, indicating that inadequate power was not an issue. Third, sensitivity analyses using all available measures (EHR and survey) and multiple imputation yielded similar results to the primary analysis. Fourth, the analysis of survey outcomes only (with the higher follow-up rates) yielded similar results to the primary analysis.

As noted, this trial did not include a usual care arm, which can be considered another limitation. This choice was consistent with our goal of offering participating sites at least 1 option for implementing a new care pathway. Given there was no usual care comparator, some within-arm improvements may reflect regression to the mean.

## Conclusions

The AIM-Back cluster randomized clinical trial adds to the existing literature by being the first to our knowledge to compare 2 alternative LBP care models and finding no superiority of the SCP over the PNP for pain interference and physical function outcomes. Future research should consider designs that optimize pathway adherence, assess effectiveness in other settings, and investigate patient-level factors indicative of a favorable response to the SCP or PNP.

## Notes

Supplement 1.
Trial Protocol and Statistical Analysis Plan

Supplement 2. **eAppendix 1.** Statistical Analysis Methods **eAppendix 2.** Secondary and Sensitivity Results **eAppendix 3.** Adherence Results **eAppendix 4.** Adverse Events for Survey Participants **eAppendix 5.** SAS Code for Fitting Hierarchical Linear Models in PROC MIXED **eFigure 1.** AIM-Back CONSORT **eTable 1.** Pathway Descriptions **eTable 2.** Patient-Reported Outcome Measures and Measurement Properties (CONSORT-PRO) **eTable 3.** Patient Characteristics From Survey at Baseline **eTable 4.** Baseline Characteristics of EHR Patients by 3M Outcome Missingness **eFigure 2a.** Enrolled Participants (n=1817) With All Time Points (EHR and Survey) for PROMIS-SF Pain Interference **eFigure 2b.** Enrolled Participants (n=1817) With All Time Points (EHR and Survey) for PROMIS-SF Physical Function **eFigure 3.** Estimated Means (Blue Text SCP; Red Text PNP) and Mean Difference (Black Text) at Follow-Up Time Points for PROMIS Pain Interference and Physical Functions Outcomes and Associated 97.5% Confidence Intervals for Enrolled Participants (n=1817) From Multiply Imputed Data With Combined Estimates Across n=50 Imputation Fit to Hierarchical Linear Mixed Models **eFigure 4.** Estimated Means (Blue Text SCP; Red Text PNP) and Mean Difference (Black Text) at Follow-Up Time Points for PROMIS Pain Interference and Physical Functions Outcomes and Associated 97.5% Confidence Intervals for Enrolled Participants (n=1817) From IPW Models Adjusted for Referral Bias Fit to Hierarchical Linear Mixed Models **eFigure 5.** Estimated Means (Blue Text SCP; Red Text PNP) and Mean Difference (Black Text) at Follow-Up Time Points for PROMIS Sleep Disturbance and NIH Pain Intensity Outcomes and Associated 95% Confidence Intervals for Enrolled Participants (Primary; n=1817) and Survey Participants (n=799) From Hierarchical Linear Mixed-Effects Models **eTable 5.** Estimated Means and Mean Differences at Follow-Up Time Points for Secondary Outcomes and Associated 95% Confidence Intervals for Survey Participants (n=799) From Hierarchical Linear Mixed-Effects Models
eReferences.

Supplement 3.
Data Sharing Statement
