# Supplementary material for: Sequenced Care Pathway vs Pain Navigator Pathway for Veterans With Low Back Pain: The AIM-Back Cluster Randomized Clinical Trial
Source: JAMA Netw Open. 2026 Apr 2;9(4):e264421. doi: 10.1001/jamanetworkopen.2026.4421 (PMC13047465; doi:10.1001/jamanetworkopen.2026.4421)
Supplement: Supplement 1. — Trial Protocol and Statistical Analysis Plan [file jamanetwopen-e264421-s001.pdf]

Improving Veteran Access to Integrated Management of Back Pain (AIM-Back): An Embedded Pragmatic, Cluster Randomized Trial

AIM-Back

Principal Investigators:

Steven Z. George, PT, PhD
S. Nicole Hastings, MD, MHS

Supported by:

The National Center for Complementary and Integrative Health (UH3AT009790)

Study Procedures Provided by:

Durham VA Health Care System
Duke University

Protocol Version 0.51

Tool Revision History

Version Number: 0.1
Version Date: January 23rd, 2019
Summary of Revisions Made: Original source document was the grant proposal from March 2017. Protocol revisions were made in preparation for Duke University and Durham VA IRB submission, as well as for NCCIH protocol review.

Revisions include: 1) updates to the care pathways from stakeholder engagement process; 2) modifications to sample size and analysis plan following Biostatistics Working Group review; 3) updated language on data sharing agreement; 4) detail on process for site selection; 5) description of process for capturing longitudinal data via the outcomes call center

Version Number: 0.2
Version Date: March 4th, 2019
Summary of Revisions Made: This version of the protocol included updates from the initial NCCIH review including 1) updates on the selection process for participating sites; 2) additional details describing the care pathways (including updated care pathway figures); 3) better definitions for pathway adherence; 4) appendices for SPIRIT and TIDier documents; 5) answered queries from NIH review; and 6) appendix that includes the outline for pathway training materials (Milestone for completed materials is June 2019).

Version Number: 0.3
Version Date: May 3rd, 2019
Summary of Revisions Made: This version of the protocol continues the responses and updates from NCCIH review including 1) Update on the sites that will participate in the clinical pathways; 2) More detailed information on how improvement will be determined in the Pain Navigator Pathway; 3) Protocol clarifications on inclusion criteria (for operational definition of LBP and geographic distance); 4) Methodological clarification on how telephone subset will be selected; 5) Statistical analysis clarification regarding statistician role and clinic drop out; 6) Addition of DSMP Template as Appendix to address Safety Monitoring portion of the protocol.

Version Number: 0.4
Version Date: June 28, 2019
Summary of Revisions Made: This version of the protocol contains responses to 1) Providing more details and plans for recruiting site recruitment and 2) updates on IRB approval for Duke University and Durham VA. There is supporting documentation for these protocol updates sent along with this revised protocol.

Version Number: 0.41
Version Date: August 15, 2019
Summary of Revisions Made: This version of the protocol includes: 1) Removed comments and accepted track changes for issues that appeared to be resolved; 2) IRB approval for Duke University and Durham VA approval that includes waiver of consent; 3) Fully executed data use agreement between Duke and Durham VA; 4) Updates to randomization schedule to account

for potential clinic dropout; 5) Site recruitment letter from VISN6 director indicating that site participation agreements can be pursued once protocol is finalized; 6) Updated data collection templates for creation of health factors in VA electronic health record; 7) Manuals for training sites in implementing the clinical pathways; 8) Added study logo to title page.

Version Number: 0.50
Version Date: October 25, 2019
Summary of Revisions Made: This version of the protocol includes responses to the Protocol Review Committee comments from September 24 review call: 1) Clarified procedures and analysis plan for timing of 3-month primary endpoint ascertainment; 2) Provided additional information on the qualitative approach; 3) Provided additional information on implementation approach including theoretical framework and evaluation plans; 4) Updated contact of eligible subjects from 4 to up to 10 call attempts will be made; 5) Nomenclature updated for the PNP, removing all remnants of the CMP acronym.

Version Number: 0.51
Version Date: January 20, 2024
Summary of Revisions Made: This version of the protocol: 1) Updated the analysis plan due to higher rates of loss to follow-up in the EHR sample at 3-months as approved by NCCIH; 2) Updated the adherence measure definition; 3) Provided additional clarification of EHR outcomes; 4) Removed follow-up survey time point of 9 months; 5) Updated study personnel; 6) Included link to pre-print server that is hosting the updated statistical analysis plan; 7) Added the clinical trial registry number to this document; and 8) Streamlined document by removing Appendices/Supplemental Files as most were no longer relevant or out of date.

Additional updates made by the study team; 1) we now refer to AIM-Back as an “embedded pragmatic trial”, as this is a better descriptor of the study; 2) age specified as a patient level characteristic that will be considered for covariate constrained randomization; 3) Changed the name of the Integrated Care Pathway (ICP) to Sequence Care Pathway (SCP) and Coordinated Care Pathway (CCP) to Pain Navigator Pathway (PNP) as these names were used in the protocol paper and at clinics for delivering programs There were no changes to the content of the care pathways with these name changes and they could be used interchangeably. However, in order to avoid confusion and to be consistent with the terminology used for implementation we made these changes in the protocol

TABLE OF CONTENTS

Page

Tool Revision History 2

TABLE OF CONTENTS 4

STUDY TEAM ROSTER 7

PARTICIPATING CLINICS 9

PRÉCIS 10

1. STUDY OBJECTIVES 12

1.1 Primary Objective 12

1.2 Secondary Objectives 13

2. BACKGROUND AND RATIONALE 13

2.1 Background on Condition, Disease, or Other Primary Study Focus 13

2.2 Study Rationale 13

2.3 Sequenced Care Pathway 15

2.4 Pain Navigator Pathway 19

3. STUDY DESIGN 21

4. SELECTION AND ENROLLMENT OF CLINICAL SITES AND PARTICIPANTS ... 23

4.1 Participant Inclusion Criteria 23

4.2 Participant Exclusion Criteria 24

4.3 Study Enrollment Procedures for Outcome Collection 24

5. IMPLEMENTATION OF CARE PATHWAYS 26

5.1 Qualitative Data Collection 26

5.2 Concomitant Interventions 27

5.2 Adherence Assessment 27

6. STUDY PROCEDURES 30

6.1.1 Sequenced Care Pathway 31

Central Delivery Physical Activity Phone Call Template 31

Central Delivery 3-month follow-up Phone Call Data Collection Template 32

6.1.2 Pain Navigator Pathway 32

6.2 VA Corporate Data Warehouse Collection 33

6.3 Blinding 33

7. SAFETY ASSESSMENTS 35

7.1 Adverse Events and Serious Adverse Events 35

7.2 Safety Monitoring 35

8. CLINIC DISCONTINUATION 36

9. STATISTICAL CONSIDERATIONS 37

9.1 General Design Issues 37

9.2 Sample Size and Randomization 37

9.2.1 Treatment Assignment Procedures 38

9.3 Definition of Populations 39

9.4 Interim Analyses and Stopping Rules 40

9.5 Outcomes 40

9.5.1 Primary Outcome 40

9.5.2 Secondary Outcomes 40

9.6 Data Analyses 40

10. MEASURES AND DATA COLLECTION 45

10.1 Survey Data Collection Process 48

10.2 Data Management 48

10.3 Quality Assurance 48

10.3.2 Quality Control Committee 49

10.3.3 Metrics 49

10.3.4 Protocol Deviations 49

10.3.5 Monitoring 49

11. PARTICIPANT RIGHTS AND CONFIDENTIALITY 49

11.1 Institutional Review Board (IRB) Review 50

11.2 Informed Consent Forms 51

11.3 Participant Confidentiality 51

11.4 Study Discontinuation 52

12. COMMITTEES 52

13. PUBLICATION OF RESEARCH FINDINGS 52

14. REFERENCES 54

157 **STUDY TEAM ROSTER**

Name	Address	Telephone	Fax	E-mail
Steven Z. George, PT, PhD (PI)	DCRI, 200 Morris St, Durham, NC 27701	(919) 668-0825	(919) 684-1846	steven.george@duke.edu
S. Nicole Hastings, MD, MHS (PI)	Durham VAHCS, HSRD (152) 508 Fulton St. Durham, NC 27705	(919) 286-6936	(919) 416-5836	susan.hastings@duke.edu
Kelli Allen, PhD (Co-Investigator)	Box 7280, UNC, Chapel Hill, NC 27599	(919) 966-0558	(919) 966-1739	kdallen@email.unc.edu
Chad Cook, PT, PhD, MBA (Co-Investigator)	DUMC 104002, 2200 W Main St. Ste B230, Durham, NC 27710	(919) 684-8905	(919) 684-1846	chad.cook@duke.edu
Corey Simon, DPT, PhD (Co-Investigator)	DUMC 104002, 2200 W Main St. Ste B230, Durham, NC 27710	(919) 681-1692	(919) 684-1846	corey.simon@duke.edu
Adam Goode, DPT, PhD (Co-Investigator)	DCRI, 200 Morris St, Durham, NC 27701	(919) 681-6154	(919) 684-1846	adam.goode@duke.edu
Heather King, PhD (Co-Investigator)	Durham VAHCS, HSRD (152) 508 Fulton St. Durham, NC 27705	(919) 286-6936	(919) 416-8025	heather.king@duke.edu
Catherine	Durham	(919) 286-	(919)	catherine.stanwyck@duke.edu

Stanwyck (Project Coordinator)	VAHCS, HSRD (152) 508 Fulton St. Durham, NC 27705	6936	416-5836	
Leo Brothers (Project Coordinator)	DCRI, 200 Morris St, Durham, NC 27701	(919) 668-4601	(919) 668-7124	joseph.l.brothers@duke.edu
Jennifer Naylor PhD	508 Fulton St. Durham, NC 27705	(919) 286-0411 x133631	(919) 416-5912	jennifer.naylor@duke.edu
Cynthia Coffman, PhD (Statistical Investigator)	Durham VAHCS, HSRD (152) 508 Fulton St. Durham, NC 27705	(919) 286-6936	(919) 416-8025	cynthia.coffman@duke.edu
Avi Alkon (Programmer)	Durham VAHCS, HSRD (152) 508 Fulton St. Durham, NC 27705	(919) 286-6936	(919) 416-8025	aviel.alkon@duke.edu
Rebecca North, PhD	Duke University School of Medicine, Trent Rd. Durham, NC 27705	(919) 286-6936	(919) 416-8025	Rebecca.North13@duke.edu
Courtni France, MPH	Durham VAHCS, HSRD (152) 508 Fulton St. Durham, NC 27705	(919) 286-6936	(919) 416-5836	courtni.france@va.gov

PARTICIPATING CLINICS

Sixteen VA medical center (VAMC) primary care clinics will be randomized to be trained to implement one of two care pathways for low back pain. Both back pain care pathways being examined in this study represent best practices (not experimental interventions). This position has been officially supported by the Department of Veteran Affairs in a memorandum stating "implementing the low back pain care pathways is a non-research operations/quality improvement activity". Employees at clinical sites that implement the back pain care pathways will participate in educational training for delivering the treatment as part of standard care and clinical program-related duties only. All research activities involving human subjects (e.g., data collection and analysis) will be carried out by employees of the Durham VA Health Care System and Duke University in accordance with approved IRB protocols.

The 16 primary care clinics included in AIM-Back training activities will be selected from VAMCs agreeing to support this embedded pragmatic trial; preliminary list establishing feasibility summarized in Table 1. This group of Medical Centers provides a potential total of 39 clinics for randomization. In further consideration of site selection criteria are factors of 1) patient volume (i.e. primary care clinics will be approached to participate in the AIM-Back study if they have 800-5,000 unique patients seen for low back pain in the preceding year) and 2) distinct provider pool (i.e. they do not share providers with another VA primary care clinic that would cause pathway contamination). When these criteria are applied, we are left with 23 clinics that have potential for randomization accounting for 41,190 patients with LBP visits in 2018 (Table 1, Column 5).

These and other VA primary clinics will be approached for formal site participation agreements when the AIM-Back protocol is approved. VISN6 leadership's position has been to hold on pursuing signed site participation agreements until then. Durham (3 primary care clinics) has already verbally agreed to participation and we will continue discussion with other locations as part of "good faith" site recruitment efforts. The good faith period will allow us to rapidly pursue formal site participation agreements once the protocol is finalized.

Table 1. VA Medical Center Sites and Associated Primary Care Clinics

VAMC	Location	Facility Complexity Level	Number of Primary Care Clinics	Number of Eligible Primary Care Clinics	Total # pts with a LBP visit in 2018 from Eligible Clinics
Durham VAHCS	Durham, NC	1a	6	3	5,788
Charles George VAMC	Asheville, NC	1c	2	2	1,920
Fayetteville VAMC	Fayetteville, NC	2	2	0	0
Hampton VAMC	Hampton, VA	1c	4	1	3,675
Hunter Holmes McGuire VAMC	Richmond, VA	1a	5	3	6,008
Salem VAMC	Salem, VA	1c	3	1	2,871

W.G. (Bill) Hefner VAMC	Salisbury, NC	1c	7	5	10,775
James J. Peters VAMC	Bronx, NY	1c	4	1	1,881
Ralph H. Johnson VAMC	Charleston, SC	1c	7	7	8,992

PRÉCIS

Study Title

Improving Veteran Access to Integrated Management of Back Pain (**AIM-Back**): A Pragmatic, Cluster Randomized Trial

Objectives

The overall objective of this large, embedded pragmatic trial is to examine the effectiveness of two different care pathways for low back pain (LBP); 1) a sequenced care pathway and 2) a pain navigator management pathway. The sequenced care pathway involves three components including pain modulation with physical pain treatments (i.e., spinal manipulation, massage, or transcutaneous electrical nerve stimulation based on site availability), telephone delivered self-management counseling for increasing physical activity, and telephone delivered behavioral treatment for those identified to be at high risk for continued disability. The care management pathway involves care coordination via a pain navigator who is knowledgeable of current guideline recommended treatments for LBP. The pain navigator will engage in shared decision making with the Veteran to coordinate and sequence services based on guideline adherence by accounting for patient preference and emphasizing early use of non-pharmacological treatments.

The back-pain care pathways in this study represent best practices thus, they will be implemented into routine clinical care at participating VA clinics. As part of a companion, IRB-approved research evaluation, we will test the central hypothesis that the sequenced care pathway will reduce pain interference with normal activities and improve physical function, as measured by PROMIS Short Form scores when compared to the care management pathway. Furthermore, we will determine participant characteristics associated with greater improvements in pain and function, and better adherence to each care pathway. Planned and appropriately powered subgroup analyses for treatment moderators of previous opioid exposure and chronic vs. acute LBP will also be completed. These additional analyses will inform the potential for matching subgroup characteristics to the delivery of a specific care pathway for better clinical outcomes for Veterans.

Design and Outcomes

The AIM-Back trial was prospectively registered (NCT04411420) on June 2nd, 2020. This is a 4-year cluster randomized embedded pragmatic trial comparing the effectiveness of two care pathways for LBP. The trial will test the *central hypothesis* that patients in an sequenced care pathway will have reduced pain interference and improved physical function when compared to patients in a pain navigator pathway. We are testing these two care pathways because both have the potential to deliver guideline adherent, biopsychosocial-oriented care but they have different implementation models. For example, the sequenced care pathway is structured to include physical activity, risk stratification, and behavioral based care. However, these qualities also make this pathway more resource intensive. In contrast, the pain navigator pathway is less disruptive to existing service delivery. Therefore it is less resource intensive by taking

advantage of existing VA resources,[1] but we anticipate there will be more variability in the care received. Therefore, determining the comparative effectiveness of these two care strategies is of high interest scientifically and also for improving non-pharmacological pain management options for Veterans.

VA primary care clinics that have agreed to have their clinical staff trained in offering these care pathways as standard clinical care will be randomly assigned to one of the two care pathways. A cluster randomized trial (CRT) was selected because it offers the most pragmatic and efficient design to address our aims due to the nature of the care pathways and interest in assessing both short and long-term outcomes. An important consideration in a CRT is the optimal unit of randomization. We will designate the unit of randomization as the VA clinic (rather than an entire VAMC 'site' or 'station') to 1) enhance feasibility of implementation, and 2) improve power (allows for higher number of clusters).

Outcomes will be collected in the EHR at baseline and 3-month follow-up and for a subset of Veterans surveys will be administered at baseline with follow up points at 3, 6, and 12 months. The primary outcomes (PROMIS pain interference and physical function) and secondary outcome (PROMIS sleep quality) will be captured in templated progress notes in the VA medical record for participants (n = 1,680) using PROMIS short form instruments (4 items per scale). A subset of these same participants (n=848) will be consented for the additional survey data collection. These additional data will be used to supplement primary outcome data (see Section 9.6 Data Analyses) and in the secondary and exploratory analyses; and the measures include more detailed patient reported outcomes on pain associated distress, physical function, and health care utilization as well as longer term follow-up.

Care Pathways and Duration

The Sequenced Care Pathway (SCP) includes both on-site physical therapy (PT) services (delivered by PT providers at each participating VA clinic) and centrally-delivered services via telehealth. The SCP will be initiated by a referral from a provider at a participating primary care clinic. The first step of the SCP is contact by the central delivery provider to explain the SCP, determine preferences in delivery mode for centrally-delivered services (telephone vs video) and initiate onsite PT services. Onsite PT services will include a physical examination, and pain modulation treatment as determined by the provider that could include manual therapy or electrotherapy and capture of baseline data on pain and function. This first step will consist of a maximum of 2 on-site PT visits and it will take 2 weeks. The second step of the SCP will last 6 weeks, with patients receiving telehealth (i.e., telephone or video) calls for home physical activity instruction. The calls are will be conducted by centrally located Durham VA providers.

The third step of the SCP is referral back to the onsite PT after completion of the 6 weeks of physical activity instruction for 1 visit consisting of additional pain modulation treatments (if needed) and completion of a simple 9-question risk stratification tool (Start Back Screening Tool (SBST)). The SBST will be used to identify at higher risk for developing chronic or persistent back pain. Patients scoring as 'low risk' on the SBST are discharged from PT services, and the SCP will end with notification to the primary care clinic. Those patients scoring 'medium or high risk' on the SBST will receive 6 more weeks of SCP care consisting of weekly calls (telephone or video) from providers at the Durham VA focused on psychological and behavioral activation components, consistent with what can be delivered by a physical therapist. This third and final step of the SCP will consist of 6 treatment sessions over 6 weeks, but only for those that have been identified as medium or high risk on the SBST.

The Pain Navigator Pathway (PNP) involves use of existing non-pharmacological services available at participating VAMCs. The PNP will be initiated by a referral from a participating

primary care clinic. The first step of the PNP is telephone or video (depending on availability and common practices at the site) contact with a pain navigator. The pain navigator will be an on-site provider trained to elicit patient preferences for non-pharmacological treatments and is knowledgeable in current recommended treatment guidelines for low back pain. The pain navigator will engage in a shared-decision making process with patients to determine the appropriate nonpharmacological, guideline supported VA service for their LBP. The pain navigator will coordinate consultation of these services to increase the likelihood of receiving the interventions. Consistent with the pragmatic nature of this trial, there will be no control of the duration or intensity of treatment received in the PNP. However, we expect that receiving the first recommended service will take approximately 6 weeks. If an additional service is needed, the above process will be repeated with the pain navigator to gain referral to another non-pharmacological service. This is expected to take another 6 weeks. If additional services are requested after the second consult with the pain navigator the patient will be referred back to the primary care clinic for additional medical examination.

These two care pathways were designed to improve access to evidence supported non-pharmacological treatments for Veterans with low back pain. The care pathways have this similar goal, but the process of delivery differs. This is an embedded pragmatic trial so we expect some variation from what was described above in care delivery parameters. This variability will be captured and accounted for in study related analyses described later in the protocol (e.g. adherence related analyses).

Sample Size and Population

Our target population is Veterans seeking care for acute and chronic LBP at VA primary care clinics. Eligible Veterans will have been deemed by their primary care provider as clinically appropriate for conservative management (e.g. no trauma, fracture, infection, or cancer) and given a pathway referral for LBP management. The total projected sample size is 1,680 Veterans across 16 different clinical sites (n=105 per clinic) for the administrative sample with outcomes derived exclusively from the EHR. For the survey sample, which is approximately half of the projected administrative sample (n=848; 53 per clinic), recruitment will be balanced across the clinical sites. The 16 clinics will be randomized in equal numbers to the SCP (N=8 clinics) and PNP (N=8 clinics) arms. We plan on conducting a covariate constrained randomization[2-4] with the following clinic level covariates assessed prior to randomization: 1) average pain scale scores of LBP patients at clinic, 2) average level of opioid exposure of LBP patients at clinic, 3) number of participating primary care providers at clinic, and 4) clinic location (main medical center/community clinic), and 5) average age of LBP patients at clinic. These characteristics were chosen to represent factors likely to be associated with baseline differences in patient population that may affect primary outcomes.

1. STUDY OBJECTIVES

1.1 Primary Objective

Examine the effectiveness at 3 months of two care pathways for LBP: 1) an sequenced care pathway (SCP) and 2) a pain navigator pathway (PNP). We will test the central hypothesis that the SCP will reduce pain interference with normal activities and improve physical function, as measured by PROMIS Short Form scores when compared to the PNP.

1.2 Secondary Objectives

We will compare the effect of two different LBP pathways on sleep quality, as measured by PROMIS Short Form scores. We will also determine participant characteristics associated with greater improvements in pain and function, and better adherence to each care pathway. Planned and appropriately powered subgroup analyses for treatment moderators of previous opioid exposure and chronic vs. acute LBP will be completed. We will identify multidimensional subgroups from baseline characteristics for treatment moderation of pain and function, as well as for care strategy adherence. These additional analyses will inform the potential for identifying subgroup characteristics that indicated better clinical outcomes for Veterans in either care pathway.

Table 2. Distinctions of the Two Care Pathways

	Sequenced Care	Pain Navigator
Initiation	Provider referral	Provider referral

2. BACKGROUND AND RATIONALE

2.1 Background on Condition, Disease, or Other Primary Study Focus

A 2011 report from the National Academy of Medicine (NAM, formerly the Institute of Medicine) described chronic pain as among the most prevalent, disabling, and costly medical problems in the United States.[5] Direct cost estimates for overall healthcare expenditures for pain treatment in the US are remarkably high, and increase further when the societal cost of chronic pain is considered: including the value of lost work and lower wages, the total cost estimates range from $560 to $635 billion.[5] Recognizing the growing extent of this burden, the IOM's Interagency Pain Research Coordinating Committee (IPRCC) outlined priorities for pain research, treatment, education and policy in the National Pain Strategy (NPS).[6] In particular, it identified two over-arching priorities to 1) identify methods for reducing the incidence of acute to chronic pain transition and 2) develop effective management strategies for high impact chronic pain conditions.[7] Likewise, the 2010 - 2014 National Health Interview Survey illustrated the negative impact of chronic pain for Veterans. When compared to non-Veterans, greater proportions of Veterans reported having; 1) pain in the previous 3 months (65.5% vs. 56.4% respectively); and 2) severe pain (9.1% vs. 6.3% respectively).[8] These data and others [9,10] show an urgent need for better pain management options in the VA health system.

In addition to recognition of the burden of chronic pain, appreciation of non-pharmacological pain management as an effective, lower risk approach (as compared to common pharmacological or surgical approaches) has led multiple federal (e.g. CDC[11]) and non-federal (e.g. NAM[5] and American College of Physicians (ACP)[12]) entities to strongly advocate for broader implementation of non-pharmacological pain management. Indeed, coordinated efforts between the NIH, DoD, and VA have built the capacity for large-scale clinical research for non-pharmacological approaches by supporting a coordinating center and demonstration projects for large scale embedded pragmatic trials. These are encouraging developments for the field. However, what constitutes best practice for non-pharmacological pain management is largely unknown.[13] For example, there are still unanswered questions about structuring of non-pharmacological care pathways to optimize clinical outcomes, diminish unwarranted diagnostic testing (e.g. advanced imaging), and limit exposure to higher risk treatment pathways (e.g. opioids, injections, and/or surgery).[13]

2.2 Study Rationale

Staff Contact	Physical Therapist	Pain Navigator
Provider Approach	Directed care -<br>Pain modulation<br>Home based activity,<br>Risk stratification,<br>Behavioral/psychologically informed treatment (if indicated)	Coordinated care -<br>Patient preference<br>Guideline adherence<br>Facilitated referrals to existing VA or non-VA pain management resources
Care Progression	Sequenced	Stepped

Among conditions targeted for improved non-pharmacological pain management strategies, musculoskeletal pain, and LBP specifically, is a particular priority.[5,6] Of those affected, Veterans are more likely to have LBP or joint pain, and are more likely to report LBP or joint pain as severe when experienced.[8] The widespread impact of LBP and the disproportionate impact of LBP on Veterans' quality of life argue strongly for improvement in non-pharmacological pain management care pathways in the VA setting. Indeed, new care pathways that meet the needs of Veterans with LBP and are associated with favorable clinical outcomes represent a critical step forward in patient care.[13] To advance understanding relevant to the above priorities, we will conduct a cluster randomized embedded pragmatic trial to develop infrastructure and investigate the comparative effectiveness of two different pain management strategies that have potential to improve access to non-pharmacological care for LBP. The two pain management care pathways that will be evaluated in this trial are 1) an sequenced care pathway (SCP) and 2) a pain navigator pathway (PNP). These two strategies share the common goal of leveraging existing VA infrastructure for non-pharmacological pain management of low back pain, but the structure of care delivery is notably different in terms of *staff contact, provider approach, and care progression* (**Table 2**).

In this trial, we will determine whether these differences impact patient outcomes and/or health care utilization. Evaluating two pain care pathways in a well-powered comparative effectiveness trial will help guide infrastructure development and contribute to novel understanding of implementation and performance in different clinical settings and situations. Other significant aspects of the proposed pain management strategies, including structuring care and following recommendations for biopsychosocial care and reducing variation in care, are further discussed below.

In recognition of the high burden of pain on Veterans and associated costs to the health care system, VHA implemented a National Pain Management Strategy.[1] This Strategy approaches pain care within a biopsychosocial framework and this has led to VA offering a broad array of services for pain care. For example, VHA has specifically encouraged expansion of access to cognitive behavioral therapy for chronic pain management. However, a major barrier to organized and timely delivery of these services has been limited provider and Veteran awareness of them. Other barriers include lack of a standardized process for connecting patients to these services (e.g., who is responsible, which services are appropriate). In addition, recent legislation affecting financing of services outside of VA (e.g., CHOICE Act) has created a rapidly changing environment regarding how these services are organized, delivered, and paid. This has increased confusion about how and when pain-related services should be offered to Veterans.

VA clinics that have agreed to have their clinical staff trained in offering these care pathways as standard care will be randomly assigned to one of the two care pathways. A cluster randomized

trial (CRT) was selected because it offers the most pragmatic and efficient design to address our aims. VA clinics implementing these back-pain care pathways are not engaged in research because both pathways involve different ways of providing standard of care treatment. Employees at clinical sites that implement the back-pain care pathways will participate in educational training for delivering the treatment as part of standard care and clinical program-related duties only. Employees delivering the clinical treatment will not be directly involved in any research related procedures (e.g. extraction of data from VA's Corporate Data Warehouse or telephone surveys). To assist each site in implementing the care pathways we will collect data and provide reports to be used for process improvement. These will include both quantitative (e.g., number of eligible patients, number of referrals, etc.) and qualitative data collected from interviews with participating providers (e.g., exploring referral process and patient response to care pathways).

2.3 Sequenced Care Pathway

The Sequenced Care Pathway (SCP) provides both on-site physical therapy (PT) services and centrally-delivered services via telephone or video from study providers at the Durham VA. The SCP is initiated with a primary care referral to the pathway. Patients will start the SCP by receiving on-site PT services for 1-2 visits. These visits will include examination, pain modulation treatment, and patient education. After the on-site visit(s) patients will be referred to receive weekly calls for 6 weeks of home physical activity instruction. These calls will be conducted by providers at the Durham VA. After 6 weeks the patients will be instructed to return to the onsite PT services at participating clinics. Patients then complete a simple 9-question risk stratification tool (SBST) patients scoring 'medium or high risk' on the SBST will receive additional centrally delivered intervention of weekly calls for 6 more weeks from the providers at the Durham VA. This intervention will be tailored to include psychological and behavioral activation components, consistent with what can be delivered by physical therapists. The third step of the SCP will consist of 6 treatment sessions over 6 weeks for those that have been identified as medium or high risk on the SBST.

There will not be strict control of specific interventions delivered in the SCP. However, the SCP incorporates interventions that align with best practice for low back pain management (i.e., non-pharmacological pain treatment, early physical activity, risk stratification, and behaviorally focused intervention). Moreover, inclusion of centrally-delivered services through the Durham VA will help to ameliorate current delays in VA access to services. If there is interest from participating sites, on-site providers will be trained to take over centrally delivered components after the study period ends.

<u>Pathway Procedure for the SCP</u>

The overall flow for the Sequenced Care pathway is described in **Figure 1**, with each step described in more detail in the Figure legend. A TIDier summary[14] for the SCP has also been completed.

Figure 1. Overview and Summary of Sequenced Care Pathway

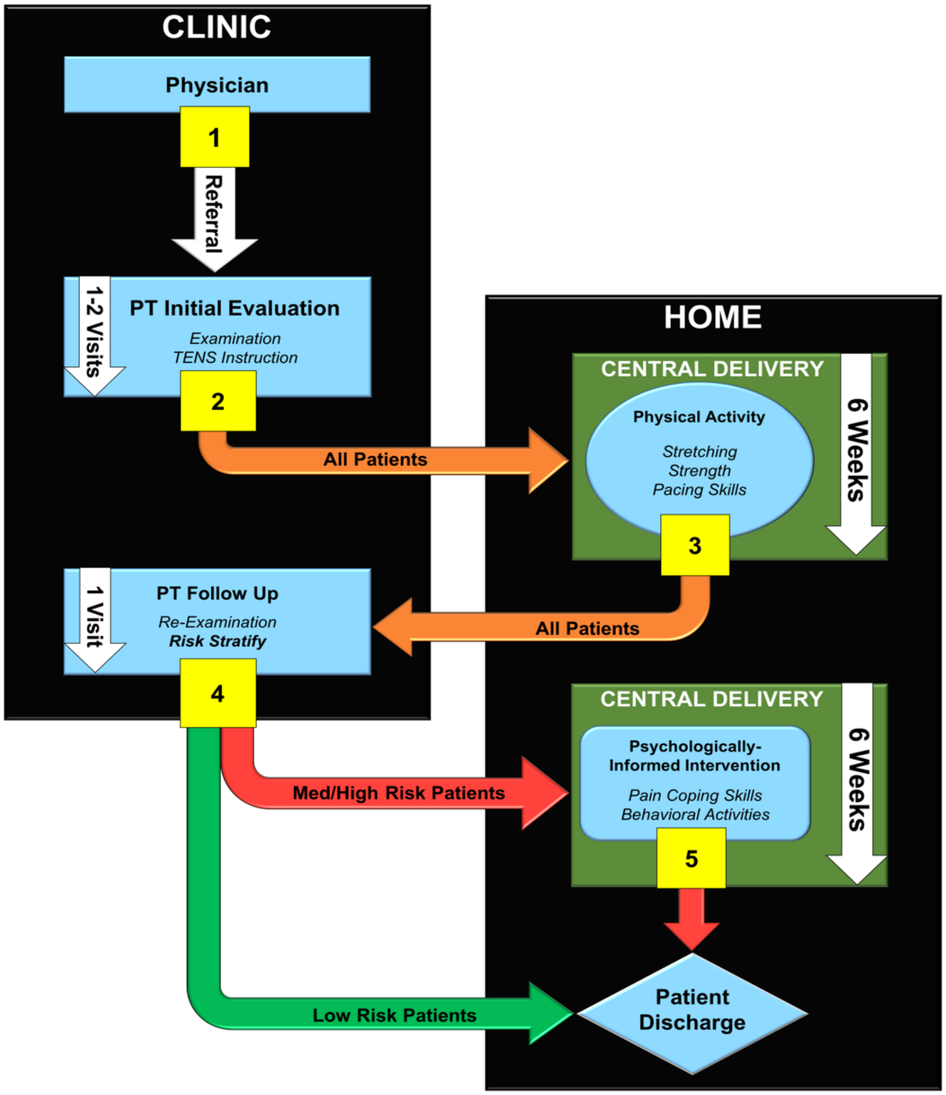

Figure Legend:

1. **1.** Patients are referred from physician to central delivery to explain the SCP and initiate onsite PT services, receive examination, on site treatment, and a transcutaneous electrical nerve stimulation (TENS) unit.
2. **2.** Patients are referred for approximately 6 weeks of centrally-delivered physical activity instruction.
3. **3.** Patients follow up with VA PT services for re-examination.
4. **4.** Patients complete the SBST. Patients who score 'low risk' are discharged to home with instructions to continue their physical activity program. Patients who score 'medium to high risk' are referred for approximately 6 weeks of a centrally-delivered psychologically-informed intervention.
5. **5.** Patients who receive psychologically-informed interventions are discharged to home upon completion.

Physical Therapy Services from Participating Sites

The physical therapy provided in the SCP will be standardized as part of the site training process. The SCP emphasizes non-pharmacological, physical pain treatment for care being delivered for at participating sites (**Figure 1, Step 2**). Keeping with the pragmatic nature of this trial, the exact type of pain treatment received will vary from site to site within allowable, evidence-based options. For this trial that will be in the form of manual therapies and/or transcutaneous electronic nerve stimulation (TENS). Manual therapies are a body-based technique that involve application of forces to patients' muscles, joints, and nerve related tissues. Each application is theoretically used to modulation pain, reduce or eliminate soft tissue inflammation, improve contractile and non-contractile tissue repair, extensibility, and/or stability, and increase range of motion for facilitation of movement and return to function. Over 200 different forms of named manual therapies exist worldwide and are used by a number of healthcare professions, including chiropractors, physical therapists, osteopaths, osteopathic physicians, and massage therapists. Manual therapies are best defined by the description of the application or technique. In this trial, we will use spinal manipulation and massage as the manual therapies of choice. TENS will be used as a separate modulatory element for physical pain treatment. TENS is the cutaneous delivery of electronic current through electrodes and has been used for pain modulation for over 50 years. TENS is included in this proposal to provide patient and provider flexibility. Spinal manipulation, massage, and TENS will be applied as described below.

Spinal Manipulation. We will consider use of a wide range of spinal manipulation techniques that fit an overall criterion: manipulation consists of a manual force applied to the spine that is accurately localized or globally applied in a single, quick and decisive movement (thrust) of small amplitude, following a careful positioning of the patient. This definition includes two forms of spinal manipulation: *Localized manipulation* (e.g., short lever manipulative procedures) involves the intent of applying a passive or assisted movement towards one specific functional region (i.e., spinal unit or single joint). These techniques are occasionally termed short lever manipulative procedures; *Generalized manipulative techniques* (e.g., long lever techniques) involve less defined pre-positioning methods and are designed to isolate the thrust to a dedicated region. Force is directed through a long lever arm, which is distant from the specific contact.

Massage. Massage methods involve the intentional and systematic manipulation of the soft tissues of the body to enhance health and healing. Multiple forms of massage techniques exist, and may include gliding, sliding, percussion, compression, kneading, friction, vibrating, and stretching. When the various forms have been compared in clinical trials, statistically significant differences among methods were found although the effects were very small.[13] As such, massage methods will be described universally as having a similar effect. Specific massage techniques for this embedded pragmatic trial will be limited to those that are most commonly used in participating clinical settings, including gliding, sliding, percussion, kneading, and stretching. Variation in specific technique is warranted because when the various forms have been compared in clinical trials, statistically significant differences among methods have been found but the effects were very small and don't suggest a superior technique exists.[13]

TENS. Results from TENS efficacy studies among individuals with chronic pain conditions have not been uniform.[15-17]. However, in many of these trials TENS delivery was likely suboptimal.[18] Recent animal models have expanded our understanding of TENS mechanisms. A key finding was that certain TENS frequencies activate the descending pain inhibitory system at the midbrain and spinal cord; including the periaqueductal gray, rostroventral medulla, and spinal cord dorsal horn.[19-22] Moreover, TENS activates mu-opioid receptors, which are the same receptors responsible for pain relief through pharmacological pain management.[21,23,24] In this embedded pragmatic trial, we will utilize TENS delivered at current evidence-based recommendations, which is asymmetrical biphasic alternating current at high frequency (20-

150Hz) and high intensity (verbalized by patient as “strong but tolerable” and not noxious).[18,25,26]. Moreover, sensory perception changes with age, meaning older adults require higher intensities to experience similar pain reduction as younger adults and we will ensure our dosing parameters adjust for Veteran age.[27]

Home Based Treatment from Durham VA Providers. A key component of the SCP is a comprehensive, home-based 6-week physical activity program (**Figure 1, Step 3**). This is an integral part of the program because physical activity is a core component of managing LBP over the long term.[28,29] In addition, given the chronic nature of this condition, it is essential that individuals learn skills for continuing with a physical activity program in the absence of regular clinical oversight. Therefore, this part of the program emphasizes development of skills in goal setting, exercise progression, and dealing with barriers and setbacks in continuing a physically active lifestyle, particularly in the context of LBP.

Prior research has not identified one specific physical activity program as being superior for individuals with LBP. Rather, based on prior studies, LBP guidelines simply recommend that a physical activity program for individuals with LBP should be comprehensive and include stretching, strengthening, and endurance activities.[28,29] Therefore the physical activity program of this pathway will include all of these elements. This component of an sequenced care pathway will be telephone or video-based coaching; this is a delivery approach we have employed successfully in other studies of Veterans with chronic pain conditions.[30-32] Specifically, we will be following the protocol of a home based, telephone supported physical activity program that was successfully piloted by Goode et al for older adults with low back pain.[33]

Risk Stratification. After completion of the 6-week program there is an opportunity for tailored treatment based on risk stratification by the SBST (**Figure 1, Step 4**). Risk stratification will be completed by the on-site PT on return to clinic visit. The SBST is a simple, but effective, risk stratification method that can be scored quickly (summing 9 items) to derive a low, medium, or high risk classification.[34] SBST use is consistent with cutting edge methods for identifying patients with LBP who are unlikely to recover unless they receive modified treatment with a behavioral and/or psychological focus. Furthermore, the SBST is better than or comparable to individual psychosocial questionnaires that have traditionally been used for establishing prognosis.[25,35] The onsite PT will apply SBST risk stratification based after the home based physical activity is completed because this approach that improves prediction accuracy and reduces the number of participants unnecessarily identified to be in need of behaviorally focusedtreatment.[36] Those at low risk from the SBST screen will be discharged from additional services, and recommended to follow up with the original referring source (**Figure 1, Step 6**).

Those at medium to high risk will receive behaviorally focused treatment that incorporates key psychological principles to encourage continued activation (**Figure 1, Step 5**). This integrated approach of combining physical and psychological treatments is appropriate for physical therapists to deliver, and the treatment provided will be consistent with the scope of practice for a physical therapist. Indeed Drs. George and Keefe have completed such training for several other clinical trials involving non-psychologist providers delivering psychologically informed treatments. This part of the pathway will continue with weekly calls for 6 weeks. The overall goal of the tailored behavioral treatment is to reduce pain-related fear and threat associated with LBP, as well as to discourage long-term avoidance behavior and encourage activation. This team has extensive experience with randomized and embedded pragmatic trials investigating the efficacy and effectiveness of combined approaches (e.g., physical therapy combined with pain coping skills). The nature of the activities will change however intentionally shifting to graded exercise, improving self-efficacy, and enhancing pain coping skills. This shift in treatment philosophy is warranted for the patients that remain medium or high risk after the physical activity phase. The overall goal of this part of the SCP is to reduce the risk of

continuing disability from LBP while emphasizing the need for self-management. After completing the behaviorally focused treatment the Veteran will be discharged from the SCP and recommended to follow up with the original referring source.

2.4 Pain Navigator Pathway

The Pain Navigator Pathway (PNP) involves referral of patients to a **pain navigator** who is knowledgeable in current recommended treatment guidelines for low back pain. The pain navigator engages in a shared-decision making process with patients to determine the appropriate guideline supported VA service for back pain. The pain navigator will also help to coordinate consultation of services with the referring physician.

Pathway Procedure

The overview of the care management pathway is described in **Figure 2**, with each step describe in more detail in the Figure Legend. A TIDier summary[14] for the PNP has also been completed.

Figure 2. Overview of the Pain Navigator Pathway

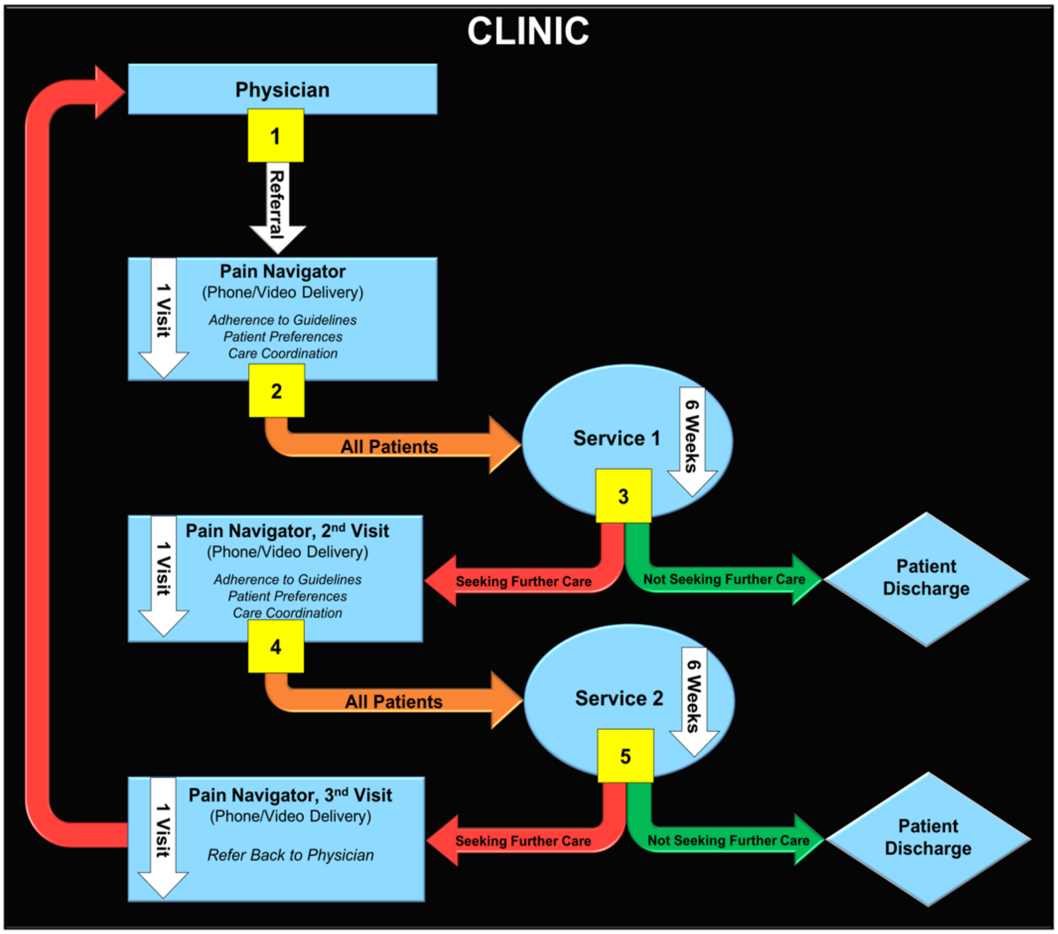

<u>Figure Legend:</u>

1. Patients are referred from their physician to a clinic pain navigator. Pain navigator contacts patients via telephone or video conference. Pain navigator and patient engage in a shared-decision making process to identify the appropriate VA service for patient's low back pain.
   - Pain navigator provides information on current VA recommended guidelines for non-pharmacologic and non-surgical pain management; as well as availability of services
   - Patient provides their services preferences

2. Pain navigator coordinates consultation input with physician. Patient attends service.

3. At the completion of service, patient either do not seek further care and are discharged to home, or seek further care through the pain navigator.

4. Patients who seek further care through the pain navigator re-engage in the share-decision making process to identify the next appropriate VA service. The pain navigator coordinates consultation input with physician, and patient attends a second service.

5. At the completion of service, patient either do not seek further care and are discharged to home, or seek further care. Those seeking further care follow up with the pain navigator who coordinates referral back to the physician.

Rationale for Pain Navigator Pathway

VA has adopted the Stepped Care Model for Pain Management (SCM-PM)[1] and this is the model of care that the PNP is designed to deliver. The SCM-PM is an evidence-based model that calls for initial assessment and management of health problems via low intensity interventions, delivered in the context of Patient-Aligned Care teams (VHA's version of the patient-centered medical home model). Expanded Care Management in Patient Aligned Care Teams (PACT) is recommended as an initial approach to pain care; however, there has been little guidance for how PACT teams should accomplish this. Thus, the PNP will implement and test a direct recommendation of VA's SCM-PM by training a pain navigator to facilitate access to VA and non-VA pain services.

Pain Navigator. The pain navigator, often a member of the PACT, will contact patients by phone or video within one week of referral. Using standardized protocols for assessing clinical appropriateness and eliciting patient preferences, pain navigators will provide information and guidance for patients to facilitate access to existing VA and non-VA resources for LBP care that is consistent with their preferences and recent practice guidelines for LBP (**Figure 2, Step 2**). Pain navigators will conduct up to a maximum of three calls with patients over a 3-month period depending on what is needed or recommended; with the first call to survey recommended services, elicit Veteran preference, align with guidelines, and initiate referrals. The pain navigator will also have a role in addressing any encountered barriers to access and encourage engagement and adherence with recommended services. Consistent with the SCM-PM, pain navigators will initiate referrals sequentially and only refer to subsequent programs or services (up to a maximum of 2) if the Veteran has not improved. Veteran improvement will be based on two factors persistent pain and/or physical limitation and indication of willingness to seek additional care for LBP symptoms. After 2 services without improvement the Veteran will be referred to the physician that initiated the PNP referral (care pathway).

Services. The specific services the Veterans receive in the PNP are not to be controlled by the pain navigator. Instead the pain navigator will use general principles of guideline adherence and patient preference to coordinate care. It is beyond the intent of this embedded pragmatic trial to have strict restrictions on the pain management services received (including additional diagnostic tests). This is an intentional part of the design, and instead of controlling the services received, we will be collecting information on the type of treatments and procedures that were part of the care episode for low back pain. For more details see Section 5.3 (Concomitant Interventions) and see Section 6.0 (Study Procedures) for the templates that will be used to create Health Factors that will collect specific information on patient encounters from the electronic medical record for those in the PNP.

3. STUDY DESIGN

An overview of this 4-year cluster randomized embedded pragmatic trial is presented in **Figure 3** and SPIRIT Table[37] has been completed. We will compare the effectiveness of two care pathways for LBP. The trial will test the *central hypothesis* that patients in the SCP will have reduced pain interference and improved physical function compared to patients in the PNP. We are testing these two care pathways in a comparative effectiveness approach because both have the potential to deliver guideline adherent, biopsychosocial-oriented care but they have different implementation approaches.

VA primary care clinics that have agreed to have their clinical staff trained in offering these care pathways as standard care will be randomly assigned to one of the two care pathways. A cluster randomized trial (CRT) was selected because it offers the most pragmatic and efficient design to address our aims due to the nature of the care pathways and interest in assessing both short and long-term outcomes. An important consideration in a CRT is the optimal unit of randomization. We will designate the unit of randomization as the VA clinic (rather than an entire VAMC 'site' or 'station') to 1) enhance feasibility of implementation, and 2) improve power (higher number of clusters).

Figure 3. Study Design Overview

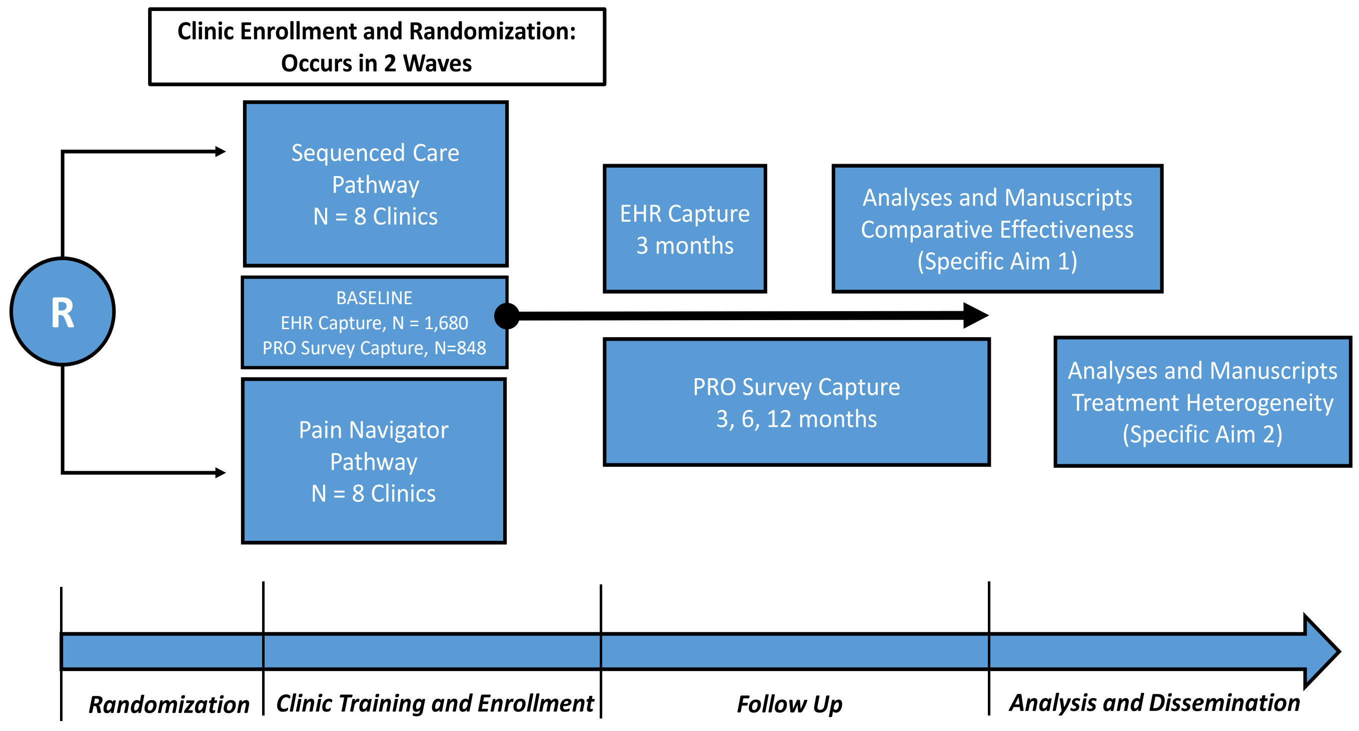

Veteran outcomes will be collected in the EHR at baseline and at 3-months follow up and for a subset of Veterans, surveys will be administered at baseline with follow up points at 3, 6, and 12 months (see Section 10 - Measures and Data Collection). The primary outcomes (PROMIS short forms for pain interference and physical function, 4 items each) and secondary outcome (PROMIS short form sleep quality) will be captured in the VA medical record by clinical providers using templates provided by the research team (see Section 6 - Study Procedures). Veteran participants (n=848) will be consented for the additional survey data collection by Durham VA study staff .

This trial is purposefully powered to examine treatment effect moderation for care strategy responses in select patient subgroups. Many clinical trials find negligible or small treatment effects for LBP interventions, so identification of treatment moderators (i.e. factors that predict largest benefit from intervention) is important for a better understanding of how treatment could be administered at the patient level. This is a newer area of emphasis in LBP research and there are methodological[38] and statistical[39] challenges to investigating treatment moderation. We have mitigated many of those challenges by taking an *a priori* approach that focuses our treatment moderation analyses to 1) chronic vs. acute LBP and 2) previous opioid

use vs. opioid naïve. This is an embedded pragmatic trial so these two subgroups were selected for their clinical relevance (i.e., can be easily identified during routine encounters) and because their potential as treatment moderators has been established in the literature. Indeed a 2015 meta-analysis on this topic included LBP status and narcotic medication use as potential treatment moderators with strong statistical evidence ($p < 0.05$) supporting their inclusion in confirmatory analyses.[38] We hypothesize that we will observe better outcomes from the SCP for patients with chronic pain and opioid exposure based on previous studies.[38,40,41] Including planned subgroup analyses in this embedded pragmatic trial will complement the primary trial results by providing important information to guide decisions about optimal targeting of resources. Moreover, by focusing on subgroups that have been prioritized in the scientific literature, these analyses also advance the field in this emerging area.

4. SELECTION AND ENROLLMENT OF CLINICAL SITES AND PARTICIPANTS

This study is a 4-year cluster randomized embedded pragmatic trial comparing the effectiveness of two care pathways for increasing non-pharmacological treatment options for LBP. The study target population is veterans seeking services for lower back pain in participating VA clinics. There will be selection of clinical sites and participants for this trial.

Clinical Site Selection

On May 16th we met with VISN6 leadership to distribute an update on the AIM-Back trial and being the process of recruiting interested clinical sites. Formal site agreements will be pursued once the study protocol is finalized.

Unit of randomization: VA primary care clinics.

Sample size: 16 clinics (8 clinics randomized to each care pathway).

Clinic eligibility:

- Volunteer and return signed clinic participation agreement
- Availability of clinical personnel willing to deliver the treatment interventions (in either arm).
- Staff and location need to be distinct from other enrolled clinics.
- Clinics can have a variable number of providers, but together meet criteria for range of monthly visits for LBP

4.1 Participant Inclusion Criteria

At the level of the individual, participant eligibility criteria are intentionally broad in keeping with the pragmatic nature of this trial. Eligibility criteria will be applied equally to and are appropriate for individuals seeking care at any of the clinical sites. Site training will include instruction in the referral criteria so that providers have consistency in the selection of participants for the care pathways (see below). Our intent is to: 1) examine the care pathways in the way they would be used in clinical practice; and 2) maximize diversity and the potential to generalize the findings from this trial.

Referral Criteria (part of site training and used by providers for pathway determination)

1. LBP that is localized or radiating
    - Localized = symptoms reported in the T12 to S5 region only, occurring unilaterally or bilaterally

- Radiating = localized symptoms reported in the T12 to S5 region, occurring unilaterally or bilaterally and radiating symptoms to the buttocks, thigh, or lower leg

2. LBP that is appropriate for conservative care
    - No history of recent spinal trauma or surgery (i.e. past 3 months)
    - No signs or symptoms suggestive of spinal fracture, tumor, or infection

Specific inclusion criteria for inclusion in the analyses are listed below.

Inclusion Criteria (must meet all)

1. Age >= 18
2. Seeking care for LBP with or without radiating symptoms from a participating VA primary care clinic
3. Provider determines LBP is appropriate for conservative management
4. Referred to integrated SCP pathway or PNP pathway by participating clinic provider

4.2 Participant Exclusion Criteria

Narrower eligibility criteria would exclude a significant number of patients with LBP resulting in limited application of examined care pathway models. Some patients will not meet clinical criteria for all treatments within each pathway; however, they will be included in the evaluation to be consistent with our pragmatic goals of determining the average treatment effect of each arm for patients with LBP when receiving routine clinical care. All adult subjects (not referred for hospice or palliative care) that are enrolled in a participating VA primary care clinic and are referred to one of the LBP pathways will be eligible for the main analysis.

Exclusion Criteria (if meet any)

1. Receiving or referred for hospice/ palliative care (defined by encounter codes and CPRS consults)
2. No documented phone number in the electronic health record.

4.3 Study Enrollment Procedures for Outcome Collection

Enrollment procedures. We anticipate total sample size of 1,680 Veterans for the administrative sample (main analysis). For these participants, data will be obtained from VA administrative and electronic health records.

We will recruit a subset of Veterans (n=848) to participate in a series of telephone interviews. The selection of this subset is described in the subsequent section (Screening, Informed Consent, Baseline Data Collection). Because these participants will be asked to provide additional information over and above what is collected and stored in the electronic health record as part of routine clinical care, we will seek informed consent for participation in this portion of the study, using the following procedures. The additional inclusion/exclusion criteria for the telephone subset are:

Inclusion Criteria for Telephone Survey Subset (must meet all)

1. Valid phone number in medical record
2. Able and willing to provide informed consent

Exclusion Criteria for Telephone Survey Subset (if meet any)

1. Currently in institutional care (nursing home or hospital).
2. Cognitive impairment or dementia (identified via ICD diagnosis codes or PCP note in previous 2 years) or lack decision-making capacity, documented in the medical record.
3. Serious mental illness defined as diagnosis of schizophrenia, bipolar disorder, psychiatric hospitalization in the previous year or current high-risk suicide flag in their CPRS medical record.
4. Unable to communicate on the telephone, or no telephone access for duration of study

Subject Recruitment. At the time of referral to an AIM-Back clinical care pathway for LBP, primary care providers will also introduce the patient to the research study portion of the AIM-Back project and ask the patient for his/her permission to be contacted by the research team. Referring providers will be given talking points from the study team about the research study including that participation in the research study is completely voluntary. The permission to be contacted will be communicated to the research team via the CPRS consult at each participating site. The AIM-Back consult will contain a mandatory "yes/no" check box for the PCP to indicate the patient's preference for research contact. This method of recruitment will allow the research team to contact patients who have not opted out soon after their referral to the AIM-Back pathway, facilitating the timing of the baseline assessments.

We have opted not to enroll Veterans in person because phone interviews improve feasibility given the number of clinics (n = 16), their expected geographic dispersion, and the overall number of participants that will need to provide consent for survey contact (n = 848). In addition, enrolling by phone is least burdensome for patients and allows us to reach patients whose clinic visit may have occurred on a weekend or during evening hours. All VAMC primary care clinics now offer some expanded hours.

Screening, Informed Consent, Baseline Data Collection. Durham VA study staff will conduct a weekly data pull that identifies Veterans that have been referred to the pathway at enrolled clinics who meet eligibility criteria and have agreed to be contacted for the survey at their referral to the AIM-BACK program. We will randomly sort eligible participants, stratified by clinic, and the RAs will recruit from this list for the Telephone Survey Subset. Before contacting each patient for enrollment, RAs will perform targeted chart review to confirm eligibility and then contact Veterans to screen for additional exclusion criteria not always identified in the medical record (e.g. hearing impairment or hospice referral) and ask them if they wish to participate. Because the study is entirely telephone based, waiver of written informed consent has been granted by the Durham VAMC IRB. After obtaining verbal informed consent, the Durham VA RA will collect baseline survey data from all study participants. Study staff will attempt to contact Veterans for enrollment within 2-3 days of the data pull date and make up to 10 attempts to contact each potentially eligible subject.

Telephone Surveys Follow-up.

Survey participants will have provided verbal informed consent prior to baseline survey collection to allow for additional contact from the Durham VA study staff for the follow-up surveys at 3,6, and 12 months.

Data collected via telephone interviews will complement the data obtained via EHR with patient-reported data across multiple domains of pain and associated co-morbidities. These data will

also provide rich information about response trajectories and suggest areas to target future EHR data capture methods. This approach ensures the trial will build capacity for conducting large-scale pragmatic clinic trials with the VA health care system.

5. IMPLEMENTATION OF CARE PATHWAYS

VA clinics that have agreed to have their clinical staff trained in offering these care pathways as standard care will be randomly assigned to one of the two care pathways. A manual for training materials used for participating VA clinics has been drafted. VA clinics implementing these back-pain care pathways are not engaged in research because both pathways involve different ways of providing standard of care treatment. Employees at clinical sites that implement the back-pain care pathways will participate in educational training for delivering the treatment as part of standard care, clinical program-related duties only.

AIM-Back will employ implementation facilitators to work with clinics to implement and maintain their care pathways. AIM-Back facilitators will work directly with a Point of Contact selected by each site to develop procedures for keeping the care pathways and referral procedures top of mind for referring providers, using existing infrastructure at each clinic (e.g. staff meetings). Employees delivering the clinical treatment will not be directly involved in any research related procedures (e.g. follow up survey collection over the phone). To assist each site in implementing the care pathways we will collect data and provide reports to be used for process improvement. These will include both quantitative (e.g., number of eligible patients, number of referrals, etc.) and qualitative data collected from interviews with participating providers (e.g., exploring referral process and patient response to care pathways).

5.1 Qualitative Data Collection

In-depth, semi-structured, individual telephone interviews (~20-30 minutes each) with be conducted with a sample of the participating, referring providers. We will purposefully sample approximately 2 referring providers from each clinic as a function of low/high adoption as evidenced by number of referrals at 2.5 to 3 months (based on clinic start dates). Interviews will be conducted as close as possible to the 3-month time frame (+/- 2 weeks to allow for scheduling). A structured form for notetaking will be utilized in order to facilitate rapid qualitative analysis. Information obtained will be used to create case memos (case study qualitative design), which will be fed back to the clinics and triangulated/supplemented with process improvement notes from the facilitator. We will target providing this feedback to the clinics by around the halfway point of the 9 months (i.e., 4.5 months). Information obtained will also inform the next starting clinics with an evaluation across all cases (multiple case study design) to be conducted at the conclusion of the active study/pathway period. The interview guide will be informed by the Consolidated Framework for Implementation Framework (CFIR)[42] and include in-depth, descriptive questions on barriers and facilitators at multiple levels (i.e., provider, clinic, facility, and patient) from the perception of the referring providers. All qualitative data will be used for quality improvement purposes (i.e., will not be considered human subjects research).

5.2 Concomitant Interventions

We have taken steps to minimize the risk of cross-contamination through our choice of study design and by imposing restrictions on clinic participation. That is, clinics must be located at geographically distinct locations, rather than on the same campus. We will measure and report the number of patients with referrals to both LBP care pathways. Furthermore, evidence of concomitant diagnostic testing and treatments received outside of the care pathways will be monitored via the VA electronic record for both LBP care pathways. Finally, we will be implementing templates (Section 6.0 Study Procedures) to capture key study outcomes as health factors and also collecting health care utilization data from both care pathways to track all intervention usage and adherence to intervention. Because this is an embedded pragmatic trial we can't limit use of concomitant interventions, but we will be able to track usage rates and compare across the two care pathways.

5.2 Adherence Assessment

We will examine "adherence" among all patients referred to one of the clinical pathways. We define "adherence" according to patient participation in planned sessions with intervention team personnel (telehealth or in-person) in either the SCP or the PNP depending on participating clinic. Data for participation/adherence to SCP sessions and referrals by the pain navigator in PNP arm will be captured in progress notes entered into the EHR and completion of referred services to PNP will be captured in the EHR. The number of planned sessions will vary between and within arms (e.g. depending on response to 1st service in PNP and depending on risk stratification in SCP); therefore, to define an adherence measure that may apply to both arms for descriptive purposes we will *a priori* define adherence as follows: 1) attended 1 planned session only; 2) partially adherent – attended some, but not all planned sessions; 3) attended all planned sessions.

We are defining adherence this way *a priori* because the number of referrals/sessions for Veterans to adhere to can vary greatly depending on the intervention. For the SCP intervention, the number of referrals or sessions to adhere to depend on risk stratification using the SBST (1-9 for low-risk veterans and 1-15 for high risk veterans). For Veterans in the PNP arm, the maximum number of referrals to adhere to can range from 1 to 5depending on what referrals (if any) are made by the Pain Navigator (**Table 3**).

Our *a priori* three-level categorization for adherence is equitable between arms in the upper and lower categories, but the middle category could be very heterogeneous and not informative. Therefore, we will describe adherence in both care pathways separately in order to identify patient characteristics potentially associated with program participation and improved pain outcomes. We will not test for differences in adherence between pathways as our goal is to gain insight into where to target clinical programs and focus efforts to improve access to and patient engagement with non-pharmacologic pain services. Depending on the distribution of the number of sessions in each pathway, we will explore additional multi-level variables that may be more informative within arms. Within SCP and risk groups, we may be able to use more granular definitions (e.g., continuous measures) for examining the impact of adherence on outcomes within treatment arm and/or risk group.

The structure of the adherence variable for Aim 2 analyses will depend on the empirical data distribution to determine adherence categories for each arm. For the PNP arm the *a priori* definition above may be reasonable, however for the SCP arm more granular categories by risk groups should be possible (see Table 3).

Data Collection for Adherence

For both PNP and SCP, adherence outcomes in Table 3 for each participant will be captured in the EHR via referral consults to pathways and health factor data in EHR templates used by clinic personnel in each of the pathways.

1. For the Pain Navigator Pathway, the numerator can range from 1 to 5 (to be enrolled in program has to adhere to referral to PNP) and the denominator is 5 so percentage of adherence for patients can range from (20%-100%)
   a. Patient is referred to the navigator and attends PNP baseline visit (adhere=1)
   b. Pain navigator may refer to service - referral to one or more services (adhere =1), may not refer to any services (adhere=0)
   c. Pain Navigator 6-week follow-up visit – patient attends visit (adhere=1), patient does not attend visit (adhere=0)
   d. Pain navigator may refer to service - referral to one or more services (adhere =1), may not refer to any services (adhere=0)
   e. Pain Navigator 3-month follow-up visit – patient attends visit (adhere=1), patient does not attend visit (adhere=0)
2. For the Sequenced Care Pathway (screen to low risk using Start Back Screening Tool at the PT follow-up visit or do not attend PT-follow-up visit), the numerator can range from 1 to 9 (to be enrolled in program has to adhere to referral to SCP) the denominator is 9
   a. Patient is referred to PT and attends PT baseline visit– (adhere=1)
   b. Home central delivery, physical activity focus – 6 weekly sessions; for each session either attends (adhere=1) or does not attend adhere=0.
   c. PT follow-up – patient can adhere or not adhere to that follow-up
   d. Home central delivery 3-month follow-up visit – patients attend visit (adhere=1), patient does not attend visit (adhere=0)
3. For the Sequenced Care Pathway (screen to medium or high risk Start Back Screening Tool at the PT follow-up visit), the numerator can range from 2 to 15 (to be in this group have to enrolled and have to attend PT follow-up visit to get stratified) the denominator is 15
   a. Patient is referred to PT and attends PT baseline visit– (adhere=1)
   b. Home central delivery, physical activity focus – 6 weekly sessions; for each session either attends (adhere=1) or does not attend (adhere=0).
   c. PT follow up - attends follow-up visit (adhere=1)
   d. Home central delivery, pain coping and behavioral focus - 6 weekly sessions; for each session either attends (adhere=1) or does not attend adhere=0.
   e. Home central delivery 3-month follow-up visit – patients attend visit (adhere=1), patient does not attend visit (adhere=0)

Table 3. Adherence Metrics and Definitions for Each Care Pathway

Intervention	Sessions	Adhered (Yes/No)	Adherence outcome	Notes
**PNP**	Initial referral only-PNP baseline visit	Yes	1/5	
	Referral for 1or	Yes	2/5	

	more services			
	No referral to services	No	1/5	
	PNP follow-up visit	Yes	2/5 or 3/5	
		No	1/5 or 2/5	
	Referral for 1 or more services	Yes	3/5 or 4/5	
	No referral to services	No	2/5 or 3/5	
	PNP 3-month visit	Yes	2/5 – 5/5	
		No	1/5 - 4/5	
**SCP**	Initial referral to 1st PT visit	Yes	1/9	
	Home-based delivery sessions	Yes	2/9 to 7/9	May not adhere to all 6 sessions; to get here had to adhere to 1 (initial referral)
		No	1/9	Adhere to initial referral but did not complete any home based sessions
	PT follow-up visit	Yes	1/9 to 8/9	
		No	1/9 to 7/9	If do not adhere to 2nd PT visit do not know risk group
Low risk only or did not attend PT follow-up visit	SCP 3-month visit	Yes	2/9 – 9/9	
		No	1/9 - 8/19	
Medium or High risk only	CBT telehealth delivery sessions	Yes	3/15 to 14/15	May not adhere to all 6 sessions; to get here had to adhere to initial referral and PT follow-up visit
		No	2/15 to 8/15	

	SCP 3-month visit	Yes	2/15 – 15/15	
		No	1/15 - 14/15	

6. STUDY PROCEDURES

An overview of the Study Procedures is included in a completed SPIRIT Table[37]. This is embedded pragmatic trial that involves providers inputting data collected during routine clinical encounters for LBP in VA clinics that have participated in the educational sessions for delivering the care pathways. These clinical providers are not research staff. These EHR templates for data collection will allow for retrieval of data from the Corporate Data Warehouse so that study related analyses can be performed.

6.1 EHR Data Collection Templates

As our primary outcomes are collected by clinical providers in the EHR, we will have study procedures in place to help providers with timing of outcome ascertainment (see outcome ascertainment study flow diagram in Figure 4). For baseline outcome assessments, biweekly reports will be generated for initial intervention contact and baseline EHR outcome collection by the Pain Navigator in the PNP arm and SCP centralized study personnel. Similarly, biweekly reports will be generated with a list of eligible patients for the 3-month assessment and provided to PNP Pain Navigator or SCP centralized personnel. The study team will monitor outcome ascertainment timing at each participating clinical site to trouble-shoot any problems or issues to circumvent loss of follow-data. Interim EHR data will be collected during routine intervention contact

Figure 4. AIM-Back Procedure for Ascertainment of Primary Endpoint

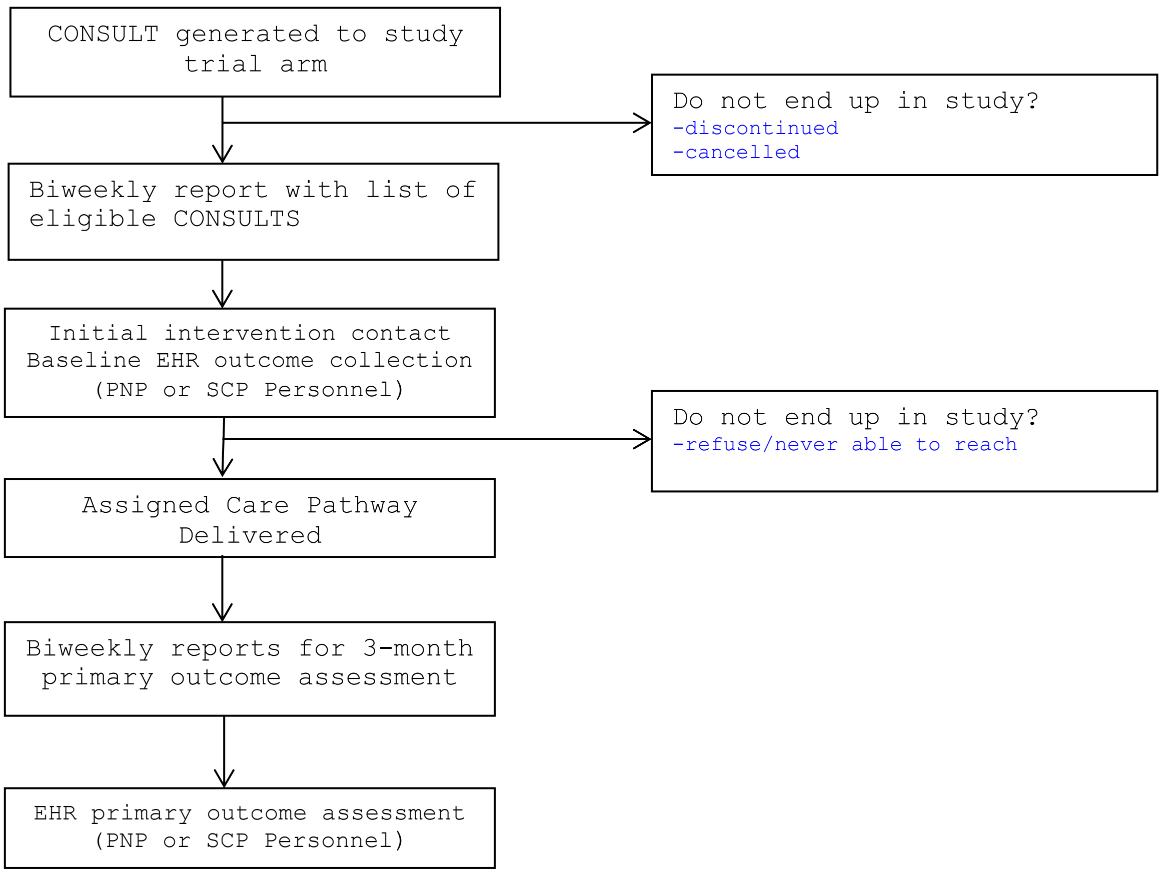

The information collected is also summarized below in **Tables 4 and 5**.

6.1.1 Sequenced Care Pathway

Table 4. Summary of Variable Fields Collected by Providers for Sequenced Care Pathway

Template	Clinical and adherence variables	PROMIS*	Risk Stratify	Provider Action
**Central Delivery Initial Phone Call Data Collection Template**	CDC Pain<br>NIH Back Pain<br>Adherence	Physical Function<br>Pain Interference<br>Sleep Quality	Start Back Screen	Initiate PT Evaluation<br>Describe SCP intervention
**SCP-PT Intervention Template Visit**	CDC Pain<br>NIH Back Pain<br>Adherence	Physical Function<br>Pain Interference<br>Sleep Quality	Start Back Screen	Pain Modulation Education<br>Central Delivery Referral
**Central Delivery Physical Activity Phone Call Template**	Adherence			Review and Modify Home Exercise Program<br>Goal Setting and Action Planning<br>Educate on Pace Setting

				Skills
**SCP-PT Intervention Follow-up Visit**	CDC Pain<br>NIH Back Pain<br>Adherence	Physical Function<br>Pain Interference<br>Sleep Quality	Start Back Screen	Pain Modulation Education<br>Low Risk - Discharge<br>Med/High Risk - Referral
**Central Delivery CBT Medium / High Risk Intervention Phone Call Template**	Adherence			Pain Coping Skills<br>Behavioral Activities
**Central Delivery 3-month follow-up Phone Call Data Collection Template**	CDC Pain<br>NIH Back Pain<br>Adherence	Physical Function<br>Pain Interference<br>Sleep Quality		

Key - * = primary outcome collection through the health factor via the electronic health record

6.1.2 Pain Navigator Pathway

Table 5. Summary of Variable Fields Collected by Pain Navigator

Template	Clinical and Adherence	PROMIS	Navigator Action
**Pain Navigator Care Pathway Initiation and Baseline Data Collection Template**	CDC Pain<br>NIH Back Pain<br>Adherence	Physical Function<br>Pain Interference<br>Sleep Quality	Service Referral 1<br>Guideline Review<br>Patient Preferences
**Pain Navigator Care Pathway Update Call Template**	Adherence		Service Referral 2<br>Guideline Review<br>Patient Preferences
**Pain Navigator Care Pathway 3-month Follow-up Template**	CDC Pain<br>NIH Back Pain<br>Adherence	Physical Function<br>Pain Interference<br>Sleep Quality	Services Received Summary<br>Referral to Initial Provider<br>Pathway Completion/Discharge

Key - * = primary outcome collection through the health factor via the electronic health record

6.2 VA Corporate Data Warehouse Collection

Collection from VA Corporate Data Warehouse will include:

Medication use. Outpatient and inpatient files from the VA Corporate Data Warehouse will be used to evaluate all visit data and associated diagnosis codes. Pharmacy Benefits Management (PBM) outpatient records will be used to define medication outcomes. PBM includes medication information including date dispensed, VA product name and VA drug class, generic drug name, national drug code, prescriber identifiers, and quantity and days' supply dispensed

Imaging, providers, and procedures. We will measure utilization of LBP imaging, provider visits, and selected procedures according to procedures employed in prior studies. [43-46] Following processes used in prior studies from Drs. Goode[46] and George,[43] we will use ICD codes to classify diagnostic/anatomical location and CPT codes to classify resource use within 12 months of referral to either LBP care pathway. For resource use we will measure and report imaging (total number of imaging studies of the lumbar spine region including plain radiographs, magnetic resonance imaging (MRIs), and computed tomography (CT) studies), provider visits (back pain and non-back associated), procedures (epidural steroid injection, facet injection, and radio frequency ablation), surgery (fusions, discectomy, laminectomy, and mild procedures).

ED visits and Pain Related ED Visits. Visits for back pain related disorders to the emergency department are common (i.e., 2.6 million visits per year) in the U.S.[47] We will measure ED visits for low back pain and general pain (any type) to the VA and non-VA facilities using the VA Corporate Data Warehouse (CDW) and Non-VA fee or ICD codes at 12-months since enrollment. Dr. Hastings and her team have validated strategies for identifying ED visits (both VA delivered and VA-financed ED care) in national VA administrative data and assigning reasons for visits using ICD codes.[48] A summary of measures, definitions, data sources and time points is provided in Section 10.

6.3 Blinding

Given the pragmatic design, participants and treating providers will not be blinded to the care pathway. **Table 6** below summarizes blinding status for various study team members and also treating providers. Treating providers delivering the clinical pathways at a clinic and the Veteran patients in that pathway have no knowledge or training on the comparison pathway being delivered at other clinics as part of this study. Treating providers entering health factor data in the EHR will not be blinded. However, survey outcome assessors (RA conducting telephone surveys) will be blinded to care pathway received. Study statisticians cannot be blinded to clinic randomization arm as they will be monitoring data collected in the EHR to track study flow, provide feedback to clinics and identify barriers or problems. Because the data entered in the EHR is different due to the nature of each of the interventions, it is not possible for study statisticians to be blinded in the conduct of this study. Study statisticians will be generating the randomization tables using a covariate constrained randomization and provide a report to inform clinics of their randomization arm.

Table 6. Blinding Summary for AIM-Back Trial

Study personnel	Clinic level randomization blinding status	Clinic level blinding status justification	Individual patient level data access	Individual patient level data access notes
PIs	Unblinded	Site recruitment and engagement role, troubleshooting barriers to clinical intervention delivery of programs; management of centralized intervention delivery	No	Access to aggregated patient data presented in reports for tracking and troubleshooting
Research coordinator	Unblinded	Site recruitment and engagement and monitoring of delivery of clinical intervention	No	Access to aggregated patient data presented in reports for tracking and troubleshooting
VA research assistant	Blinded		Yes	Administering patient surveys
PhD Statistician	Unblinded	Involved in monitoring data in EHR including assessing targeted sample size goals; conducting and verifying covariate constrained randomization	Yes	Access intermittently to troubleshoot any problems with recruitment, data collection and data quality
Masters Statistician	Unblinded	Involved in extracting and monitoring data from EHR and developing reports; conducting and verifying covariate constrained randomization	Yes	Ongoing access to extract and monitor data from EHR and/or patient surveys
SQL programmer	Unblinded	Involved in extracting data from EHR and developing reports to be used for monitoring recruitment, adherence, and data	Yes	Ongoing access to extract and monitor data from EHR and/or patient surveys

		quality		

7. SAFETY ASSESSMENTS

The protocol is currently approved by the Duke IRB and Durham VA. Study activities requiring individual-level consent involve survey-based assessments only. Other research activities involve data collection from the EHR only; thus, this study has been deemed minimal risk study by the local IRB's.

7.1 Adverse Events and Serious Adverse Events

Because this study is collecting standard of care information and contact with the participants only involves telephone survey-based assessments, this is considered a minimal risk study. There is no investigational drug or device, and all data collected through EHR records are being collected through routine clinical visits as part of standard care.

A Data and Safety Monitoring Board (DSMB) will be appointed by NCCIH to review study progress, assess the adequacy of ongoing enrollment & site performance, ensure adequacy of data acquisition & protocol adherence and evaluate overall safety throughout trial implementation. The DSMB will meet at least annually after trial initiation. A DSMB Charter Document outlining the operating guidelines for the committee, the frequency of planned meetings and the specific data presentation format will be agreed upon during the initial meeting of the DSMB. Study reports will be created by the study data center, following a standardized format, as directed by the DSMB. The DSMB will report directly to NCCIH.

The PIs will report any serious, unexpected, and study-related adverse event or unanticipated problem to the local IRB and the DSMB within the time frame specified by current VHA and institution requirements. Data safety and monitoring plan has additional details.

7.2 Safety Monitoring

Because this is collecting standard of care information and contact with the participants only involves telephone survey-based assessments, this is considered a minimal risk study. There is no investigational drug or device, and all data collected through EMR records are considered standard of care data.

The DSMB will be implemented to review our research procedures and operating procedures before initiating the study and to ensure the data collection and safety-monitoring are implanted while the study is ongoing. The DSMB will also be involved with reporting of any adverse events that may occur and will make the determination of whether study stoppage is indicated. Currently we do not have any interim analyses planned for this proposal, but we will pursue this option if it is indicated by the DSMB. The DSMB will meet at least annually either in person or on teleconference and will meet more frequently if circumstances warrant. For example, in the situation when multiple adverse events may merit a reconsideration of the risk to benefit ratio for this study. The DSMB will likely consist of 3-5 individuals with the appropriate expertise to review this study, but who are not directly involved with its operation and are not considered as prior collaborators with this research team. The minutes and executive summary of each DSMB meeting will be submitted to the NCCIH, the NIH Pain Management Work Group and the local IRB's involved with this trial.

8. CLINIC DISCONTINUATION

While our goal is to not have any clinics discontinue, clinic discontinuation could occur by a clinic informing us that they were dropping out after randomization but before training and rollout of pathways or after rollout of pathways or some other unplanned circumstance. It is important to note that our design allows for additional sites to be added in the 2nd round of cluster randomization if site(s) from the first round discontinue or have lower than expected recruitment rates. This is described in more detail in Section 9 (Statistical Considerations).

One of the eligibility criteria for clinics is that the volume of back patients seen on a weekly basis is on average 8 -12 so that over a 6-month period assuming a 50% referral rate (4-6 referred per week) clinics can meet recruitment goal of 105 Veterans referred to their pathway; it may take clinics with smaller patient volumes or lower referral rates longer to enroll and weekly referrals could vary from 2 to 4 per week and we can still meet recruitment goals on our timeline.

Given that this is an embedded pragmatic trial and “enrollment” is controlled by referrals from primary care providers to the clinics randomized pathway either PNP or SCP, the study team will monitor care pathway volume monthly at each participating clinical site. The goal to monitoring pathway volume will be for the study team to trouble-shoot problems to help clinics increase pathway volume. Over the first couple of quarters of an enrollment period at a clinic we will review number of referrals with clinics to make sure meeting goals and to trouble-shoot any problems or issues. If it appears that lower than expected volume is occurring mitigating steps will include meeting with site leaders to review care pathway procedures, trouble-shoot, and enhance education outreach for referring providers. At the time that we begin enrolling block 2 clinics, we will add additional clinics if any has dropped out in block 1 and we will examine recruitment in the clinics in block 1 and determine whether we need to enroll and randomize additional clinics in block 2 to compensate for low recruitment (below target of 105 per clinic) at any of the block 1 clinics. The blocked covariate constrained randomization balances covariates within and across blocks. We will use any information from the enrollment process of the 10 clinics in block 1 to inform and improve the enrollment process for the set of clinics for block 2. We have a solid plan for dropout of a clinic or low recruitment in block 1 that maintains the randomization and balance of covariates with the ability to add clinics to block 2 before the block 2 randomization.

To guard against drop out of clinics from block 2 we plan to add 2 reserve sites to the block 2 wave of randomization to use in case a clinic should drop out of block 2. We have adjusted the block sizes to have 10 clinics randomized in block 1 and have 8 clinics in block 2 (if no clinics drop out in block 1) which include 2 reserve clinics. Through the design process for this trial we have increased the number of clinics from 12 to 16 in part to provide a safeguard for the potential drop out of a clinic and still be able to conduct a successful trial (**see Table 7** below that has effect sizes for 12 clinics with same number of patients per clinic as we targeting for 16 clinics and for 16 clinics with smaller number of patients per clinic).

Table 7. Sample Size Table for AIM-Back

Number of clinics per condition	Number of patients per clinic needed at post-intervention	Df for baseline covariate adjustment	Minimum Detectable Difference (Effect size)	Minimum Detectable Difference (Effect size)	Minimum Detectable Difference (Effect size)	Number needed at baseline with 20% attrition per clinic/

	follow-up/ Total N		ICC=0.01	ICC=0.02	ICC=0.05	Total N_b
6	42/N=504	5<br>2	0.49<br>0.44	0.56<br>0.50	0.72<br>0.65	53/ N_b=636
6	84/N=1008	5<br>2	0.40<br>0.35	0.47<br>0.42	0.66<br>0.59	105/ N_b=1260
8	32/N=512	5<br>2	0.41<br>0.40	0.46<br>0.44	0.57<br>0.55	40/ Nb=640
8	63/N=1008	5<br>2	0.32<br>0.31	0.38<br>0.37	0.52<br>0.50	79/ Nb=1264
8	42/N=672	5<br>2	0.37<br>0.36	0.42<br>0.41	0.54<br>0.53	53/ Nb=848
8	84/N=1344	5<br>2	0.30<br>0.29	0.36<br>0.35	0.50<br>0.49	105/ Nb=1680

9. STATISTICAL CONSIDERATIONS

9.1 General Design Issues

This study will be a longitudinal cluster randomized trial conducted at 16 VA clinics. The “clinics” must be geographically distinct with no overlap in staffing. A summary of study measures, with their associated definition, data source, and time interval is provided above in Section 10 (Data Collection and Measures). Outcomes derived from VA EHR data (for example utilization and pharmacy data, as well as health factor data obtained through data capture tools designed and implemented for this study) will be obtained for the larger administrative sample (n=1680). Outcomes from telephone administered surveys will be available on approximately half of participants (n=848), who are consented to participate in enhanced survey data collection. Study measures were selected to be patient-centered and consistent with recent recommendations from the NIH Task Force on Research Standards for Chronic Back Pain, and relevant to Veteran’s health and VHA.[49]

9.2 Sample Size and Randomization

In **Table 8** below we present a range of minimum detectable effect size differences for the primary outcomes of Pain Interference or Function Scores for both the administrative sample (N=84 per clinic) and survey sample (N=42 per clinic) for 16 clinics (8 clinics randomized to each arm) for Aim 1. Sample size calculations for the cluster randomized design were based on the net difference between the two conditions across baseline and 3-months follow-up.[50] We assumed over time correlations of 0.50 for both patients (based on data from the Goode et al. pilot study[33] and summary data available for Durham VA clinics, intraclass correlation coefficients (ICC) of 0.01, 0.02, and 0.05 to account for clinic clustering, and also adjusted for the potential group-level randomization of up to 4 stratification variables as baseline covariate adjustments where we conservatively assumed no reduction in either group or subject level variance components. For all calculations, the type-I error is 2.5% to account for the multiple primary outcomes and power is conservatively assumed to be 90% to guard against deviations from assumptions. The number needed at baseline (N_b) is based on an attrition rate of 20%. Note in the **below Table 4** that the scenarios above lead to having adequate power to detect a medium effect size for the primary outcomes for both the administrative and survey samples.

Table 8. Minimum detectable effect size differences for 90% power, alpha of 0.025 and ICC of 0.01, 0.02, and 0.05 for administrative outcome (n=84 per clinic) and survey outcome (n=42 per clinic)

Number of clinics per condition	Number of patients per clinic needed at post-intervention follow-up (Total N)	Minimum Detectable Effect Size Difference ICC=0.01	Minimum Detectable Effect Size Difference ICC=0.02	Minimum Detectable Effect Size Difference ICC=0.05	Number of patients per clinic needed at baseline with 20% attrition (Total N_b)
8	42 (N=672)	0.37	0.42	0.55	53 (N_b=848)
8	84 (N=1344)	0.30	0.36	0.50	105 (N_b=1680)

The standardized effect sizes[51] that we are powered to detect range from 0.30 to 0.50 for the primary outcomes on the administrative sample. This range maps to differences of approximately 2.4 to 5.0 points in the PROMIS Pain Interference score between arms assuming standard deviations in the range found in the pilot study from Goode et al.[33] These magnitude of differences have been reported to be clinically relevant.[52] Similarly for the survey sample, the range of effect size differences we can detect map to differences of approximately 3.0 to 5.5 points between arms.

For AIM 2, the subgroup analyses of the primary outcomes in the administrative sample, if we assume an even distribution of a binary moderator (e.g. opioid use), our effective sample size for detection of moderation is a quarter that for the main effect analysis yielding an effective sample size of n=336 (21 per clinic).[53] For the moderator interaction effect, we can detect medium to large effect size differences over a range of assumptions (**Table 9 below**), which allows for prudent exploration of treatment moderation.

Table 9. Minimum detectable effect size differences by a binary moderator for 90% power, alpha of 0.025 and ICC of 0.01, 0.02, and 0.05 for administrative sample.

Number of clinics per condition	Number of patients per clinic needed at post-intervention follow-up (Total N)	Minimum Detectable Effect Size Difference ICC=0.01	Minimum Detectable Effect Size Difference ICC=0.02	Minimum Detectable Effect Size Difference ICC=0.05	Number of patients per clinic needed at baseline with 20% attrition (Total N_b)
8	21 (N=336)	0.48	0.53	0.63	27 (N_b=432)

9.2.1 Treatment Assignment Procedures

In this study, randomization occurs at the clinic (i.e., the cluster) level and we plan on conducting a covariate constrained randomization[2-4] with the following clinic level covariates assessed prior to randomization: 1) average pain scale scores of LBP patients at clinic, 2) average level of opioid exposure of LBP patients at clinic, 3) number of participating primary care providers at clinic, 4) clinic location (main medical center/community clinic) and 5) average age of LBP patients at clinic. These characteristics were chosen to represent factors likely to be associated with baseline differences in patient population that may affect primary outcomes. However, the exact number of covariates we use will depend on the quality and distribution of these covariates across the 16 clinics that we enroll. For example, if all the clinics we enroll are

community clinics, then we would drop the clinic location covariate in the randomization. Currently, throughout the VA, general pain scale scores (rate pain on a scale from 1 to 10) are being collected for all primary care visits and stored in administrative data. Our plan will be to use these data, averaging the pain scale scores of LBP Veterans at each clinic over a 3 to 6-month time period prior to clinic enrollment. For Durham primary care clinics, we found that pain scores were collected at over 95% of primary care visits for lower back pain over a one-year time period. From the Goode et al. pilot study[33] of Veterans with back pain (n=50), we found that correlation of pain scale scores to PROMIS pain interference at baseline was approximately 0.55, a reasonably strong correlation. For the opioid exposure covariate, our plan is to apply the PMC3 opioid exposure definition to LBP patients at clinics over a 3 to 6-month period prior to clinic enrollment and then use the proportion of patients that meet the opioid exposure definition as the average level of opioid exposure for the clinic. The number of participating (referring) providers at a clinic will be used as an indication of size of clinic (volume of patients served). For the age of LBP patients, we will use age that is available in the electronic health record and average the age of Veterans with LBP visits to the clinic over the 3 to 6-month time period prior to clinic enrollment.

In planning for the UH3, we have determined that we cannot roll out the interventions for all 16 clinics at the same time due to logistical constraints. Therefore, we plan to conduct a covariate constrained randomization in 2 blocks following the extension to randomize multiple blocks of groups by Carter and Hood.[54] In the first block we will have 10 clinics and the second block will have 8 clinics if no clinics drop out of block 1 before randomization occurs in the second block. The second block will include 2 reserve clinics for use if a clinic should drop out from block 2 (see Section 8). In the standard covariate constrained randomization all participating clusters need to be enrolled before randomization. We are concerned that clinics that would roll out later in the schedule may lose interest or drop out if we enroll and randomize all clinics at once at the beginning of the study. An advantage of the blocked compared to the standard randomization is that, if a clinic would drop out in the first block before we randomize the second block, we would be able to add clinics in the second block to compensate for the loss. As we cannot roll out the interventions at all clinics at the same time within a block, following randomization, we will then randomly pair one SCP clinic and one PNP clinic and then randomly order pairs for intervention roll out and recruitment.

As this is a cluster randomized embedded pragmatic trial, patients, intervention delivery personnel and recruitment personnel will not be blinded to clinic intervention assignment. However, research personnel collecting study surveys by telephone will be blinded.

9.3 Definition of Populations

As this is an embedded pragmatic trial, all adult subjects that present to clinics with LBP and are referred to one of the two arms of the trial with initial contact with pain navigator or central delivery for data collection will be eligible for the primary analysis as part of the administrative sample. A subset of these Veterans will be enrolled as research subjects so that more detailed research data can be collected at regular intervals for follow up. For this subgroup, equivalent numbers of Veterans with LBP will be enrolled at each clinic.

The primary analyses will be conducted on an intention-to-treat basis; patients will be analyzed in the group to which they were randomized, regardless of intervention adherence, using all available data. The main conclusions drawn from this trial will be based on the pre-specified hypotheses outlined below and will be tested with two-sided p-values at the standard 0.05 level, except where noted due to multiple primary outcomes. For all study outcomes, we will interpret differences between groups over time with reference to prior

literature regarding clinically meaningful changes. Results from exploratory analyses will be interpreted with appropriate consideration for their exploratory nature. Statistical analyses will be performed using the latest release of SAS for Windows (Cary, NC) and R software.

9.4 Interim Analyses and Stopping Rules

Interim analyses and stopping rules are not applicable to our embedded pragmatic trial.

9.5 Outcomes

9.5.1 Primary Outcome

Co-primary outcomes will be pain interference with normal activities and functional status, assessed by PROMIS Short Form scores collected in the EHR on the administrative sample (n=1680) at baseline and 3 months; 3 months is the primary outcome time point. For baseline and 3-month follow-up, we will rely on central delivery personnel and clinical providers in SCP and pain navigators in PNP to collect these data in the EHR via progress note templates to collect health factors relevant for the analysis for the PROMIS pain interference and functions scores (see Section 6). For the follow-up, a report will be generated every 2-weeks to evaluate which participants are due for the 3-month follow-up and the report will be sent to central delivery personnel or pain navigator for them to administer these measures (see outcome ascertainment study flow diagram in Figure 4. The window around the 3-month outcome assessment will be 1-month. If outcome assessment occurs outside of this defined window they will not be included in the primary analysis. Given that this is an embedded pragmatic trial the study team will monitor outcome ascertainment timing at each participating clinical site to trouble-shoot any problems or issues to circumvent loss of outcome data.

9.5.2 Secondary Outcomes

Secondary outcomes of PROMIS sleep, opioid use, and utilization will also be obtained administratively on the full administrative sample (n=1680). As previously described for the primary measure PROMIS sleep will be collected at the same times as the primary outcomes in the EHR (see Section 6). Opioid use will be defined at baseline and then at 12 months later using the PMC3 provided definition. For imaging and ED utilization, we will measure use for the 12 months following baseline.

9.6 Data Analyses

The 3 -month follow up rate of primary outcome data collected as part of an AIM-Back clinical follow-up visit in the electronic health record has been lower than anticipated. At the recommendation of AIM-Back monitoring partners an updated statistical analysis plan was generated. The updated analysis plan allows for use of survey data to augment the follow up rate for the primary outcomes collected in the electronic health record. This updated statistical analysis plan was created and approved prior to completing enrollment and is described in more detail in the 0.51 version of the protocol. The updated analysis plan was also published here for full transparency:

https://www.medrxiv.org/content/10.1101/2023.12.22.23300382v1

Aim 1 Analyses

The primary outcomes are continuous and will be ascertained at the planned baseline and follow-up assessment (3 months) from administrative data collected from the EHR on

Veterans presenting at participating clinics that are referred and enrolled in AIM-BACK. For Veterans in the EHR sample that do not have the 3-month EHR outcome data within the 3-month follow-up window but have also participated in the survey study, we will supplement the 3-month EHR with the PROMIS measures collected from the survey when the 3- or 6-month follow-up survey is in the 2-4 month follow-up window for the EHR sample. In all cases, the 3-month EHR PROMIS measures and the survey measures are being collected by telephone. In the EHR sample, data is collected in templated notes in the EHR by clinical personnel onsite or clinical personnel from our central delivery team. Survey sample data is being collected by blinded study personnel.

Changes in pain interference and/or physical function scores will be estimated and the primary hypotheses tested via hierarchical linear mixed-effects models, with patients nested within clinics and baseline and 3 month values in the response vector.[55] Hierarchical linear models are a flexible and powerful analytic tool for clustered longitudinal continuous outcomes. The fixed-effect portion of the model will have the form: $Y_{ijk} = \beta_0 + \beta_1 * (\mathit{followup}) + \beta_2 * (\mathit{followup} * \mathit{intervention})$ for clinic *i*, patient *j,* at time *k*. Random effects (clinics and time by clinics) will be included in the model to account for clustering of patients within clinics as the clinics are the unit of randomization. Random effects will also be included to account for the within-patient correlation between repeated measures over time. We will determine the best-fitting random effects structure by fitting a variety of random coefficient models (e.g., random intercept only, random intercept and linear slope) and assessing model performance with the Akaike Information Criteria (AIC) model selection criteria.[56,57] The predictors in the model will include a time effect and indicator variables for treatment interacting with the time effect. The intraclass correlation capturing the relationship of outcomes between patients seen at the same clinic is accounted for via the random effects for the clinics and time by clinics, which are assumed to be normally distributed. The model will be fit in the SAS procedure PROC MIXED using full likelihood approximation and the hypotheses will be tested by whether the estimated coefficient β_2 is positive and significantly different than 0 at the 0.025 level due to 2 primary outcome variables. We will include covariates used in the covariate constrained randomization[4] (5 potential variables; average pain scores, clinic location (main medical center/community clinic), number of participating primary care providers, average level of opioid exposure of LBP patients at clinic, and average age of LBP patients at clinic) in our primary model as well as a limited number of patient-level covariates that are readily available in the EHR (CDC pain at referral, age, gender, race and a comorbidity measure).

The survey outcomes for the enrolled subset of patients will be collected at baseline, 3, 6, and 12 months and will include pain interference, function, intensity, catastrophizing, sleep, and depression (see Section 10 for full descriptions). These are all longitudinal continuous outcomes and a hierarchical linear model similar to that described for the primary aim will be fit. We will fit random coefficient models as described above (e.g., random intercept only, random intercept and linear slope) and assess using AIC model selection criteria to determine the best model for the covariance structure. Similarly, we will determine the best model for the mean structure (e.g., linear, quadratic, dummy coding) as there are four outcome measurement occasions guided by descriptive plots and model fit assessed using AIC model selection criteria. Due to the timing of administration of baseline surveys – some baseline surveys may occur after the initial intervention contact - we will conduct a sensitivity analysis treating baseline surveys that occur after initial provider contact as occurring in the post-treatment period.

Secondary Analyses

We will assess differences in proportions of “responders” between treatment arms for each of the primary outcomes. A “responder” will be defined as achieving 30% improvement in

pain interference or function scores at 3 months follow up.[58] Responder status for those missing outcome data will be estimated using best linear unbiased predictors from the hierarchical linear model [59]so that those missing observed follow up data will be included. We will use a generalized linear mixed model with a logit link function to compare differences in responder status between arms where the main predictor of interest will be treatment arm adjusting for clustering of VA clinic either with random effects or by conditioning.[55,60,61] As a sensitivity analysis to explore responder cut points of pain interference and function measures, we will compute cumulative proportion responder analysis graphs.[62]

Secondary outcomes of sleep, opioid use, and health utilization (including chronic opioid usage) will also be obtained administratively on all eligible subjects. The sleep PROMIS measure is a continuous outcome that will be assessed at baseline and 3 months and similar modeling procedures as described for the primary outcomes will be used. Opioid use will be examined in two ways, one as a binary variable based on whether a chronic opioid user or not at baseline and 12 months and second using a continuous measure of morphine equivalents for opioid dose at baseline and 12 months.[63] For the dichotomous outcomes, we will use a generalized linear mixed model with a logit link function where the main predictor of interest will be treatment arm and will include baseline opioid use status (chronic or not chronic) adjusting for clustering of VA clinic either with random effects or by conditioning.[55,60,61] For opioid morphine equivalent dose, we will fit a similar model as was described for the primary outcomes except the follow up time point in the model will be for 12 months. For imaging and ED utilization, we will assume a count-like distribution for the number of ED visits in the 12 months of follow up and will use generalized linear mixed model for count variables.[55]

The survey outcomes for the enrolled subset of patients will be collected at baseline, 3, 6, and 12 months and will include pain interference, function, intensity, catastrophizing, sleep, and depression (see Section 10 for full descriptions). These are all longitudinal continuous outcomes and a hierarchical linear model similar to that described for the primary aim will be fit. We will fit random coefficient models as described above (e.g. random intercept only, random intercept and linear slope) and assess using AIC model selection criteria to determine best model for the covariance structure. Similarly, we will determine the best model for the mean structure (e.g., linear, quadratic, dummy coding) as there are four outcome measurement occasions guided by descriptive plots and model fit assessed using AIC model selection criteria. Due to the timing of administration of baseline surveys that some baseline surveys may occur after the initial intervention contact, we will conduct sensitivity analysis treating baseline surveys after initial provider contact as occurring in the post-treatment period.

Sensitivity Analyses: We originally proposed a sensitivity analysis fitting models that include all EHR follow-up measurements at their follow-up time (even those outside the 3-month follow-up window) and then set up a contrast to estimate the treatment arm difference at 3-months from this model. We plan to expand on this analysis and, for the EHR sample, include all measurement time points (EHR and/or survey); the number of measurement occasions per subject will vary, ranging from a minimum of 1 (EHR baseline only) to a maximum of 8 measurement occasions and be at unequal intervals. The zero time point will be the EHR baseline, and survey time points and other EHR time points will be denoted as days from EHR baseline. As described above, we will fit random coefficient models and determine the best covariance and mean structure. A curve-fitting method (e.g., lowess, splines) will be used to estimate the average trends in outcomes and to inform the functional form of our mean trajectory model (e.g., linear or quadratic and other higher order terms). We will set up a contrast in this model to estimate the treatment

difference at 3-months, the primary time point, as well as contrasts to examine treatment differences at longer follow-up times (e.g., 6-months).

Aim 2 Analyses

As highlighted in the UG3 section we have strong clinical and scientific rationale to investigate chronic vs. acute LBP and previous opioid use vs. opioid naïve as *a priori* subgroups for treatment moderation.[38] We will define "chronic low back" as pain that has persisted for at least 3 months and resulted in pain on at least half the days in the past six months to be consistent with the NIH task force definition.[49] All participants will have chronicity of back pain assessed via the EHR template (see Section 6) at the time of initial referral to a given care pathway using questions specified in the NIH minimum dataset.[49] Opioid exposure prior to pathway entry will be defined as being prescribed at least one prescription opioid for 20 or more consecutive days (yes or no within 12 months prior to entering pathway).[64] This definition could be altered if different ones are suggested for use by the PMC3. These data will be obtained through VA's Pharmacy Benefits Management System.

For the planned subgroup analyses for key moderators on the primary outcomes of pain interference and function will be the same modeling framework as described for AIM 1 with the addition of the indicator variable(s) for the moderator and associated interactions.[65] The fixed-effect portion of the model will have the form: $Y_{ijk} = \beta_0 + \beta_1 \textit{*(moderator)} + \beta_2 \textit{*(moderator*intervention)} + \beta_3 \textit{*(followup)} + \beta_4 \textit{*(followup*intervention)} + \beta_5 \textit{*(moderator*followup)} + \beta_6 \textit{*(moderator*intervention*followup)}$ for clinic *i*, patient *j,* at time *k*. A random effects (clinics and time by clinics) will be included in the model to account for clustering of patients within clinics, as the clinics are the unit of randomization. Differential change in pain interference or function by moderator for the two intervention groups is supported if the coefficient for β_6 is significantly different than zero at the p=0.025 level. For the adherence multinomial outcome, we will fit a generalized linear model with a random effect for clinic and cumulative logit link function. As the adherence outcome is potentially a 3-level outcome there will be two interaction terms to examine for moderation effects (e.g., whether the effect of the intervention by chronicity is different for those moderately adherent vs. not adherent and/or those fully adherent vs. not adherent).

Exploratory analyses to identify multidimensional subgroups from combinations of baseline characteristics (e.g., age, sex, opioid use) for treatment moderation of the primary outcomes of pain and function as well as per protocol adherence to treatment.

Patients are likely to vary in treatment response. This variation is known as heterogeneity of treatment effects (HTE). Exploration of HTE has typically focused on one-factor-at-a-time post-hoc subgroup analyses (as described in our planned subgroup analysis); however, it is possible to instead identify multidimensional subgroups exhibiting HTE. In particular, we will apply the data-driven method GUIDE[66] (Generalized, Unbiased, Interaction Detection and Estimation) that can be used for longitudinal outcomes, so can incorporate all follow-up outcomes at 3 and 12-months, as well as multi-response outcomes. GUIDE uses a regression tree algorithm that identifies subgroups with heterogeneous effects and estimates how the treatment effect varies across the subgroups. We will include baseline demographic (e.g., age, sex, race) and clinical characteristics (e.g., LBP chronicity, opioid use, comorbid conditions) that are available from EHR. GUIDE provides bootstrap confidence intervals for the treatment effects of identified nodes and is available via a software package.

We will also examine "adherence" among all patients referred to one of the clinical pathways. We define "adherence" according to participant participation in planned sessions with

intervention team personnel (telephone or in-person) in each care pathway. The number of planned sessions will vary between and within arms (e.g., depending on response to 1st service in pain navigator arm and depending on risk stratification in integrated pathway arm); therefore, we will define adherence as follows: 1) non-adherent – attended no planned sessions 2) partially adherent – attended some, but not all planned sessions 3) attended all planned sessions. Depending on the number of planned sessions in each pathway we will explore additional multi-level variables that may be more informative; structure of the adherence variable for Aim 2 analyses will depend on data distribution.

We will describe adherence in both care pathways in order to identify patient characteristics potentially associated with program participation and improved pain outcomes. We will not test for differences in adherence rates between pathways as our goal is to gain insight into where to target clinical programs and focus efforts to improve access to and patient engagement with non-pharmacologic pain services. We will also conduct exploratory analyses on alternative definitions of adherence or aspects of intervention receipt. For example, in the pain navigator arm, differentiating based on whether someone actually received one of the recommended services.

Exploratory analyses to identify qualitative interactions that define groups of Veterans that have greater improvement in pain and function with the Sequenced Care pathway versus the care management program, or greater improvement with care management versus Sequenced Care, or no difference in response.

As part of personalized medicine, tailored treatment recommendations are the goal. In this trial, we are offering two different treatments, and we want to explore identifying qualitative interactions that define groups of individuals that have greater improvement with one treatment over the other. We will apply the tree-based method called Qualitative Interaction Trees (QUINT) to identify these subgroups.[67,68] QUINT is a tree-based clustering method for data obtained from a two-armed trial that identifies three subgroups defined by different baseline characteristics of subjects in the study. QUINT uses a partitioning criterion that is maximized using a sequential partitioning algorithm or a stepwise binary splitting procedure. We will use the R package *quint* and apply to our study data to identify characteristics for Veterans where Sequenced Care pathway treatment results in greater improvement in outcomes than care management pathway, subgroup of Veterans that care management pathway results in greater improvements than Sequenced Care pathway and the subgroup for which improvement does not differ between treatments. We will first construct a summary of each patient's repeated measures profile to characterize the patient's change over time at the primary time point 3-months (i.e., whether or not the patient improved and mean improvement). Empirical Bayes estimates from hierarchical linear models will be used to generate individual-level estimates of mean improvement for each of the primary outcomes. Currently this software only supports continuous outcomes so we would not be able to apply to the multilevel response adherence outcome; however, we will monitor updates for expansion to other distributions of outcomes. Limitation of this methods are that subjects with missing data on any included variables are omitted from the analysis, the effect of clustering within clinics is not addressed, and categorical baseline characteristics of more than two categories cannot be used.

Statistical methods for heterogeneity of treatment effects (HTE) are a rapidly developing field. We will monitor the development of new methods that may be applicable to our study for adapting the analysis plan for this aim and work closely with the PMC3 Biostatistics Working Group and Dr. Lisa Wruck (an internal advisory member for this proposal) to ensure robustness of our chosen methods.

Missing Data. We do not anticipate much missing data in the main predictors of interest, intervention arm, or patient characteristics available in the EHR or assessed at baseline in survey outcomes. There may be missing values in the outcome measures due to dropout, death, a missed interim assessment, or an assessment outside the 1-month window for 3-month follow-up, or item non-response. However, hierarchical linear mixed models via maximum likelihood estimation, our main analysis technique for the primary outcomes, implicitly accommodate missingness at random (MAR).[55] Therefore, inferences will be valid even with differential dropout by intervention arm. We will thoroughly explore reasons for dropout and, depending upon the type and scope of missing data, we may explore the sensitivity of intervention effects to different missing data mechanisms (MAR vs. MNAR).[69] Our plan would be to conduct sensitivity analyses to evaluate the sensitivity of the assumption of MAR and/or MNAR on intervention effects. If our primary model included design variables only (treatment, stratification variables, clinic), this model would meet the MAR assumptions if missing data is related to previous outcome assessments or design variables included in the primary model. We can assess the sensitivity of the MAR assumption by conducting an analysis that includes auxiliary variables (either as additional variables in primary models or by conducting a multiple imputation including auxiliary variables). These auxiliary variables or other baseline characteristics for the large sample may be limited due to availability in the EHR and will explore additional variables including baseline opioid exposure. We are currently proposing to include age, sex, race, CDC chronic pain and a comorbidity index in our primary analysis that may strengthen our MAR assumption. To explore the MAR assumption, outcomes will be multiply imputed using principled methods in SAS (via PROC MI or IVEware).[70] Multiple imputation provides a framework for being able to incorporate information from important auxiliary variables while still preserving a parsimonious main treatment effect model and is described as a significant advantage in recommendations from Panel on Handling Missing Data in Clinical Trials.[71] Note that if needed, we will utilize imputation methods that account for the multiple levels of correlation inherent in the clustered data structure. If we cannot justify the assumption of MAR, we will explore the sensitivity of intervention effects to the MNAR assumption; we will follow guidelines in Mallinckrodt[72] and Ratitch et al[69] for model selection and pattern mixture modeling.

In contrast to an EHR study where all measurements are determined by clinical practice and do not follow a data collection schedule, the AIM-BACK program has a data collection schedule that has been implemented in the clinical process with outcome assessments documented in the EHR. Thus, standard missing data techniques for missing outcome measurements can be applied for those enrolled, as described above. However, because Veterans are referred to the AIM-BACK program by providers and must attend the first AIM-BACK visit to be enrolled, the potential for selection bias exists with a significant number of Veterans referred that are not enrolled and have no outcome data. Through April 2023, we have approximately 2000 referrals with over 1400 Veterans enrolled in the program; approximately 70% of referrals led to enrollment in the AIM-BACK program with around 30% of referrals discontinued or cancelled across clinics. In monthly reports during the course of our study, we have been examining differences in CDC pain, age, gender, race, and ethnicity of Veterans referred and enrolled in the program to Veterans referred that do not enroll in the program. Overall to date, the patient demographics and CDC pain rates are similar between those enrolled and not enrolled. However, as we have no outcome data on those that do not enroll, in sensitivity analyses we will use inverse probability weighting methods (IPW) to adjust for this selection bias. [Peskoe SB, Arterburn D, Coleman KJ, Herrinton LJ, Daniels MJ, Haneuse S. Adjusting for

selection bias due to missing data in electronic health records-based research. Stat Methods Med Res. 2021 Oct;30(10):2221-2238. doi: 10.1177/09622802211027601. Epub 2021 Aug 26. PMID: 34445911.] Probability weights for the probability of enrolling in the AIM-BACK program will be generated by fitting a logistic regression model to the binary enrollment indicator, with covariates for patient EHR demographics, and CDC pain at time of referral; we will explore whether adjustment for clinic is needed either as a fixed or random effect. We will adjust our primary model described above with probability weights and estimate treatment effects at 3-months.

10. MEASURES AND DATA COLLECTION

Outcome measures and timing of collection is summarized in **Table 10**. The data collection process is explained in subsequent sections. In version 0.51 of the protocol removed the 9 month follow up collection as that decision had been made early in trial to limit administrative burden of survey follow ups.

Table 10. Summary of Measures

Outcome Variable	Definition	Data Source	Baseline	3 months	6 months	12 months
**EHR Outcomes**						
Pain Interference	PROMIS SF- 4 item	CDW health factor data element	x	x		
Physical function	PROMIS SF- 4 item	CDW health factor data element	x	x		
Pain intensity	Numeric Scale (0-10)	CDW health factor data element	x	x		
Opioid Chronic Use	At least one prescription opioid for 120 days or more (non-consecutive) or 10 refills	Pharmacy Benefits Management	x			x
Opioid Exposure	At least one prescription	Pharmacy Benefits Management	x			x
Opioid Morphine Equivalent (MEDD)	Total mg MEDD supplied in time frame	Pharmacy Benefits Management	x			x
Adverse clinical outcomes	(1) accidents resulting in wounds or injuries, (2) opioid-related accidents and overdose, (3) alcohol- and nonopioid drug–related accidents and overdose, (4) self-inflicted injuries and (5) violence-related injuries	CDW inpatient & outpatient<br>Non-VA care (Fee) inpatient & outpatient (ICD codes)				x
Sleep disturbance	PROMIS SF- 4 item	CDW health factor data element	x	x		
Sedative/hypnotic exposure	At least one prescription sedative/hypnotic for 20 or more consecutive days	Pharmacy Benefits Management	x			x
PTSD symptoms	PCL-5	CDW health factor data element	x			x
Imaging, Providers, and Procedures	Utilization<br>- Radiographs, MRI, CT<br>- Provider types and number	CDW inpatient & outpatient<br>Non-VA care (Fee)<br>CPT outpatient + ICD codes				x

	- Injection, ablation, and surgery					
ED visits	ED visits<br>- VA<br>- Non-VA	CDW inpatient & outpatient<br>Non-VA care (Fee) inpatient & outpatient				x
Pain-related ED visits	ED visits<br>- VA<br>- Non-VA	CDW inpatient & outpatient files;<br>Non-VA care (Fee) inpatient & outpatient + ICD codes				x
**Survey (CATI) outcomes**						
Pain Interference	PROMIS SF- 4 items	Patient report on CATI survey	x	x	x	x
Physical Function	PROMIS SF-4 items	Patient report on CATI survey	x	x	x	x
Pain intensity	PEG Pain Screening Tool	Patient report on CATI survey	x	x	x	x
Catastrophizing	NIH recommended minimum dataset (2 items)	Patient report on CATI survey	x	x	x	x
Self-Efficacy	Patient Self Efficacy Questionnaire (2 items)	Patient report on CATI survey	x	x	x	x
Quality of life	Euroquol - 5 items	Patient report on CATI survey	x	x	x	x
Sleep disturbance	PROMIS SF - 4 item	Patient report on CATI survey	x	x	x	x
Depressed Mood	PHQ-2 Questionnaire	Patient report on CATI survey	x	x	x	x
Alcohol	AUDIT-C	Patient report on CATI survey	x	x	x	x

10.1 Survey Data Collection Process

Because the participation in the two care pathways described earlier in the protocol is considered a quality improvement initiative, local site activities and participation in the two care pathways will not require IRB oversight. Two separate (Durham VA and Duke University) IRBs have been approved for described research related project activities. The Durham VAMC IRB will be the IRB of record to cover participant survey recruitment activities, including obtaining verbal informed consent for participating in the telephone survey, administering a telephone survey for baseline and follow-up data collection activities, medical record chart abstraction, and post data collection analysis activities.

The table above documents the potential questions which will be asked in baseline and follow up interviews. These questions will serve as the CRFs for the survey data collection activities.

Pre-consent screening data will be collected in the VA instance of REDCap survey software. Following verbal consent, baseline assessments will be collected by VA staff using the VA instance of REDCap. Post-consent, follow-up assessments will also be collected by VA staff using the VA instance of REDCap. The recruitment and participant management system will be a Durham VA in-house application named Study Tracking that will be run by the VA staff. It will provide all functionality needed to keep track of study participants as they move through the protocol. Study Tracking will interface with the REDCap survey software.

10.2 Data Management

The Durham VA will serve as the coordinating center and data management center for this study. The Durham VA will be responsible for enrollment, consenting participants for the follow up telephone survey assessments, baseline and follow-up data collection, as well as data analysis after the database lock. Other than standard demographic information, all of the outcome measures being collected for this study via follow up surveys are harmonious with other existing patient reported pain and pain impact measures collected within the field.

The follow up surveys will be administered at three, six, and twelve months post baseline, and participants will be paid $25 for completing each survey, including baseline. Payments will be made via direct deposit or mailed to the participants' address. Payments will be administered through the Durham VA.

10.3 Quality Assurance

All VA employees will receive the appropriate training to use the various data collection and participant tracking systems.

All new interviewers complete training that covers topics on Human Subject Protection (HSP), HIPAA Privacy and Security, standardized interviewing procedures, and instrument specific training. Interviewers follow the questionnaire scripts which are displayed directly on their computer screen as they precede through the interview.

The standard interviewing training module teaches and guides interviewers to remain neutral, to read questions clearly and slowly, and to avoid rewording, asking supplemental questions, making assumptions, or acting surprised/interested to a subject's responses. Until training is fully complete, interviewers complete any questionnaires under supervision.

In addition to the thorough training, project staff monitor and perform quality assurance checks on interviewers on an ongoing basis. Using a checklist, staff monitor and provide feedback to interviewers about any issues during an interview. Study staff also review data from completed interviews and data discrepancy checks to ensure questionnaires are completed thoroughly, no questions are being systematically skipped and that valid responses are being captured and to notice any trends in data quality that may indicate any biases from the way surveys are administered. We will not be looking for trends in the data over time, but instead determining if we can improve on any missing data trends, or invalid response patterns noticed during data quality checks. If any issues arise during the monitoring or during any quality assurance checks, interviewers are provided with a re-training and receive continual monitoring.

10.3.2 Quality Control Committee

Not applicable for this embedded pragmatic trial.

10.3.3 Metrics

The REDCap and Study Tracking databases will be used to track rates of follow up completion in real time. If at any time follow up completion rates are concerning, study staff members will evaluate processes and brainstorm better ways to capture follow up data. Based on past experiences, we anticipate between a 75-90% success rate for collecting follow up data. In order to achieve a high success rate, the interviewing staff will continue to contact participants as many times as possible during the eligibility window (15 days prior to an interval date, and 15 days post an interval date).

10.3.4 Protocol Deviations

Because the participation in and data collected from the two care pathways described earlier in the protocol is considered a quality improvement initiative, there will be no increased risk from standard of care for participants during these project activities. For the sub-group of participants who will be consented to be contacted for the follow-up telephone survey-based assessments, we expect these activities to be considered minimal risk from the Durham VA and Duke University Health System IRBs. The loss of confidentiality will be the greatest risk for participating in the telephone survey-based assessments. There is no investigational drug or device, and all data collected through EMR records are considered standard of care data. One reportable protocol deviation we anticipate would be enrolling participants who do not meet our established eligibility criteria outlined in section four.

10.3.5 Monitoring

Site monitoring (e.g., recruitment and adherence) described earlier (Section 8).

11. PARTICIPANT RIGHTS AND CONFIDENTIALITY

All baseline and follow up research material will be obtained from participants during phone calls with Durham VA study staff or from the medical files of participating Veterans by Durham VA research staff. Data obtained from medical files will be drawn from existing records generated in

the course of clinical care. The following human subjects related data elements will be collected for this study, with the source(s) of information noted:

- Presence of inclusion and exclusionary health conditions and clinical criteria (VA electronic medical records and self-report)
- Subject demographic and clinical characteristics, including age, gender, race / ethnicity, income, education level, and marital status (VA electronic medical records and subject self-report)
- Subject-reported clinical and functional status, health information quality of life, pain impact measures, information on co-morbidities (scales administered by research staff)
- Completion of telephone calls as part of the study
- Participant health care use during the study period (VA electronic medical records)

Only individuals officially assigned to the study team will have access to individually identifiable information about human subjects. This will include the principal investigators, co-investigators, statisticians, computer programmer, project coordinator, and research assistants. All of these individuals will have completed required human subjects training and will be included on a staff listing with the IRB.

Potential Risks. There are few potential risks associated with this study. It is possible that some participants may feel uncomfortable answering some of these questions. We will only ask questions that involve data that are important for study outcomes, and we will inform participants that they may refuse to answer any questions, but still be involved in the study. Since personal data will be collected as part of this study, there is a risk of loss of confidentiality. However, we will take several measures to minimize this risk. First, we will only collect the data necessary for the study. Second, all electronic data will be stored on secure servers, rather than on individual desktop or laptop computers. Third, electronic study data will be kept in folders and databases that are only accessible to key personnel who are IRB-certified and whose job functions require access to these data.

Adequacy of Protection from Risks; including recruitment and informed consent and protections against risks including data security and sharing

Protection against risk. We do not anticipate any significant physical, psychological, social, financial, or legal risks to be associated with participation in this study. In order to minimize any risks regarding privacy of individuals and confidentiality of data, we will take specific measures to protect both paper and electronic data. Except when required by law, participants will not be identified by name, social security number, address, telephone number, or any other direct personal identifier in study records disclosed. All electronic data will be stored on secure servers in folders and databases accessible only to study personnel whose job functions require access to this information. We will minimize the use of paper data collection by entering information from telephone screening interviews, baseline and follow-up assessments, and intervention tracking directly into a computer database. Any paper-based documents will be stored in a locked filing cabinet in a locked office. We have used these data security procedures successfully in multiple prior studies. We will follow standard procedures for reporting any adverse events and other unanticipated problems to the IRB. Scopes of practice for all research staff members will be approved by and maintained on record with the Durham and Duke IRBs.

11.1 Institutional Review Board (IRB) Review

IRB protocol review approved at Duke University and Durham VA.

11.2 Informed Consent Forms

A script for the approved informed consent documents for the Durham VA IRB has been drafted. Trained research staff will obtain informed consent according to existing VA policies and procedures using an IRB approved protocol. Because the study is conducted entirely over the phone, we were granted a waiver of documentation of informed consent and HIPAA authorization. We will maintain study files with documentation of the conversation in which verbal consents and authorizations were obtained, for inspection by regulatory bodies as appropriate.

After obtaining verbal informed consent, the VA RA will collect baseline survey data from all study participants. A well established and IRB-approved protocol will be used for Veteran who express suicide ideation during baseline or at any of the follow-up phone calls. The study will be conducted according to Good Clinical Practice guidelines, the U.S. Code of Federal Regulations (CFR) Title 21 CFR (Part 50 – Protection of Human Subjects and Part 56 – Institutional Review Boards) and the Declaration of Helsinki.

11.3 Participant Confidentiality

The Durham HSR&D COIN adheres to VA policy and Durham VAMC IRB requirements, and has also developed additional Standard Operating Procedures for data security which have been designed to ensure continued confidentiality, integrity, and availability of research data. These procedures, which protect both paper and computer-based records, have been used successfully in many studies, and will be followed for the proposed study.

With respect to all data, these procedures mandate the following to ensure confidentiality and safe handling of all data:
1. Access to all participant data and information will be restricted to authorized personnel.
2. Participants will not be identified by name in any reports or publications, nor will data be presented in such a way that the identity of individual participants can be inferred.
3. Each participant will be assigned an anonymous study ID which will be used on all study forms.
4. All study personnel will maintain certification with the Durham VAMC IRB that they have completed training in research ethics and confidentiality.

With respect to paper-based records, these procedures mandate the following:
1. All study records that contain participant information will be kept in secured, locked areas when not in use.
2. In addition, such materials, when in use, will be kept safe from public scrutiny.

With respect to computer-based records, the following practices are followed:
1. All research data are stored on approved servers which are in a physically secured server room.
2. Individual computer accounts, password protected, are issued to staff members.
3. Access to computer data is granted by OI&T personnel after confirming appropriate documentation through the IRB, per COIN policies.

Utilization data, in particular, will be downloaded directly from national files to the Durham HSR&D COIN servers. Of study personnel, only the Statisticians will have access to these data, which will not be moved from this secured environment.

All patient information collected in the context of this research study, and even the fact that an individual is participating in the study, will be considered confidential. This confidentiality will be assured through several mechanisms. First, each participant will be assigned an anonymous

study ID which will be used on all study forms. Second, all study forms and paper records that contain participant information will be kept in secured, locked areas when not in use. In addition, such materials, when in use, will be kept away from public scrutiny. Materials that need to be discarded will be destroyed. Third, access to all participant data and information will be restricted to authorized personnel. In the case of computerized study data, access to data will be password protected and staff members will be assigned individualized passwords that allow them access to only those elements of the data management system to which they are authorized. In addition, all study personnel will maintain certification with the Durham VA IRB that they have completed training in research ethics, which includes training on confidentiality. Finally, participants will not be identified by name in any reports or publications, nor will data be presented in such a way that the identity of individual participants can be inferred.

11.4 Study Discontinuation

This study may be discontinued at any time by the DSMB, IRB, the NCCIH, the OHRP, the FDA, or other government agencies as part of their duties to ensure that research participants are protected. The NCCIH must be notified of any IRB or agency discussions that may result temporary or permanent study discontinuation. There is more detailed information on study discontinuation in the DSMP.

12. COMMITTEES

Study PIs participate in the monthly PMC Steering Committee meeting and we have an active representative on all PMC workgroups, which also meets monthly. Finally, we have study specific meetings once per month for the entire team (i.e. “Large Group Meeting”, monthly cadence), and we have task specific meetings as needed for work directed at milestones and deadlines (i.e. “Task Group Meetings”, weekly cadence). The NIH representative is included in all invitations for the Large Group Meetings and can join via teleconference.

13. PUBLICATION OF RESEARCH FINDINGS

One advantage of this embedded pragmatic trial is that the care strategies were meant to be sustainable which will improve translation into routine clinical practice outside of the sites that participated in the trial, if results warrant such translation. The results of this study will be relevant to broad audiences thus we plan to disseminate study results in both academic and non-academic forums. Our dissemination efforts will be assisted by the Durham HSR&D COIN Stakeholder Engagement Core, a group formed to help investigators identify important stakeholders with whom to share research results, including government/policy makers, patients, Veterans, the public, researchers, advocacy groups, and the press. Examples of how dissemination and implementation of trial findings will proceed:

- Preliminary findings and results will be presented to the NIH Pain Management Collaboratory Work Group This input will help to guide future implementation strategies within the VA Health System and beyond
- Prior to dissemination in publication or presentations, Veteran and provider stakeholders will be presented with the data and asked to provide input regarding their own interpretations of research findings, particularly the relevance to the stakeholder category to which they belong.
- Provider stakeholders involved with this proposal will facilitate dissemination by presenting key findings at meetings and conferences.
- Scientific dissemination will be aimed at those most likely to have a vested interest in implementing these trial findings.

- At conclusion of study Duke, VA, and clinicaltrials.gov websites will host summary data, downloadable versions of key papers, and the manual of procedures and operations for each care pathway.

14. REFERENCES

1. Kerns RD, Philip EJ, Lee AW, Rosenberger PH. Implementation of the veterans health administration national pain management strategy. *Translational behavioral medicine.* 2011;1(4):635-643.
2. Turner EL, Li F, Gallis JA, Prague M, Murray DM. Review of Recent Methodological Developments in Group-Randomized Trials: Part 1-Design. *American journal of public health.* 2017;107(6):907-915.
3. Raab GM, Butcher I. Balance in cluster randomized trials. *Statistics in medicine.* 2001;20(3):351-365.
4. Li F, Turner EL, Heagerty PJ, Murray DM, Vollmer WM, DeLong ER. An evaluation of constrained randomization for the design and analysis of group-randomized trials with binary outcomes. *Statistics in medicine.* 2017;36(24):3791-3806.
5. Medicine Io. Relieving Pain in America: A Blueprint for Transforming Prevention, Care, Education, and Research In. Washington, DC: The National Academies Press; 2011.
6. Committee IPRC. National Pain Strategy: A Comprehensive Population Health Level Strategy for Pain. In: Services HaH, ed. Washington, DC National Institutes of Health; 2015.
7. Lentz TA, Harman JS, Marlow NM, George SZ. Application of a Value Model for the Prevention and Management of Chronic Musculoskeletal Pain by Physical Therapists. *Physical therapy.* 2017.
8. Nahin RL. Severe Pain in Veterans: The Effect of Age and Sex, and Comparisons With the General Population. *The journal of pain : official journal of the American Pain Society.* 2017;18(3):247-254.
9. Butchart A, Kerr EA, Heisler M, Piette JD, Krein SL. Experience and management of chronic pain among patients with other complex chronic conditions. *The Clinical journal of pain.* 2009;25(4):293-298.
10. Sinnott P, Wagner TH. Low back pain in VA users. *Archives of internal medicine.* 2009;169(14):1338-1339; author reply 1339.
11. Dowell D, Haegerich TM, Chou R. CDC Guideline for Prescribing Opioids for Chronic Pain--United States, 2016. *Jama.* 2016;315(15):1624-1645.
12. Chou R, Deyo R, Friedly J, et al. Nonpharmacologic Therapies for Low Back Pain: A Systematic Review for an American College of Physicians Clinical Practice Guideline. *Annals of internal medicine.* 2017.
13. Chou R, Deyo R, Friedly J, et al. AHRQ Comparative Effectiveness Reviews. In: *Noninvasive Treatments for Low Back Pain.* Rockville (MD): Agency for Healthcare Research and Quality (US); 2016.
14. Hoffmann TC, Glasziou PP, Boutron I, et al. Better reporting of interventions: template for intervention description and replication (TIDieR) checklist and guide. *BMJ (Clinical research ed).* 2014;348:g1687.
15. Johnson MI, Paley CA, Howe TE, Sluka KA. Transcutaneous electrical nerve stimulation for acute pain. *The Cochrane database of systematic reviews.* 2015(6):Cd006142.
16. Johnson MI, Walsh DM. Pain: continued uncertainty of TENS' effectiveness for pain relief. *Nature reviews Rheumatology.* 2010;6(6):314-316.

17. **Khadilkar A, Odebiyi DO, Brosseau L, Wells GA. Transcutaneous electrical nerve stimulation (TENS) versus placebo for chronic low-back pain. *The Cochrane database of systematic reviews.* 2008(4):Cd003008.**

18. **Sluka KA, Bjordal JM, Marchand S, Rakel BA. What makes transcutaneous electrical nerve stimulation work? Making sense of the mixed results in the clinical literature. *Physical therapy.* 2013;93(10):1397-1402.**

19. **Ainsworth L, Budelier K, Clinesmith M, et al. Transcutaneous electrical nerve stimulation (TENS) reduces chronic hyperalgesia induced by muscle inflammation. *Pain.* 2006;120(1-2):182-187.**

20. **DeSantana JM, Da Silva LF, De Resende MA, Sluka KA. Transcutaneous electrical nerve stimulation at both high and low frequencies activates ventrolateral periaqueductal grey to decrease mechanical hyperalgesia in arthritic rats. *Neuroscience.* 2009;163(4):1233-1241.**

21. **Kalra A, Urban MO, Sluka KA. Blockade of opioid receptors in rostral ventral medulla prevents antihyperalgesia produced by transcutaneous electrical nerve stimulation (TENS). *The Journal of pharmacology and experimental therapeutics.* 2001;298(1):257-263.**

22. **Sluka KA, Deacon M, Stibal A, Strissel S, Terpstra A. Spinal blockade of opioid receptors prevents the analgesia produced by TENS in arthritic rats. *The Journal of pharmacology and experimental therapeutics.* 1999;289(2):840-846.**

23. **Goncalves TC, Londe AK, Albano RI, et al. Cannabidiol and endogenous opioid peptide-mediated mechanisms modulate antinociception induced by transcutaneous electrostimulation of the peripheral nervous system. *Journal of the neurological sciences.* 2014;347(1-2):82-89.**

24. **Al-Hasani R, Bruchas MR. Molecular mechanisms of opioid receptor-dependent signaling and behavior. *Anesthesiology.* 2011;115(6):1363-1381.**

25. **Beneciuk JM, Bishop MD, Fritz JM, et al. The STarT back screening tool and individual psychological measures: evaluation of prognostic capabilities for low back pain clinical outcomes in outpatient physical therapy settings. *Physical therapy.* 2013;93(3):321-333.**

26. **Moran F, Leonard T, Hawthorne S, et al. Hypoalgesia in response to transcutaneous electrical nerve stimulation (TENS) depends on stimulation intensity. *The journal of pain : official journal of the American Pain Society.* 2011;12(8):929-935.**

27. **Simon CB, Riley JL, 3rd, Fillingim RB, Bishop MD, George SZ. Age Group Comparisons of TENS Response Among Individuals With Chronic Axial Low Back Pain. *The journal of pain : official journal of the American Pain Society.* 2015;16(12):1268-1279.**

28. **Choi BK, Verbeek JH, Tam WW, Jiang JY. Exercises for prevention of recurrences of low-back pain. *The Cochrane database of systematic reviews.* 2010(1):Cd006555.**

29. **Hayden JA, van Tulder MW, Tomlinson G. Systematic review: strategies for using exercise therapy to improve outcomes in chronic low back pain. *Annals of internal medicine.* 2005;142(9):776-785.**

30. Allen KD, Oddone EZ, Coffman CJ, et al. Telephone-based self-management of osteoarthritis: A randomized trial. *Annals of internal medicine.* 2010;153(9):570-579.
31. Allen KD, Oddone EZ, Coffman CJ, et al. Patient, Provider, and Combined Interventions for Managing Osteoarthritis in Primary Care: A Cluster Randomized Trial. *Annals of internal medicine.* 2017.
32. Allen KD, Yancy WS, Jr., Bosworth HB, et al. A Combined Patient and Provider Intervention for Management of Osteoarthritis in Veterans: A Randomized Clinical Trial. *Annals of internal medicine.* 2016;164(2):73-83.
33. Goode AP, Taylor SS, Hastings SN, Stanwyck C, Coffman CJ, Allen KD. Effects of a Home-Based Telephone-Supported Physical Activity Program for Older Adult Veterans With Chronic Low Back Pain. *Physical therapy.* 2018;98(5):369-380.
34. Hill JC, Dunn KM, Lewis M, et al. A primary care back pain screening tool: identifying patient subgroups for initial treatment. *Arthritis and rheumatism.* 2008;59(5):632-641.
35. Wideman TH, Hill JC, Main CJ, Lewis M, Sullivan MJ, Hay EM. Comparing the responsiveness of a brief, multidimensional risk screening tool for back pain to its unidimensional reference standards: the whole is greater than the sum of its parts. *Pain.* 2012;153(11):2182-2191.
36. Beneciuk JM, Fritz JM, George SZ. The STarT Back Screening Tool for prediction of 6-month clinical outcomes: relevance of change patterns in outpatient physical therapy settings. *The Journal of orthopaedic and sports physical therapy.* 2014;44(9):656-664.
37. Chan AW, Tetzlaff JM, Altman DG, et al. SPIRIT 2013 statement: defining standard protocol items for clinical trials. *Annals of internal medicine.* 2013;158(3):200-207.
38. Gurung T, Ellard DR, Mistry D, Patel S, Underwood M. Identifying potential moderators for response to treatment in low back pain: A systematic review. *Physiotherapy.* 2015;101(3):243-251.
39. Patel S, Hee SW, Mistry D, et al. Programme Grants for Applied Research. In: *Identifying back pain subgroups: developing and applying approaches using individual patient data collected within clinical trials.* Southampton (UK): NIHR Journals Library

Copyright (c) Queen's Printer and Controller of HMSO 2016. This work was produced by Patel et al. under the terms of a commissioning contract issued by the Secretary of State for Health. This issue may be freely reproduced for the purposes of private research and study and extracts (or indeed, the full report) may be included in professional journals provided that suitable acknowledgement is made and the reproduction is not associated with any form of advertising. Applications for commercial reproduction should be addressed to: NIHR Journals Library, National Institute for Health Research, Evaluation, Trials and Studies Coordinating Centre, Alpha House, University of Southampton Science Park, Southampton SO16 7NS, UK.; 2016.

40. **Beneciuk JM, Hill JC, Campbell P, et al. Identifying Treatment Effect Modifiers in the STarT Back Trial: A Secondary Analysis. *The journal of pain : official journal of the American Pain Society.* 2017;18(1):54-65.**

41. **Steenstra IA, Knol DL, Bongers PM, Anema JR, van Mechelen W, de Vet HC. What works best for whom? An exploratory, subgroup analysis in a randomized, controlled trial on the effectiveness of a workplace intervention in low back pain patients on return to work. *Spine.* 2009;34(12):1243-1249.**

42. **Damschroder LJ, Aron DC, Keith RE, Kirsh SR, Alexander JA, Lowery JC. Fostering implementation of health services research findings into practice: a consolidated framework for advancing implementation science. *Implementation science : IS.* 2009;4:50.**

43. **Childs JD, Fritz JM, Wu SS, et al. Implications of early and guideline adherent physical therapy for low back pain on utilization and costs. *BMC health services research.* 2015;15:150.**

44. **Fritz JM, Brennan GP, Hunter SJ. Physical Therapy or Advanced Imaging as First Management Strategy Following a New Consultation for Low Back Pain in Primary Care: Associations with Future Health Care Utilization and Charges. *Health services research.* 2015;50(6):1927-1940.**

45. **Fritz JM, Cleland JA, Brennan GP. Does adherence to the guideline recommendation for active treatments improve the quality of care for patients with acute low back pain delivered by physical therapists? *Medical care.* 2007;45(10):973-980.**

46. **Goode AP, Richardson WJ, Schectman RM, Carey TS. Complications, revision fusions, readmissions, and utilization over a 1-year period after bone morphogenetic protein use during primary cervical spine fusions. *The spine journal : official journal of the North American Spine Society.* 2014;14(9):2051-2059.**

47. **Friedman BW, Chilstrom M, Bijur PE, Gallagher EJ. Diagnostic testing and treatment of low back pain in US emergency departments. A national perspective. *Spine.* 2010;35(24):E1406-E1411.**

48. **Hastings SN, Smith VA, Weinberger M, Oddone EZ, Olsen MK, Schmader KE. Health services use of older veterans treated and released from veterans affairs medical center emergency departments. *Journal of the American Geriatrics Society.* 2013;61(9):1515-1521.**

49. **Deyo RA, Dworkin SF, Amtmann D, et al. Report of the NIH Task Force on research standards for chronic low back pain. *J Pain.* 2014;15(6):569-585.**

50. **Murray DD. *Design and Analysis of Group Randomized Trials.* New York, New York.: Oxford University Press; 1998.**

51. **Cohen J. *Statistical power analysis for the behavioral sciences.* Hillsdale, NJ: Lawrence Earlbaum Associates; 1988.**

52. **Amtmann D, Kim J, Chung H, Askew RL, Park R, Cook KF. Minimally important differences for Patient Reported Outcomes Measurement Information System pain interference for individuals with back pain. *Journal of Pain Research.* 2016;9:251-255.**

53. Leon AC, Heo M. Sample Sizes Required to Detect Interactions between Two Binary Fixed-Effects in a Mixed-Effects Linear Regression Model. *Computational statistics & data analysis.* 2009;53(3):603-608.
54. Carter BR, Hood K. Balance algorithm for cluster randomized trials. *BMC medical research methodology.* 2008;8:65.
55. Gibbons Ha. *Longitudinal Data Analysis.* Hoboken, NJ: Wiley & sons; 2006.
56. Akaike H. Information Theory and an Extension of the Maximum Likelihood Principle. In: Parzen E, Tanabe K, Kitagawa G, eds. *Selected Papers of Hirotugu Akaike.* New York, NY: Springer New York; 1998:199-213.
57. Burnham KP, Anderson DR. *Model Selection and Multimodel Inference.* 2 ed: Springer-Verlag New York; 2002.
58. Dworkin RH, Turk DC, Wyrwich KW, et al. Interpreting the clinical importance of treatment outcomes in chronic pain clinical trials: IMMPACT recommendations. *The journal of pain : official journal of the American Pain Society.* 2008;9(2):105-121.
59. Ramon C. Littell PD, George A. Milliken PD, Walter W. Stroup PD, Russell D. Wolfinger PD, Oliver Schabenberger PD. *SAS® for Mixed Models.* 2 ed: SAS Institute; 2006.
60. Kahan BC. Accounting for centre-effects in multicentre trials with a binary outcome - when, why, and how? *BMC medical research methodology.* 2014;14:20.
61. Localio AR, Berlin JA, Ten Have TR, Kimmel SE. Adjustments for center in multicenter studies: an overview. *Annals of internal medicine.* 2001;135(2):112-123.
62. Farrar JT, Dworkin RH, Max MB. Use of the cumulative proportion of responders analysis graph to present pain data over a range of cut-off points: making clinical trial data more understandable. *Journal of pain and symptom management.* 2006;31(4):369-377.
63. Von Korff M, Saunders K, Thomas Ray G, et al. De facto long-term opioid therapy for noncancer pain. *The Clinical journal of pain.* 2008;24(6):521-527.
64. Seal KH, Shi Y, Cohen G, et al. Association of mental health disorders with prescription opioids and high-risk opioid use in US veterans of Iraq and Afghanistan. *Jama.* 2012;307(9):940-947.
65. Kent DM, Rothwell PM, Ioannidis JP, Altman DG, Hayward RA. Assessing and reporting heterogeneity in treatment effects in clinical trials: a proposal. *Trials.* 2010;11:85.
66. Loh WY, Fu H, Man M, Champion V, Yu M. Identification of subgroups with differential treatment effects for longitudinal and multiresponse variables. *Statistics in medicine.* 2016;35(26):4837-4855.
67. Dusseldorp E, Doove L, Mechelen I. Quint: An R package for the identification of subgroups of clients who differ in which treatment alternative is best for them. *Behavior research methods.* 2016;48(2):650-663.
68. Dusseldorp E, Van Mechelen I. Qualitative interaction trees: a tool to identify qualitative treatment-subgroup interactions. *Statistics in medicine.* 2014;33(2):219-237.

69. Ratitch B, O'Kelly M, Tosiello R. Missing data in clinical trials: from clinical assumptions to statistical analysis using pattern mixture models. *Pharmaceutical statistics.* 2013;12(6):337-347.

70. Collins LM, Schafer JL, Kam CM. A comparison of inclusive and restrictive strategies in modern missing data procedures. *Psychological methods.* 2001;6(4):330-351.

71. Little RJ, D'Agostino R, Cohen ML, et al. The prevention and treatment of missing data in clinical trials. *The New England journal of medicine.* 2012;367(14):1355-1360.

72. Mallinckrodt CH. *Preventing and Treating Missing Data in Longitudinal Clinical Trials: A Practical Guide.* Cambridge: Cambridge University Press; 2013.

medRxiv preprint doi: https://doi.org/10.1101/2023.12.22.23300382; this version posted December 26, 2023. The copyright holder for this preprint (which was not certified by peer review) is the author/funder, who has granted medRxiv a license to display the preprint in perpetuity.

It is made available under a CC-BY-NC-ND 4.0 International license .

Improving Veteran Access to Integrated Management of Back Pain (AIM-Back): Updated Statistical Analysis Plan for this Embedded Pragmatic, Cluster Randomized

Cynthia J. Coffman, PhD (Center of Innovation to Accelerate Discovery and Practice Transformation, Durham VA Healthcare System, Department of Biostatistics and Bioinformatics, Duke University School of Medicine- (cynthia.coffman@duke.edu)

Rebecca North, PhD Center for the Study of Aging and Human Development, Duke University School of Medicine- Rebecca.North13@duke.edu

Steven Z. George PT, PhD, FAPTA (Duke Clinical Research Institute and Departments of Orthopaedic Surgery and Population Health Sciences, Duke University, Durham NC - steven.george@duke.edu)

Susan N. Hastings MD, MHSc (Durham Center of Innovation to Accelerate Discovery and Practice at Durham VAHCS, Geriatric Research, Education and Clinical Center at Durham VAHCS HSRD, Department of Population Health Sciences and Department of Medicine at Duke University, Durham NC - susan.hastings@duke.edu)

NOTE: This preprint reports new research that has not been certified by peer review and should not be used to guide clinical practice.

medRxiv preprint doi: https://doi.org/10.1101/2023.12.22.23300382; this version posted December 26, 2023. The copyright holder for this preprint (which was not certified by peer review) is the author/funder, who has granted medRxiv a license to display the preprint in perpetuity.

It is made available under a CC-BY-NC-ND 4.0 International license .

Abstract

The Improving Veteran Access to Integrated Management of Back Pain (AIM-Back) pragmatic, embedded, cluster-randomized trial is ongoing with enrollment starting in February 2020 and projected to end in first quarter of 2024. The 3-month follow up rate of primary outcome data collected as part of an AIM-Back clinical follow-up visit in the electronic health record has been lower than anticipated. At the recommendation of AIM-Back monitoring partners an updated statistical analysis plan was generated. The updated analysis plan allows for use of survey data to augment the follow up rate for the primary outcomes collected in the electronic health record. This updated statistical analysis plan was created and approved prior to completing enrollment and described in this paper.

medRxiv preprint doi: https://doi.org/10.1101/2023.12.22.23300382; this version posted December 26, 2023. The copyright holder for this preprint (which was not certified by peer review) is the author/funder, who has granted medRxiv a license to display the preprint in perpetuity.

It is made available under a CC-BY-NC-ND 4.0 International license .

Background

The Improving Veteran Access to Integrated Management of Back Pain (AIM-Back) pragmatic, embedded, cluster-randomized trial is ongoing with key elements of the study design, care pathways, primary outcomes and data analysis included in the published protocol paper.[1] AIM-Back was prospectively registered at www.clinicaltrials.gov (NCT04411420), began enrollment in February 2020, and is expected to continue enrollment into the first quarter of 2024. In response to a recommendation from our Data Safety Monitoring Board and in collaboration with study sponsor (National Center for Complementary and Integrative Health) and the NIH/DoD/VA Pain Management Collaboratory Biostatistics and Study Design Work Group we are updating our analysis plan prior to completing enrollment. The primary motivation for updating our statistical analysis plan is that we have observed a much lower than expected completion of 3-month follow-up where data is collected in the electronic health record (EHR) during AIM-back clinical visits. The updated statistical analysis plan was approved by our monitoring partners on December 18, 2023. Therefore, the purpose of this paper is to provide the rationale and justification for the updated data analysis plan, as well as to fully describe the updated analysis plan. The updated statistical analysis plan is only for our originally planned primary analyses and does not involve a change in the primary outcomes originally described in the protocol paper.

Ongoing Trial Progress Informing Updated Analysis Plan

The original power calculations for the this EHR sample were done assuming an 80% follow-up rate at 3-months, with 16 clinics enrolling n=105 at each clinic at baseline to have 84 per clinic at 3-months. The effect size difference we are powered to detect

medRxiv preprint doi: https://doi.org/10.1101/2023.12.22.23300382; this version posted December 26, 2023. The copyright holder for this preprint (which was not certified by peer review) is the author/funder, who has granted medRxiv a license to display the preprint in perpetuity.
It is made available under a CC-BY-NC-ND 4.0 International license .

assuming an ICC of 0.01 is 0.30 and for an ICC of 0.05 is 0.50. As of the end of September 2023, the overall 3-month EHR completion rate is 51% (52% in the sequenced care pathway (SCP) arm; 49% in pain navigator pathway (PNP) arm). With a 40% completion rate, n=42 at 3-months at each site with all the same assumptions as original power calculation, the effect size difference we can detect is 0.37 for an ICC of 0.01 and 0.55 for an ICC of 0.05. As the ICC is a nuisance parameter[2], we have calculated interim estimates of the ICC for our co-primary outcomes, PROMIS pain interference and physical function, at 3-months adjusted for baseline PROMIS pain interference and physical function, respectively. These calculations indicated they are comparable to our range for the original power calculations on the lower end of the range. To estimate the adjusted ICC, we fit a mixed model with the 3-month PROMIS outcome measure and adjusted for baseline PROMIS measure fitting a random effect for clinic, and used the covariance estimates for calculating the ICC. The estimated adjusted ICCs for the primary outcomes in the EHR sample and bootstrapped 95% confidence intervals are 0.014 with 95% CI of [0.00,0.046] for PROMIS physical function and 0.001 with 95% CI of [0.00,0.028] for PROMIS pain interference. The lower bound for the bootstrapped confidence interval for the ICC for both physical function and pain interference is 0.

During the trial we have enrolled and randomized 19 clinics; however, 2 clinics in block 2 withdrew from delivering the AIM-Back program after 8 months. Of those clinics that withdrew, 1 Veteran had enrolled with 3 referrals at one of the clinics and 5 Veterans enrolled with 9 referrals at the second clinic. For participating clinics the enrollment goal was to have clinics be in the 65-130 Veterans range with enrollment capped at n=130 to reduce variability in cluster sizes across clinics. As of the end of September 2023, n=1752

medRxiv preprint doi: https://doi.org/10.1101/2023.12.22.23300382; this version posted December 26, 2023. The copyright holder for this preprint (which was not certified by peer review) is the author/funder, who has granted medRxiv a license to display the preprint in perpetuity.
It is made available under a CC-BY-NC-ND 4.0 International license .

Veterans have enrolled with 12 clinics having met or exceeded the n=105 goal; 3 clinics have met minimum enrollment goals of n=65. We plan to complete enrollment in the first quarter of 2024 and project that 13-15 clinics will meet or exceed the n=105 enrollment goal. Current projections are that 2 clinics may be under the n=65 minimum goal.

Additional Data Source

We have collected the primary PROMIS outcomes for pain interference and physical function in the EHR. As described in our protocol paper[1], Veterans that are referred to the AIM-Back program by providers can also agree to be contacted for a survey study. These Veterans provide consent to participate, and are administered surveys that include the primary PROMIS measure outcomes and additional measures at baseline 3, 6, and 12 months follow-up. As of the end of September 2023, the overall 3-month and 6-month survey completion rate is 85% (83% in SCP arm; 87% in PNP arm) and 83% (84% in SCP arm; 81% in PNP arm), respectively. As of the end of September 2023 the number of Veterans in the survey study with baseline surveys were n=978 with n=764 completing 3-month and n=657 6-month surveys.

For the updated primary analysis, we will supplement the lower than expected 3-month EHR completion rate with the PROMIS measures collected from the survey when the 3- or 6-month follow-up survey is in the 2-4 month follow-up window for the EHR sample. In all cases, the 3-month EHR PROMIS measures and the survey measures are collected by telephone. In the EHR sample, data is collected in templated notes by clinical personnel at the clinic or from our central delivery team. Survey sample data is collected by blinded study personnel.

medRxiv preprint doi: https://doi.org/10.1101/2023.12.22.23300382; this version posted December 26, 2023. The copyright holder for this preprint (which was not certified by peer review) is the author/funder, who has granted medRxiv a license to display the preprint in perpetuity.
It is made available under a CC-BY-NC-ND 4.0 International license .

For the EHR sample, of those through 4-months of the study (n=1540), baseline demographic characteristics, rates of Centers for Disease Control and Prevention (CDC) high impact chronic pain and mean PROMIS scores for those with completed 3-month EHR follow-up compared to those missing EHR 3-months follow-up are shown in **Table 1.** Those missing 3-month follow-up are slightly younger, have higher rate of White race and slightly lower rates of CDC high impact chronic pain. Baseline PROMIS measures for pain interference, physical function and sleep are similar between those that completed 3-month vs. missing 3-month outcome. As of the end of September 2023, 44% of n=1752 enrolled subjects are a part of the survey sample (**Table 2**). Baseline characteristics and PROMIS survey measure scores for the EHR and Survey sample are shown in **Table 3**. Overall the survey sample is slightly younger, similar gender and White race distribution, with somewhat lower Black race representation in Survey sample than EHR sample and similar mean baseline PROMIS scores for pain interference, physical function and sleep disturbance.

There are 176 Veterans (75 in SCP arm; 101 in PNP arm) with missing EHR 3-month outcomes where at least 4-months have elapsed since their EHR baseline and have 3- or 6-month survey outcomes in the 2-4 month follow-up window (**Table 4**). Use of these data boosts the overall 3-month completion rate by 11% to 62% (increased to 63% for the SCP arm and 61% for the PNP arm). Baseline mean PROMIS scores by EHR-survey overlap are similar and shown in **Table 5**.

We did not need to reevaluate power as our initial power calculations for 90% power included a range of ICC values and we had effect size differences for both a lower sample size for survey outcomes and EHR outcomes (**Table 6**). We can use the effect size difference for the

medRxiv preprint doi: https://doi.org/10.1101/2023.12.22.23300382; this version posted December 26, 2023. The copyright holder for this preprint (which was not certified by peer review) is the author/funder, who has granted medRxiv a license to display the preprint in perpetuity.

It is made available under a CC-BY-NC-ND 4.0 International license .

survey outcomes, where n=42 at 3-months follow up as that would be a 60% attrition rate for the EHR sample – so the effect size differences we can detect for an ICC of 0.01 range from 0.30 – 0.37, for attrition rates ranging from 20% to 60% for the EHR Veterans. We used the sample size calculator from the NIH website (https://researchmethodsresources.nih.gov/) and **Table 7** shows the output from that calculator that yields a range of effect size differences for different numbers of clinics per arm as well as number of units per clinic needed at 3-months for an ICC of 0.01. Based on this table with our projected loss to follow-up adjusted based on using the survey data, we expect our loss to follow-up to be in the 35-40% range and with an ICC of 0.01 would give 90% power to detect effect size differences in the 0.32 -0.33 range.

Updated Analysis Plan

Primary Analyses (Aim 1)

The primary outcomes are continuous and will be ascertained at the planned baseline and follow-up assessment (3 months) from administrative data collected from the EHR on Veterans presenting at participating clinics that are referred and enrolled in AIM-BACK. For Veterans in the EHR sample that do not have the 3-month EHR outcome data within the 3-month follow-up window but have also participated in the survey study, we will supplement the 3-month EHR with the PROMIS measures collected from the survey when the 3- or 6-month follow-up survey is in the 2-4 month follow-up window for the EHR sample. In all cases, the 3-month EHR PROMIS measures and the survey measures are being collected by telephone. In the EHR sample, data is collected in templated notes in the EHR by clinical personnel onsite or clinical personnel from our central delivery team. Survey sample data is being collected by blinded study personnel.

medRxiv preprint doi: https://doi.org/10.1101/2023.12.22.23300382; this version posted December 26, 2023. The copyright holder for this preprint (which was not certified by peer review) is the author/funder, who has granted medRxiv a license to display the preprint in perpetuity.
It is made available under a CC-BY-NC-ND 4.0 International license .

Changes in pain interference and/or physical function scores will be estimated and the primary hypotheses tested via hierarchical linear mixed-effects models, with patients nested within clinics and baseline and 3 month values in the response vector.[3] Hierarchical linear models are a flexible and powerful analytic tool for clustered longitudinal continuous outcomes. The fixed-effect portion of the model will have the form: $Y_{ijk} = \beta_0 + \beta_1*(followup) + \beta_2*(followup*intervention)$ for clinic i, patient j, at time k. Random effects (clinics and time by clinics) will be included in the model to account for clustering of patients within clinics as the clinics are the unit of randomization. Random effects will also be included to account for the within-patient correlation between repeated measures over time. We will determine the best-fitting random effects structure by fitting a variety of random coefficient models (e.g., random intercept only, random intercept and linear slope) and assessing model performance with the Akaike Information Criteria (AIC) model selection criteria.[4, 5] The predictors in the model will include a time effect and indicator variables for treatment interacting with the time effect. The intraclass correlation capturing the relationship of outcomes between patients seen at the same clinic is accounted for via the random effects for the clinics and time by clinics, which are assumed to be normally distributed. The model will be fit in the SAS procedure PROC MIXED using full likelihood approximation and the hypotheses will be tested by whether the estimated coefficient β_2 is positive and significantly different than 0 at the 0.025 level due to 2 primary outcome variables. We will include covariates used in the covariate constrained randomization[6] (5 potential variables; average pain scores, clinic location (main medical center/community clinic), number of participating primary care providers, average level of opioid exposure of LBP patients at clinic, and average age of LBP patients at clinic) in

medRxiv preprint doi: https://doi.org/10.1101/2023.12.22.23300382; this version posted December 26, 2023. The copyright holder for this preprint (which was not certified by peer review) is the author/funder, who has granted medRxiv a license to display the preprint in perpetuity.
It is made available under a CC-BY-NC-ND 4.0 International license .

our primary model as well as a limited number of patient-level covariates that are readily available in the EHR (CDC pain, age, gender, race and a comorbidity measure).

The survey outcomes for the enrolled subset of patients will be collected at baseline, 3, 6, and 12 months and will include pain interference, function, intensity, catastrophizing, sleep, and depression. These are all longitudinal continuous outcomes and a hierarchical linear model similar to that described for the primary aim will be fit. We will fit random coefficient models as described above (e.g. random intercept only, random intercept and linear slope) and assess using AIC model selection criteria to determine the best model for the covariance structure. Similarly, we will determine the best model for the mean structure (e.g. linear, quadratic, dummy coding) as there are four outcome measurement occasions guided by descriptive plots and model fit assessed using AIC model selection criteria. Due to the timing of administration of baseline surveys – some baseline surveys may occur after the initial intervention contact - we will conduct a sensitivity analysis treating baseline surveys that occur after initial provider contact as occurring in the post-treatment period.

Sensitivity Analyses

We are updating and expanding our sensitivity analysis. We originally proposed a sensitivity analysis fitting models that include all EHR follow-up measurements at their follow-up time (even those outside the 3-month follow-up window) and then set up a contrast to estimate the treatment arm difference at 3-months from this model. We plan to expand on this analysis and, for the EHR sample, include all measurement time points (EHR and/or survey); the number of measurement occasions per subject will vary, ranging from a minimum of 1 (EHR baseline only) to a maximum of 8 measurement occasions

medRxiv preprint doi: https://doi.org/10.1101/2023.12.22.23300382; this version posted December 26, 2023. The copyright holder for this preprint (which was not certified by peer review) is the author/funder, who has granted medRxiv a license to display the preprint in perpetuity.
It is made available under a CC-BY-NC-ND 4.0 International license .

(**Table 8**), and be at unequal intervals. The zero-time point will be the EHR baseline, and survey time points and other EHR time points will be denoted as days from EHR baseline. As described above, we will fit random coefficient models and determine the best covariance and mean structure. A curve-fitting method (e.g., lowess, splines) will be used to estimate the average trends in outcomes and to inform the functional form of our mean trajectory model (e.g., linear or quadratic and other higher order terms). We will set up a contrast in this model to estimate the treatment difference at 3-months, the primary time point, as well as contrasts to examine treatment differences at longer follow-up times (e.g., 6-months).

Missing Data

We do not anticipate much missing data in the main predictors of interest, intervention arm, or patient characteristics available in the EHR or assessed at baseline in survey outcomes. There may be missing values in the outcome measures due to dropout, death, a missed interim assessment or an assessment outside the 1-month window for 3-month follow-up, or item non-response. However, hierarchical linear mixed models via maximum likelihood estimation, our main analysis technique for the primary outcomes, implicitly accommodate missingness at random (MAR).[3] Therefore, inferences will be valid even with differential dropout by intervention arm. We will thoroughly explore reasons for dropout and, depending upon the type and scope of missing data, we may explore the sensitivity of intervention effects to different missing data mechanisms (MAR vs. MNAR).[7] Our plan would be to conduct sensitivity analyses to evaluate the sensitivity of the assumption of MAR and/or MNAR on intervention effects. If our primary model

medRxiv preprint doi: https://doi.org/10.1101/2023.12.22.23300382; this version posted December 26, 2023. The copyright holder for this preprint (which was not certified by peer review) is the author/funder, who has granted medRxiv a license to display the preprint in perpetuity.
It is made available under a CC-BY-NC-ND 4.0 International license .

included design variables only (treatment, stratification variables, clinic), this model would meet the MAR assumptions if missing data is related to previous outcome assessments or design variables included in the primary model. We can assess the sensitivity of the MAR assumption by conducting an analysis that includes auxiliary variables (either as additional variables in primary models or by conducting a multiple imputation including auxiliary variables). These auxiliary variables or other baseline characteristics for the large sample may be limited due to availability in the EHR and will explore additional variables including baseline opioid exposure and area depravation index. We are currently proposing to include age, sex, race, CDC pain and a comorbidity index in our primary analysis that may strengthen our MAR assumption. To explore the MAR assumption, outcomes will be multiply imputed using principled methods in SAS (via PROC MI or IVEware).[8] Multiple imputation provides a framework for being able to incorporate information from important auxiliary variables while still preserving a parsimonious main treatment effect model and is described as a significant advantage in recommendations from Panel on Handling Missing Data in Clinical Trials.[9] Note that if needed, we will utilize imputation methods that account for the multiple levels of correlation inherent in the clustered data structure. If we cannot justify the assumption of MAR, we will explore the sensitivity of intervention effects to the MNAR assumption; we will follow guidelines in Mallinckrodt[10] and Ratitch et al[7] for model selection and pattern mixture modeling.

In contrast to an EHR study where all measurements are determined by clinical practice and do not follow a data collection schedule, the AIM-Back program has a data collection schedule that has been implemented in the clinical process with outcome assessments documented in the EHR. Thus, standard missing data techniques for missing

medRxiv preprint doi: https://doi.org/10.1101/2023.12.22.23300382; this version posted December 26, 2023. The copyright holder for this preprint (which was not certified by peer review) is the author/funder, who has granted medRxiv a license to display the preprint in perpetuity.
It is made available under a CC-BY-NC-ND 4.0 International license .

outcome measurements can be applied for those enrolled, as described above. However, because Veterans are referred to the AIM-BACK program by providers and must attend the first AIM-BACK visit to be enrolled, the potential for selection bias exists with a significant number of Veterans referred that are not enrolled and have no outcome data.

Through September 2023, we have approximately 2700 referrals with over 1700 Veterans enrolled in the program; approximately 70% of referrals led to enrollment in the AIM-BACK program with around 30% of referrals discontinued or cancelled across clinics. In monthly reports during the course of our study, we have been examining differences in CDC pain, age, gender, race, and ethnicity of Veterans referred and enrolled in the program to Veterans referred that do not enroll in the program. Overall to date, the patient demographics and CDC pain rates are similar between those enrolled and not enrolled. However, as we have no outcome data on those that do not enroll, in sensitivity analyses we will use inverse probability weighting methods (IPW) to adjust for this selection bias.[11] Probability weights for the probability of enrolling in the AIM-BACK program will be generated by fitting a logistic regression model to the binary enrollment indicator, with covariates for patient EHR demographics, and CDC pain at time of referral; we will explore whether adjustment for clinic is needed either as a fixed or random effect. We will adjust our primary model described above with probability weights and estimate treatment effects at 3-months.

Conclusion

An updated plan for the primary analysis for the AIM-Back trial has been created and was approved by our monitoring partners on December 18, 2023. The need for an

medRxiv preprint doi: https://doi.org/10.1101/2023.12.22.23300382; this version posted December 26, 2023. The copyright holder for this preprint (which was not certified by peer review) is the author/funder, who has granted medRxiv a license to display the preprint in perpetuity.

It is made available under a CC-BY-NC-ND 4.0 International license .

updated analysis plan was driven by the lower than projected follow-up rates for the primary outcome in the EHR observed during the AIM-Back trial. The updated plan does not change the primary outcome measures, but allows for clinical data to be supplemented by survey data that occurs within a 1 month timeframe around the 3-month endpoint. Additionally, we present updated models for our sensitivity and missing data analyses. This updated analysis plan, along with the previously published protocol paper[1], are key source documents for evaluating the primary results of care pathway effectiveness from the AIM-Back trial.

medRxiv preprint doi: https://doi.org/10.1101/2023.12.22.23300382; this version posted December 26, 2023. The copyright holder for this preprint (which was not certified by peer review) is the author/funder, who has granted medRxiv a license to display the preprint in perpetuity.
It is made available under a CC-BY-NC-ND 4.0 International license .

Table 1: Baseline demographic characteristics, CDC high impact chronic pain rate and PROMIS measures of EHR sample (n=1752) and Survey sample (n=978) for those completed EHR 3-month follow-up vs. missing 3-month EHR follow-up

Baseline Characteristic/Measure	Completed 3-month N=777	Missing 3-month N=763
**Female, n(%)**	107(13.8)	99(13.0)
**Race, n(%)**		
White	450(57.9)	480(62.9)
Black	262(33.7)	207(27.1)
Other	14(1.8)	20(2.6)
Missing	51(6.7)	56(7.3)
**Hispanic Ethnicity**	27(3.5)	44(5.8)
**Age, mean(SD)**	53.54(15.3)	51.56(15.9)
**CDC High Impact Chronic pain, n(%)**		
Referral	596(76.7)	585(76.7)
Baseline	514(66.2)	491(64.4)
**PROMIS Pain Interference, mean(SD)**	63.28(6.9)	62.80(6.7)
**PROMIS Physical Function, mean(SD)**	36.45(4.7)	36.98(4.7)
**PROMIS Sleep, mean(SD)**	58.23(8.2)	58.45(8.6)

medRxiv preprint doi: https://doi.org/10.1101/2023.12.22.23300382; this version posted December 26, 2023. The copyright holder for this preprint (which was not certified by peer review) is the author/funder, who has granted medRxiv a license to display the preprint in perpetuity.

It is made available under a CC-BY-NC-ND 4.0 International license .

Table 2: Distribution of baseline survey in EHR enrolled sample by treatment arm

ARM	In EHR Not in Survey	In EHR, In survey	Total EHR enrolled
Arm 1	604	380	984
	61.3%	38.6%	
Arm 2	386	382	768
	50.3%	49.7%	
Total	990	762	1752
	54.2%	43.5%	

Table 3: Baseline characteristics and PROMIS measures of EHR sample (n=1752) and Survey sample (n=978)

	**EHR Sample**			**Survey Sample**		
**Patient Characteristics (from trial enrollment)**	**Arm 1 (N=984)**	**Arm2 (N=768)**	**Total (N=1752)**	**Arm 1 (N=482)**	**Arm2 (N=496)**	**Total (N=978)**
Age, mean (SD)	52.6(15.9)	53.2(15.3)	52.8(15.6)	52.1 (15.5)	51.4 (15.2)	51.7 (15.3)
Sex– Female, N (%)	109(11.1)	109(14.2)	218(12.4)	48 (10.0)	65 (13.1)	113 (11.5)
Race, N (%)						
Black or African American	223(22.7)	286(37.2)	509(29.1)	90 (18.7)	163 (32.9)	254 (25.9)
White	664(67.5)	415(54.0)	1079(61.6)	317 (65.8)	285 (57.5)	602 (61.5)
Ethnicity, N (%)						
Hispanic or Latino	54(5.5)	28(3.6)	82(4.7)	85 (8.7)	49 (10.2)	36 (7.3)
**PROMIS Measures**						
Pain Interference, Mean (SD) N	63.0 (6.54) 956	63.0 (7.20) 766	63.0 (6.84) 1722	63.1 (6.61) 467	63.3 (6.68) 474	63.2 (6.64) 941
Physical Function, Mean (SD) N	37.0 (5.12) 958	37.3 (5.68) 764	37.1 (5.37) 1722	37.9 (5.58) 476	38.2 (5.87) 492	38.1 (5.73) 968
Sleep Disturbance Mean (SD) N	58.5 (8.31) 956	58.1 (8.49) 766	58.3 (8.39) 1722	58.7 (7.76) 479	58.5 (7.82) 489	58.6 (7.79) 968

medRxiv preprint doi: https://doi.org/10.1101/2023.12.22.23300382; this version posted December 26, 2023. The copyright holder for this preprint (which was not certified by peer review) is the author/funder, who has granted medRxiv a license to display the preprint in perpetuity.

It is made available under a CC-BY-NC-ND 4.0 International license .

Table 4: For Veterans that have an EHR baseline and through 4 months of study (n=1540) as of 9/30/2023 the distribution of 3-month outcomes from EHR and 3- and 6-months surveys

ARM	IN EHR, Completed 3-month	IN HER, Completed 3-month with survey boost	In EHR, no 3-M EHR, no survey or no survey in window	In EHR, no 3M EHR, 3-month survey in window	In EHR, no 3M EHR, 6-month survey in window	Total	
ARM 1	401	502	318	96	5	101	820
	48.9%	61.2%	38.7%	11.7%	0.6%	12.3%	
ARM 2	376	451	269	68	7	75	720
	52.2%	62.6%	37.4%	9.4%	1.0%	10.4%	
Total	777	953	587	164	12	176	1540
	50.5%	61.8%	38.1%	10.6%	0.8%	11.4%	

medRxiv preprint doi: https://doi.org/10.1101/2023.12.22.23300382; this version posted December 26, 2023. The copyright holder for this preprint (which was not certified by peer review) is the author/funder, who has granted medRxiv a license to display the preprint in perpetuity.

It is made available under a CC-BY-NC-ND 4.0 International license .

Table 5. Baseline PROMIS scores by EHR – Survey Overlap

EHR – Survey Overlap	EHR Mean(SD)	Survey Mean(SD)
**In EHR, In Survey (n=763)**		
Pain Interference	62.8(7.0)	63.1(6.7)
Physical Function	36.7(4.6)	38.0(5.7)
Sleep	58.7(8.2)	58.3(7.8)
**In EHR, Not In Survey (n=989)**		
Pain Interference	62.8(7.0)	-
Physical Function	36.7(5.0)	-
Sleep	58.0(8.6)	-
**Not in EHR, In Survey (n=200)**		
Pain Interference	-	63.6(6.6)
Physical Function	-	38.3(5.8)
Sleep	-	59.9(7.8)

medRxiv preprint doi: https://doi.org/10.1101/2023.12.22.23300382; this version posted December 26, 2023. The copyright holder for this preprint (which was not certified by peer review) is the author/funder, who has granted medRxiv a license to display the preprint in perpetuity.

It is made available under a CC-BY-NC-ND 4.0 International license .

Table 6. Power Table for AIM-BACK

Number of clinics per condition	Number of patients per clinic needed at post-intervention follow-up (Total N)	Df for baseline covariate adjustment	Minimum Detectable Difference (Effect size) ICC=0.01	Minimum Detectable Difference (Effect size) ICC=0.02	Minimum Detectable Difference (Effect size) ICC=0.05	Number needed at baseline with 20% attrition per clinic (Total N_b)
8	42 (N=672)	5<br>2	0.37<br>0.36	0.42<br>0.41	0.54<br>0.53	53<br>(Nb=848)
8	84<br>(N=1344)	5<br>2	0.30<br>0.29	0.36<br>0.35	0.50<br>0.49	105<br>(Nb=1680)

Table key: Effect sizes differences between arms based on the net difference between the two study conditions across two points in time (baseline and 3-months) at 3-months follow-up for 90% power, an alpha of 0.025, within-subject correlation = 0.50, standard deviation (SD) of outcome=1, adjustment for baseline covariates (max of 4 group level covariates based on constrained randomization that could take up between 2 and 5 degrees of freedom; we conservatively estimated no reduction in variance with inclusion of baseline covariates) and intraclass correlation coefficients (ICC) of 0.01, 0.02 and 0.05.

medRxiv preprint doi: https://doi.org/10.1101/2023.12.22.23300382; this version posted December 26, 2023. The copyright holder for this preprint (which was not certified by peer review) is the author/funder, who has granted medRxiv a license to display the preprint in perpetuity.

It is made available under a CC-BY-NC-ND 4.0 International license .

Table 7. Output table from NIH Website Power Calculator for an ICC=0.01

Standardized Detectable Difference						
Groups Per Condition						
		7	8	9	10	11
	42	0.4168	0.3727	0.3417	0.3182	0.2993
Members	63	0.3648	0.3262	0.2991	0.2785	0.2619
Per	84	0.3358	0.3002	**0.2753**	0.2563	0.2277
Group	105	0.3171	0.2835	0.2600	0.2420	0.2277
	126	0.3040	0.2718	0.2492	0.2320	0.2183

Table Key: Effect sizes calculated using Research Methods Resources: National Institutes of Health. Available from: https://researchmethodsresources.nih.gov/

medRxiv preprint doi: https://doi.org/10.1101/2023.12.22.23300382; this version posted December 26, 2023. The copyright holder for this preprint (which was not certified by peer review) is the author/funder, who has granted medRxiv a license to display the preprint in perpetuity.

It is made available under a CC-BY-NC-ND 4.0 International license .

Table 8: Data patterns for veterans referred by primary care providers at enrolled clinics to AIM-BACK pathway including baseline 3, 6 and 12-months survey.

If do not agree to be contacted for survey, possible patterns are

PNP arm – primary outcomes are collected at baseline and 3-month only in the EHR in this arm

Referral	Survey baseline	EHR baseline	Survey 3-month	EHR 3-month	Survey 6-month	Survey 12-month
x						
x		x				
x		x		x		

SCP arm – primary outcomes are collected at baseline, 6-weeks and 3-months in the EHR in this arm

Referral	Survey baseline	EHR baseline	EHR 6-week	Survey 3-month	EHR 3-month	Survey 6-month	Survey 12-month
x							
x		x	x				
x		x					
x		x			x		
x		x	x		x		

If agree to be contacted for survey, possible patterns are

PNP ARM (light blue fill have referral only; light grey fill have EHR only; grey fill have EHR and survey, white fill have survey only)

Referral	Survey baseline	EHR baseline	Survey 3-month	EHR 3-month	Survey 6-month	Survey 12-month
x						
x		x				
x		x		x		
x	x	x				
x	x	x	x			
x	x	x		x		
x	x	x	x	x		
x	x	x	x		x	
x	x	x	x			x
x	x	x	x		x	x
x	x	x		x	x	
x	x	x		x		x
x	x	x		x	x	x
x	x	x	x	x	x	

medRxiv preprint doi: https://doi.org/10.1101/2023.12.22.23300382; this version posted December 26, 2023. The copyright holder for this preprint (which was not certified by peer review) is the author/funder, who has granted medRxiv a license to display the preprint in perpetuity.
It is made available under a CC-BY-NC-ND 4.0 International license .

x	x	x	x	x		x
x	x	x	x	x	x	x
x	x					
x	x		x			
x	x		x		x	
x	x		x			x
x	x		x		x	x
x	x				x	
x	x					x
x	x				x	x

SCP Arm (light blue fill have referral only; light grey fill have EHR only; grey fill have EHR and survey, white fill have survey only)

Referral	Survey baseline	EHR baseline	EHR 6-week	Survey 3-month	EHR 3-month	Survey 6-month	Survey 12-month
x							
x		x	x				
x		x					
x		x			x		
x		x	x		x		
x	x	x					
x	x	x			x		
x	x	x			x	x	
x	x	x			x		x
x	x	x			x	x	x
x	x	x				x	
x	x	x				x	x
x	x	x					x
x	x	x	x				
x	x	x	x		x		
x	x	x	x		x	x	
x	x	x	x		x		x
x	x	x	x		x	x	x
x	x	x	x			x	
x	x	x	x			x	x
x	x	x	x				x
x	x	x		x			
x	x	x		x	x		
x	x	x		x	x	x	
x	x	x		x	x		x
x	x	x		x	x	x	x
x	x	x		x		x	
x	x	x		x		x	x
x	x	x		x			x

medRxiv preprint doi: https://doi.org/10.1101/2023.12.22.23300382; this version posted December 26, 2023. The copyright holder for this preprint (which was not certified by peer review) is the author/funder, who has granted medRxiv a license to display the preprint in perpetuity.

It is made available under a CC-BY-NC-ND 4.0 International license .

X	X	X	X	X			
X	X	X	X	X	X		
X	X	X	X	X	X	X	
X	X	X	X	X	X		X
X	X	X	X	X	X	X	X
X	X	X	X	X		X	
X	X	X	X	X		X	X
X	X	X	X	X			X
X	X						
X	X			X			
X	X			X		X	
X	X			X			X
X	X			X		X	X
X	X					X	
X	X						X
X	X					X	X

medRxiv preprint doi: https://doi.org/10.1101/2023.12.22.23300382; this version posted December 26, 2023. The copyright holder for this preprint (which was not certified by peer review) is the author/funder, who has granted medRxiv a license to display the preprint in perpetuity.

It is made available under a CC-BY-NC-ND 4.0 International license .

References

1. George SZ, Coffman CJ, Allen KD, Lentz TA, Choate A, Goode AP, Simon CB, Grubber JM, King H, Cook CE *et al*: **Improving Veteran Access to Integrated Management of Back Pain (AIM-Back): Protocol for an Embedded Pragmatic Cluster-Randomized Trial**. *Pain Med* 2020, **21**(Suppl 2):S62-s72.
2. Hemming K, Martin J, Gallos I, Coomarasamy A, Middleton L: **Interim data monitoring in cluster randomised trials: Practical issues and a case study**. *Clin Trials* 2021, **18**(5):552-561.
3. Gibbons Ha: **Longitudinal Data Analysis.** Hoboken, NJ: Wiley & sons; 2006.
4. Akaike H: **Information Theory and an Extension of the Maximum Likelihood Principle**. In: *Selected Papers of Hirotugu Akaike.* edn. Edited by Parzen E, Tanabe K, Kitagawa G. New York, NY: Springer New York; 1998: 199-213.
5. Burnham KP, Anderson DR: **Model Selection and Multimodel Inference**, 2 edn: Springer-Verlag New York; 2002.
6. Li F, Turner EL, Heagerty PJ, Murray DM, Vollmer WM, DeLong ER: **An evaluation of constrained randomization for the design and analysis of group-randomized trials with binary outcomes**. *Statistics in medicine* 2017, **36**(24):3791-3806.
7. Ratitch B, O'Kelly M, Tosiello R: **Missing data in clinical trials: from clinical assumptions to statistical analysis using pattern mixture models**. *Pharmaceutical statistics* 2013, **12**(6):337-347.
8. Collins LM, Schafer JL, Kam CM: **A comparison of inclusive and restrictive strategies in modern missing data procedures**. *Psychological methods* 2001, **6**(4):330-351.
9. Little RJ, D'Agostino R, Cohen ML, Dickersin K, Emerson SS, Farrar JT, Frangakis C, Hogan JW, Molenberghs G, Murphy SA *et al*: **The prevention and treatment of missing data in clinical trials**. *The New England journal of medicine* 2012, **367**(14):1355-1360.
10. Mallinckrodt CH: **Preventing and Treating Missing Data in Longitudinal Clinical Trials: A Practical Guide**. Cambridge: Cambridge University Press; 2013.
11. Peskoe SB, Arterburn D, Coleman KJ, Herrinton LJ, Daniels MJ, Haneuse S: **Adjusting for selection bias due to missing data in electronic health records-based research**. *Stat Methods Med Res* 2021, **30**(10):2221-2238.
