# Supplementary material for: Sequenced Care Pathway vs Pain Navigator Pathway for Veterans With Low Back Pain: The AIM-Back Cluster Randomized Clinical Trial
Source: JAMA Netw Open. 2026 Apr 2;9(4):e264421. doi: 10.1001/jamanetworkopen.2026.4421 (PMC13047465; doi:10.1001/jamanetworkopen.2026.4421)
Supplement: Supplement 2. — eAppendix 1. Statistical Analysis Methods eAppendix 2. Secondary and Sensitivity Results eAppendix 3. Adherence Results eAppendix 4. Adverse Events for Survey Participants eAppendix 5. SAS Code for Fitting Hierarchical Linear Models in PROC MIXED eFigure 1. AIM-Back CONSORT eTable 1. Pathway Descriptions eTable 2. Patient-Reported Outcome Measures and Measurement Properties (CONSORT-PRO) eTable 3. Patient Characteristics From Survey at Baseline eTable 4. Baseline Characteristics of EHR Patients by 3M Outcome Missingness eFigure 2a. Enrolled Participants (n=1817) With All Time Points (EHR and Survey) for PROMIS-SF Pain Interference eFigure 2b. Enrolled Participants (n=1817) With All Time Points (EHR and Survey) for PROMIS-SF Physical Function eFigure 3. Estimated Means (Blue Text SCP; Red Text PNP) and Mean Difference (Black Text) at Follow-Up Time Points for PROMIS Pain Interference and Physical Functions Outcomes and Associated 97.5% Confidence Intervals for Enrolled Participants (n=1817) From Multiply Imputed Data With Combined Estimates Across n=50 Imputation Fit to Hierarchical Linear Mixed Models eFigure 4. Estimated Means (Blue Text SCP; Red Text PNP) and Mean Difference (Black Text) at Follow-Up Time Points for PROMIS Pain Interference and Physical Functions Outcomes and Associated 97.5% Confidence Intervals for Enrolled Participants (n=1817) From IPW Models Adjusted for Referral Bias Fit to Hierarchical Linear Mixed Models eFigure 5. Estimated Means (Blue Text SCP; Red Text PNP) and Mean Difference (Black Text) at Follow-Up Time Points for PROMIS Sleep Disturbance and NIH Pain Intensity Outcomes and Associated 95% Confidence Intervals for Enrolled Participants (Primary; n=1817) and Survey Participants (n=799) From Hierarchical Linear Mixed-Effects Models eTable 5. Estimated Means and Mean Differences at Follow-Up Time Points for Secondary Outcomes and Associated 95% Confidence Intervals for Survey Participants (n=799) From Hierarchical Linear Mixed-Effects M [file jamanetwopen-e264421-s002.pdf]

Supplementary Online Content

George SZ, Coffman CJ, North R, et al. Sequenced care pathway vs pain navigator pathway for veterans with low back pain: the AIM-Back cluster randomized clinical trial. *JAMA Netw Open.* 2026;9(4):e264421. doi:10.1001/jamanetworkopen.2026.4421

eAppendix 1. Statistical Analysis Methods
eAppendix 2. Secondary and Sensitivity Results
eAppendix 3. Adherence Results
eAppendix 4. Adverse Events for Survey Participants
eAppendix 5. SAS Code for Fitting Hierarchical Linear Models in PROC MIXED
eFigure 1. AIM-Back CONSORT
eTable 1. Pathway Descriptions
eTable 2. Patient-Reported Outcome Measures and Measurement Properties (CONSORT-PRO)
eTable 3. Patient Characteristics From Survey at Baseline
eTable 4. Baseline Characteristics of EHR Patients by 3M Outcome Missingness
eFigure 2a. Enrolled Participants (n=1817) With All Time Points (EHR and Survey) for PROMIS-SF Pain Interference
eFigure 2b. Enrolled Participants (n=1817) With All Time Points (EHR and Survey) for PROMIS-SF Physical Function
eFigure 3. Estimated Means (Blue Text SCP; Red Text PNP) and Mean Difference (Black Text) at Follow-Up Time Points for PROMIS Pain Interference and Physical Functions Outcomes and Associated 97.5% Confidence Intervals for Enrolled Participants (n=1817) From Multiply Imputed Data With Combined Estimates Across n=50 Imputation Fit to Hierarchical Linear Mixed Models
eFigure 4. Estimated Means (Blue Text SCP; Red Text PNP) and Mean Difference (Black Text) at Follow-Up Time Points for PROMIS Pain Interference and Physical Functions Outcomes and Associated 97.5% Confidence Intervals for Enrolled Participants (n=1817) From IPW Models Adjusted for Referral Bias Fit to Hierarchical Linear Mixed Models
eFigure 5. Estimated Means (Blue Text SCP; Red Text PNP) and Mean Difference (Black Text) at Follow-Up Time Points for PROMIS Sleep Disturbance and NIH Pain Intensity Outcomes and Associated 95% Confidence Intervals for Enrolled Participants (Primary; n=1817) and Survey Participants (n=799) From Hierarchical Linear Mixed-Effects Models
eTable 5. Estimated Means and Mean Differences at Follow-Up Time Points for Secondary Outcomes and Associated 95% Confidence Intervals for Survey Participants (n=799) From Hierarchical Linear Mixed-Effects Models
eReferences.

This supplementary material has been provided by the authors to give readers additional information about their work.

© 2026 George SZ et al. *JAMA Network Open.*

eAppendix 1. Statistical Analysis Methods

Covariate Constrained Randomization Covariates

The patient-level covariates were averages of characteristics of patients with visits to clinics with LBP ICD-10 codes in the 6 months prior to enrollment date for the block and included 1) pain scale scores, 2) level of opioid exposure, and 3) age. The clinic-level covariates included: 1) number of participating primary care providers, and 2) location (main medical center/community clinic).

Patient covariates

Demographic and clinical information, PTSD diagnoses, and area deprivation index (ADI) in the year prior to referral, and Care Assessment Need (CAN) scores prior to referral date (closest) were extracted from CDW. The ADI is a measure of socioeconomic disadvantage in a geographic region, ranging from 1-100 with higher values indicating more disadvantage.[1] The CAN score,[2] a comorbidity measure, is a risk percentile based on the estimated risk for hospital admission or death within a year calculated weekly for all eligible Veterans. The score ranges from 0 (lowest risk) to 99 (highest risk). Opioid and benzodiazepine use were extracted from pharmacy refill data in CDW.

Primary outcomes

T-scores were used for PROMIS measures using response pattern scoring with RedCAP to score PROMIS-SF measures missing 1 item. If more than 1-item was missing, scores were set to missing.

For the small number of enrolled subjects that had multiple EHR follow-up visits we used the outcome data from the visit if it fell in the follow-up window or if both were in the follow-up window outcome data from the first visit was used.

All available time points

We included all measurement timepoints for the enrolled participants (EHR and survey; range from 1 to 8 per patient) at unequal intervals. The zero timepoint was when Veterans completed enrollment into the program establishing their EHR baseline; all other timepoints were denoted as days from EHR baseline. Survey outcomes collected more than 60 days prior to EHR baseline were excluded (n=43). Hierarchical penalized spline mixed-effects models were fit using piecewise linear functions with prespecified knots included as fixed and random effects at the population-average level.[3] Linear time with knots at 3, 6, and 9 months interacting with treatment were included with random effects for clinics, clinic by time, and patient level random intercept and slopes.

Multiple Imputation

We conducted a sensitivity analysis for the primary analysis (n=1817) using a multiple imputation by fully conditional specification (chained equations) with the R-package *mice*[4] that included additional baseline variables beyond those in our primary models to strengthen the MAR assumption and accounted for the clustering of patients within clinic.[5] We used the *2l.pan mice* package that applies a two-level normal model with homogeneous within group variances. We have limited baseline patient-level covariates available on enrolled participants from EHR above those that were defined *a priori* (CDC high impact chronic pain status at baseline (HICP), age, gender, race and CAN score) included in primary models. The additional patient-level covariates included in the multiple imputation were NIH chronicity, chronic opioid use, PTSD diagnosis in year prior to referral and ADI. We conduced n=50 imputations as the highest fraction of missing information was approximately 50%.

Referral bias

To address the potential effect of selection bias of enrolled participants (n=1817) on our primary results as providers refer patients to AIM-BACK but not all attend an AIM-BACK visit (enroll), inverse probability weights (IPW) were used to adjust for this referral bias.[6] Probability weights for the probability of enrolling in AIM-BACK after referral were generated by fitting a logistic regression model to the binary enrollment indicator by clinic, with covariates for patient EHR demographics, opioid use, CAN score, HICP pain at time of referral, ADI, and PTSD diagnosis (see Table S3) using PROC PSMATCH in SAS version 9.4. Propensity scores were estimated separately by clinic to account for differing clinic populations; the clinic specific propensity scores reflect variation in clinic populations

and operations. For each clinic, to insure comparability of groups we selected all observations that were in the region of common support (sufficient overlap in propensity scores defined by the largest interval that contains propensity scores for subjects for both groups (enrolled and not enrolled); n= 43 patients were excluded across 13 of 17 clinics that were outside the region of common support. Observations that were in the region of common support for each clinic were used (sufficient overlap in propensity scores) and stabilized weights were used. To assess balance of measured covariates we examined standardized differences and inspected estimated distribution of propensity scores.[7] We included IPW in primary models described in main paper and estimated treatment effects at 3-months.

Adherence metrics

Patient level metrics were used to describe selected delivery components for each pathway. For the SCP, the potential range for number of sessions attended depended on SBST risk stratification (1-9 for low-risk and 1-15 for medium/high risk). We also described whether pain modulation was used and/or pain neuroscience education was delivered in the SCP initial and/or 6-week follow-up visit. For the PNP the potential range for number of sessions attended range from 1 to 5 depending on number of referrals documented at the initial and/or 6-week follow-up visit. We also described for those who attended 3-month follow-up visits, whether they received the pain navigator recommended services based on documented self-report data in EHR.

Due to the variability in types and number of visits/sessions attended (pathway delivery tracking), tests for differences in these metrics between pathways were not conducted. The goal in describing these metrics by pathway was to gain insight into where to target clinical programs and focus efforts to improve patient access to and engagement with non-pharmacologic pain services.

eAppendix 2. Secondary and Sensitivity Results

SECONDARY RESULTS

For the survey sample the estimated ICC for PROMIS-SF pain interference was 0.005 and for physical function was 0.003.

SENSITIVITY ANALYSIS RESULTS

All available time points

In the analysis for the enrolled participants (n=1817) using all available outcome measures (EHR and survey, min of 1 (EHR baseline only), max of 8 measurements), 43 observations were excluded that were more than 60 days prior to the EHR baseline (see Figure S2a A). The mean number of measurements per patient was 3.7 with 82.9% having 2 or more measurements. For the co-primary PROMIS-SF outcomes pain interference and physical function there were no differences between arms at 3-months, 6-months and 12-months (see Figures S1a, S1b). Estimated differences at 3-months were similar to estimates from primary analysis models with estimated difference for pain interference of -0.5 points (97.5% CI, -1.6 to 0.4) for SCP vs. PNP and for physical function of 0.3 points (97.5% CI, -0.3 to 1.5; p= 0.139) (see Figures S2a S2b).

Multiple imputation

Estimated differences at 3-months were similar to estimates from primary analysis models with estimated difference for pain interference of -0.5 points (97.5% CI, -1.4 to 0.5, p=0.28) for SCP vs. PNP and for physical function of 0.5 points (97.5% CI, -0.3 to 1.3; p= 0.159) (Figure S2).

Referral bias

Characteristics of the Referral Cohort (n=2767) are shown in Table S3. In the analysis to address the potential effect of referral bias of enrolled participants (n=1817) on the co-primary PROMIS-SF outcomes pain interference and physical function there were no differences between arms at 3-months. Estimated differences at 3-months were similar to estimates from primary analysis models with estimated difference for pain interference of -0.6 points (97.5% CI, -1.6 to 0.4; p=0.16) for SCP vs. PNP and for physical function of 0.4 points (97.5% CI, -0.5 to 1.3; p= 0.31) (Figure S3).

eAppendix 3. Adherence Results

Uptake of the AIM-Back pathways was high with 17/19 (89.4%) clinics completing the implementation training and successfully delivering their randomly assigned pathway. Of the 17 clinics that implemented their assigned pathway, 16 (94.1%) met minimum recruitment goals of n = 65 Veterans enrolled with lowest enrolling clinics of n = 41 and n = 82 for SCP and PNP, respectively. Fourteen clinics (82.3%) enrolled at least 100 Veterans with highest enrolling clinics of n = 129 for both SCP and PNP.

In the SCP, Veterans stratified to low risk with SBST at 6-weeks or with missing SBST scores (n=434) completed a mean (SD) of 3.7 (2.3) sessions of a possible 9. Of Veterans who attended the 6-week visit and were stratified to medium/high risk (n=377, 70.9%), a mean (SD) of 7.5 (3.4) sessions of a possible 15 were completed. In the initial visit, 91.9% (n=745) received pain modulation or pain neuroscience education. Veterans in the SCP completed a mean (SD) of 2.4 (1.9) physical activity (PA) sessions out of 6 (see Table 1). Veterans in the PNP completed a mean (SD) of 4.3 (0.7) sessions of a possible 5. In the initial and 6-week visits, 94.9% (n=955) and 70.8% (n=413) of Veterans were referred to at least one service with PT, chiropractic, and acupuncture, the 3 most frequently referred services (see Table 2). For those who completed the 3-month follow-up visit, 80.7% (n=381) indicated receipt of referred service either within or outside VA.

© 2026 George SZ et al. *JAMA Network Open.*

eAppendix 4. Adverse Events for Survey Participants

Clinical pathways were delivered as part of routine care so adverse events were only collected for Veterans who consented to survey completion. Adverse events were categorized as death, hospitalizations, and important medical events (e.g. ED visits) and were documented as required by the DSMB). There were 83 instances of adverse events among the 1069 consented Veterans contacted to participate in the surveys, none were survey related.

© 2026 George SZ et al. *JAMA Network Open.*

eAppendix 5. SAS Code for Fitting Hierarchical Linear Models in PROC MIXED

```
*Clinic and Patient-level covariates were centered with mean 0;

proc mixed data=analyze covtest;
    class clinic_id patient_id time RandomArm(ref="PNP");
            model &outcome. = ThreeMonths RandomArm*Threemonths &covars.
            Clinic_std_ClinicType_n Clinic_std_numberproviders Clinic_std_meanage1 Clinic_std_meanavgpain1
            Clinic_std_meanopioid1
            Patient_std_gender_n Patient_std_imp_race_n Patient_std_baseCDC Patient_std_ageatimport
            Patient_std_imp_baseCAN
              / solution ddfm=kr;
            random intercept time / subject=clinic_id;
            repeated time / subject=patient_id type=un;
            ods output Nobs=nobs Estimates=est;
            estimate 'Baseline Overall Mean' intercept 1 Threemonths 0 RandomArm* Threemonths 0 0 / cl alpha=
            0.025;
            estimate 'SCP 3M Mean' intercept 1 Threemonths 1 RandomArm*Threemonths 1 0 / cl alpha= 0.025;
            estimate 'PNP 3M Mean' intercept 1 Threemonths 1 RandomArm*Threemonths 0 1 / cl alpha= 0.025;
            estimate '3M Mean Difference' intercept 0 Threemonths 0 RandomArm* Threemonths 1 -1 / cl alpha=
            0.025
            estimate 'SCP 3M Change' intercept 0 Threemonths 1 RandomArm*Threemonths 1 0 / cl alpha= 0.025
            estimate 'PNP 3M Change' intercept 0 Threemonths 1 RandomArm*Threemonths 0 1 / cl alpha= 0.025.;
            run;
```

© 2026 George SZ et al. *JAMA Network Open.*

eFigure 1. AIM-Back CONSORT

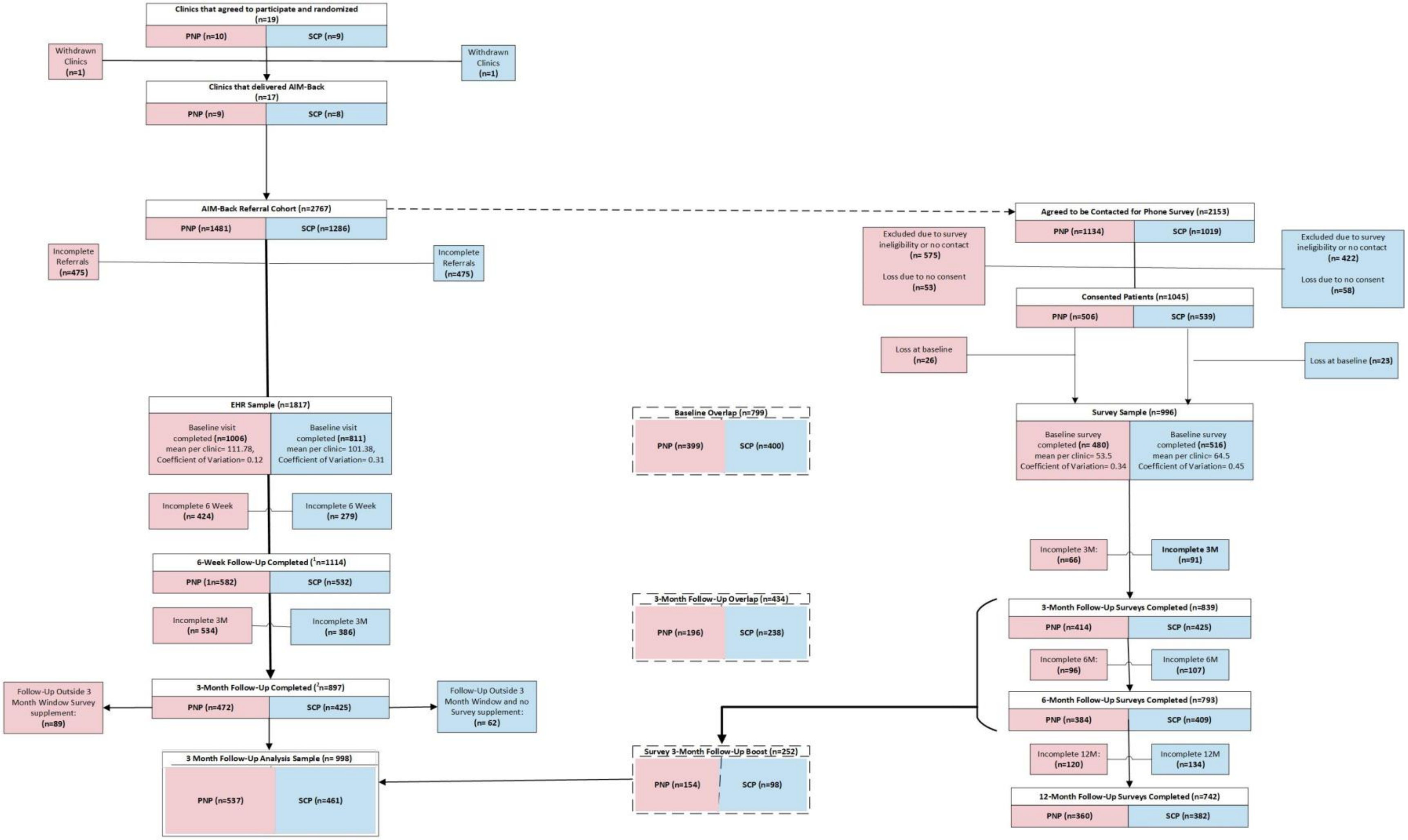

[1] n=35 Veterans with multiple 6-week follow-up visits in PNP arm (32 with 2 visits, 3 with 3) and n=18 in SCP arm (17 with 2 visits, 1 with 3)
[2] n=8 Veterans with two 3-month follow-up visits in PNP arm and n=1 in SCP arm

© 2026 George SZ et al. *JAMA Network Open.*

eTable 1. Pathway Descriptions

Timepoint	Sequence Care Pathway (SCP) Activities	Provider Encounter Type	Pain Navigator Pathway (PNP) Activities	Provider Encounter Type
**Referral to AIM-Back**	Veterans meet with their Primary Care Provider and are referred to begin the AIM-Back program.	Primary Care Physician	Veterans meet with their Primary Care Provider and are referred to begin the AIM-Back program.	Primary Care Physician
**Weeks 0-6**	**Initial PT Session (Baseline):** The Veteran receives a comprehensive PT examination, evaluation, and treatment, including pain modulation techniques and/or pain neuroscience education. A personalized home exercise program is developed.<br><br>**First 6 Weeks Activities:** Weekly telehealth-based physical activity coaching is provided by a remote care provider to support adherence to the exercise program, including strength, flexibility, and activity goals.	On Site Physical Therapist<br><br>Telehealth Physical Therapist	**Initial Navigator Session (Baseline):** Veteran meets virtually with the Pain Navigator, who reviews their back pain history, prior treatments, care preferences, and options. Using shared decision-making, they select a non-pharmacologic treatment (e.g., PT, chiropractic, acupuncture).<br><br>Arrangements are made for the Veteran's preferred treatment, and they begin care at the first available appointment.	Pain Navigator
**Weeks 6-12**	**Follow-up PT Session:** After six weeks of remote care, the Veteran receives a follow-up PT session for re-evaluation and continued treatment, including pain modulation and/or pain neuroscience education.<br><br>**Activities:** Medium/high-risk Veterans (identified via the STarT Back Screening Tool) may receive up to six additional telehealth psychologically informed practice (PIP) sessions focused on pain coping strategies, education, goal setting, and relaxation techniques.	On Site Physical Therapist<br><br>Telehealth Physical Therapist	**Follow-up Navigator Session**: Veteran meets again virtually with the Navigator to review progress. Using shared decision-making, they may select additional or continued non-pharmacological treatment(s).<br><br>Arrangements are made for the new or continued services.	Pain Navigator
**Week 12**	**12-Week/3-Month Follow-up:** Veterans may complete a virtual visit with their remote provider to collect standardized patient-reported outcomes for the EHR. Based on preferences, they may be discharged or referred back to their PCP.	Telehealth Physical Therapist	**12-Week/3-Month Follow-up**: Veterans may complete a virtual visit with the Navigator to collect standardized patient-reported outcomes for the EHR. Based on preferences, they may be discharged or referred back to their PCP.	Pain Navigator

© 2026 George SZ et al. *JAMA Network Open.*

eTable 2. Patient-Reported Outcome Measures and Measurement Properties (CONSORT-PRO)

Instrument	Domain	Items	Recall Period	Score Range (Direction)	Validity Evidence	Reliability Evidence	Responsiveness / MCID	Assessment Time Points	Key References
PROMIS Pain Interference SF	Pain impact	4	Past 7 days	T-score 41.6–75.6 (higher = worse)	Construct validity vs legacy pain measures; validated in musculoskeletal & VA populations	Internal consistency α ≈0.95	Responsive; MCID ~3–5 points	Baseline, 3-month (EHR); 3, 6, 12-month (survey)	Cella et al., 2010; Amtmann et al., 2016
PROMIS Physical Function SF	Physical function	4	Past 7 days	T-score 22.5–57.0 (higher = better)	Validated vs SF-36 PF and performance measures	α ≈0.90–0.95	Responsive; MCID ~3–5 points	Same as above	Cella et al., 2010; Hays et al., 2013
PROMIS Sleep Disturbance SF	Sleep	4	Past 7 days	T-score (higher = worse)	Established construct validity in chronic pain	α >0.90	Responsive	Baseline, 3-month; survey subset	Yu et al., 2012
NIH Pain Intensity	Pain severity	1	Past 7 days	0–10 (higher = worse)	Face and construct validity	Test–retest reliability established	MCID ~1–2 points	Baseline, 3-month	Dworkin et al., 2005
PEG	Pain intensity/interference	3	Past 7 days	0–10 (higher = worse)	Validated brief pain measure	α ≈0.73–0.89	MCID ~1–2 points	Survey	Krebs et al., 2009
PSEQ-2	Pain self-efficacy	2	Current	0–12 (higher = better)	Validated short form	α >0.80	Responsive	Survey	Nicholas et al., 2015
EQ-5D-5L	Quality of life	5	Current	Index score + VAS	Widely validated across conditions	High test–retest reliability	Established MCIDs	Survey	Herdman et al., 2011
PHQ-2	Depressed mood	2	Past 2 weeks	0–6	Criterion validity vs PHQ-9	α ≈0.80	Screening tool	Survey	Kroenke et al., 2003
AUDIT-C	Alcohol use	3	Past year	0–12	Validated in VA populations	α ≈0.85	Screening tool	Survey	Bradley et al., 2007

Abbreviations: MCID = minimal clinically important difference; SF = short form; VA = Veterans Affairs.

© 2026 George SZ et al. *JAMA Network Open.*

eTable 3. Patient Characteristics From Survey at Baseline

Characteristic*	***Overall N=799***	***PNP N=399***	***SCP N=400***
Age (years), mean (SD)	*52.8 (15.2)*	*53.0 (15.5)*	*52.7 (15.0)*
Age (categories), no. (%)			
<50	*343 (42.9)*	*170 (42.6)*	*173 (43.3)*
50-64	*248 (31.0)*	*118 (29.6)*	*130 (32.5)*
65-74	*145 (18.1)*	*80 (20.1)*	*65 (16.3)*
>75	*63 (7.9)*	*31 (7.8)*	*32 (8.0)*
Sex, no. (%)			
Female	*88 (11.0)*	*40 (10.0)*	*48 (12.0)*
Male	*711 (89.0)*	*359 (90.0)*	*352 (88.0)*
Gender, no. (%)			
Woman	*87 (10.9)*	*38 (9.5)*	*49 (12.3)*
Man	*706 (88.4)*	*357 (89.5)*	*349 (87.3)*
Other	*2 (0.3)*	*0 (0.0)*	*2 (0.5)*
DK/Ref/Missing	*4 (0.5)*	*4 (1.0)*	*0 (0.0)*
Race, no. (%)			
Black or African American	*198 (24.8)*	*64 (16.0)*	*134 (33.5)*
*Other/Multiracial^*	*85 (10.6)*	*56 (14.0)*	*29 (7.3)*
White	*516 (64.6)*	*279 (69.9)*	*237 (59.3)*
Hispanic Ethnicity, no. (%)	*64 (8.0)*	*38 (9.5)*	*26 (6.5)*
CDC High Impact Chronic Pain, no. (%)	*473 (59.2)*	*246 (61.7)*	*227 (56.8)*
Missing	*5 (0.6)*	*2 (0.5)*	*3 (0.8)*
NIH Chronicity, no. (%)	*727 (91.0)*	*364 (91.2)*	*363 (90.8)*
Missing	*2 (0.3)*	*0 (0.0)*	*2 (0.5)*
Opioid Use, no. (%)	*70 (8.8)*	*41 (10.3)*	*29 (7.3)*
Chronic Opioid Use, no. (%)	*25 (3.1)*	*12 (3.0)*	*13 (3.3)*
Benzodiazepine Use, no. (%)	*39 (4.9)*	*22 (5.5)*	*17 (4.3)*
Chronic Benzodiazepine Use, no. (%)	*18 (2.3)*	*7 (1.8)*	*11 (2.8)*
PTSD, no. (%)	*191 (23.9)*	*91 (22.8)*	*100 (25.0)*
1 yr CAN Score, mean (SD)	*46.0 (29.6)*	*48.2 (29.7)*	*43.7 (29.4)*
Area Deprivation Index, mean (SD)	*56.9 (23.2)*	*59.3 (23.8)*	*54.6 (22.4)*
Additional Characteristics Collected in the PRO			
Highest Level of Education, no. (%)			

© 2026 George SZ et al. *JAMA Network Open.*

eTable 3. Patient Characteristics From Survey at Baseline

Characteristic*	***Overall N=799***	***PNP N=399***	***SCP N=400***
Some high school	*15 (1.9)*	*10 (2.5)*	*5 (1.3)*
High school graduate or equivalent (GED)	*165 (20.7)*	*82 (20.6)*	*83 (20.8)*
Trade/technical/vocational school	*30 (3.8)*	*18 (4.5)*	*12 (3.0)*
Some college credit but no degree	*224 (28.0)*	*109 (27.3)*	*115 (28.8)*
Associate's degree (AA or AS)	*116 (14.5)*	*60 (15.0)*	*56 (14.0)*
Bachelor's degree (BA or BS)	*154 (19.3)*	*76 (19.0)*	*78 (19.5)*
Post graduate work or graduate degree	*94 (11.8)*	*44 (11.0)*	*50 (12.5)*
DK/Ref/Missing	*1 (0.1)*	*0 (0.0)*	*1 (0.3)*
Employment Status, no. (%)			
Employed for wages full time (35+ hours/week)	*296 (37.0)*	*155 (38.8)*	*141 (35.3)*
Employed for wages part time (less than 35 hours/week)	*48 (6.0)*	*22 (5.5)*	*26 (6.5)*
Retired	*267 (33.4)*	*131 (32.8)*	*136 (34.0)*
Out of work and looking for work	*30 (3.8)*	*13 (3.3)*	*17 (4.3)*
Out of work but not currently looking for work	*12 (1.5)*	*5 (1.3)*	*7 (1.8)*
A homemaker	*6 (0.8)*	*6 (1.5)*	*0 (0.0)*
A student	*18 (2.3)*	*6 (1.5)*	*12 (3.0)*
Military (Reservist)	*3 (0.4)*	*1 (0.3)*	*2 (0.5)*
Unable to work - please describe reason	*114 (14.3)*	*57 (14.3)*	*57 (14.3)*
DK/Ref/Missing	*5 (0.6)*	*3 (0.8)*	*2 (0.5)*
Relationship Status, no. (%)			
Married or living together as married	*503 (63.0)*	*246 (61.7)*	*257 (64.3)*
Divorced/Separated	*139 (17.4)*	*77 (19.3)*	*62 (15.5)*
Widowed	*27 (3.4)*	*19 (4.8)*	*8 (2.0)*
Single, never married	*127 (15.9)*	*56 (14.0)*	*71 (17.8)*
DK/Ref/Missing	*3 (0.4)*	*1 (0.3)*	*2 (0.5)*
Living Situation, no. (%)			
Have a steady place to live	*765 (95.7)*	*379 (95.0)*	*386 (96.5)*
Have place to live today, worried about losing it in the future	*30 (3.8)*	*18 (4.5)*	*12 (3.0)*
Do not have a steady place to live	*3 (0.4)*	*2 (0.5)*	*1 (0.3)*
DK/Ref/Missing	*1 (0.1)*	*0 (0.0)*	*1 (0.3)*
Living Situation Problems, no. (%)			
Pests (bugs, ants, or mice)	*45 (5.6)*	*23 (5.8)*	*22 (5.5)*

© 2026 George SZ et al. *JAMA Network Open.*

eTable 3. Patient Characteristics From Survey at Baseline

Characteristic*	***Overall N=799***	***PNP N=399***	***SCP N=400***
Mold	*15 (1.9)*	*8 (2.0)*	*7 (1.8)*
Lead paint or pipes	*4 (0.5)*	*4 (1.0)*	*0 (0.0)*
Lack of heat	*3 (0.4)*	*3 (0.8)*	*0 (0.0)*
Oven/stove not working	*3 (0.4)*	*1 (0.3)*	*2 (0.5)*
Smoke detectors missing/not working	*5 (0.6)*	*3 (0.8)*	*2 (0.5)*
Water leaks	*10 (1.3)*	*2 (0.5)*	*8 (2.0)*
Multiple problems	*40 (5.0)*	*24 (6.0)*	*16 (4.0)*
None of the above problems	*670 (83.9)*	*329 (82.5)*	*341 (85.3)*
DK/Ref/Missing	*4 (0.5)*	*2 (0.5)*	*2 (0.5)*
Smoking, no. (%)	*172 (21.5)*	*90 (22.6)*	*82 (20.5)*
DK/Refused/Missing	*4 (0.5)*	*3 (0.8)*	*1 (0.3)*
Use of Nonpharmacological and Self-Care Approaches			
Acupuncture, no. (%)	*57 (7.2)*	*38 (9.5)*	*19 (4.8)*
DK/Ref/Missing	*1 (0.1)*	*0 (0.0)*	*1 (0.3)*
Manipulation, no. (%)	*160 (20.1)*	*85 (21.3)*	*75 (18.8)*
DK/Ref/Missing	*1 (0.1)*	*0 (0.0)*	*1 (0.3)*
Massage, no. (%)	*185 (23.2)*	*88 (22.1)*	*97 (24.4)*
Yoga, no. (%)	*96 (12.0)*	*47 (11.8)*	*49 (12.3)*
Tai Chi/Qigong, no. (%)	*14 (1.8)*	*8 (2.0)*	*6 (1.5)*
Exercise, no. (%)	*621 (77.9)*	*303 (75.9)*	*318 (79.9)*
Relaxation Techniques, no. (%)	*318 (40.1)*	*161 (40.6)*	*157 (39.6)*
DK/Ref/Missing	*1 (0.1)*	*1 (0.3)*	*0 (0.0)*
Meditation/Mindfulness, no. (%)	*206 (25.8)*	*100 (25.1)*	*106 (26.6)*
Psychotherapy/Counseling, no. (%)	*229 (28.7)*	*121 (30.3)*	*108 (27.1)*
DK/Ref/Missing	*1 (0.1)*	*1 (0.3)*	*0 (0.0)*

Missing characteristics imputed by site: 1yr CAN Score and ADI (mean); race and ethnicity (mode)

^ Other races included American Indian or Alaska Native, Asian, Native Hawaiian or Pacific Islander

© 2026 George SZ et al. *JAMA Network Open.*

eTable 4. Baseline Characteristics of EHR Patients by 3M Outcome Missingness

Characteristics*	***Not Missing N=1100***	***Missing N=717***
Age (years), mean (SD)	*53.3 (15.4)*	*52.5 (16.1)*
Age (categories), no. (%)		
<50	*444 (40.4)*	*322 (44.9)*
50-64	*372 (33.8)*	*190 (26.5)*
65-74	*186 (16.9)*	*144 (20.1)*
75+	*98 (8.9)*	*61 (8.5)*
Sex, no. (%)		
Female	*140 (12.7)*	*80 (11.2)*
Male	*960 (87.3)*	*637 (88.8)*
Race, no. (%)		
Black or African American	*332 (30.2)*	*209 (29.1)*
*Other/Multiracial^*	*44 (4.0)*	*24 (3.3)*
White	*724 (65.8)*	*484 (67.5)*
Hispanic Ethnicity, no. (%)		
Non-Hispanic	*1044 (94.9)*	*678 (94.6)*
Hispanic	*56 (5.1)*	*39 (5.4)*
CDC High Impact Chronic Pain, no. (%)	*735 (66.8)*	*459 (64.0)*
NIH Chronicity, no. (%)	*1014 (92.2)*	*658 (91.8)*
Opioid Use, no. (%)	*110 (10.0)*	*69 (9.6)*
Chronic Opioid Use, no. (%)	*38 (3.5)*	*26 (3.6)*
Benzodiazepine Use, no. (%)	*54 (4.9)*	*35 (4.9)*
Chronic Benzodiazepine Use, no. (%)	*22 (2.0)*	*16 (2.2)*
1yr CAN Score, mean (SD)	*46.9 (30.1)*	*44.7 (30.1)*
PTSD, no. (%)	*273 (24.8)*	*152 (21.2)*
Area Deprivation Index, mean (SD)	*58.0 (23.2)*	*57.2 (22.4)*

Missing characteristics imputed by site: 1yr CAN Score (mean), missing n=51 (41 PNP, 10 SCP); race and ethnicity (mode), missing n=36 (27 PNP, 9 SCP)

^ Other races included American Indian or Alaska Native, Asian, Native Hawaiian or Pacific Islander

© 2026 George SZ et al. *JAMA Network Open.*

eFigure 2a. Enrolled Participants (n=1817) With All Time Points (EHR and Survey) for PROMIS-SF Pain Interference A). Observed outcomes by days from EHR baseline visit with penalized B-Spline curves (blue SCP; red PNP; B) Estimated means (blue text SCP; red text PNP) and mean difference (black text) at follow-up time points for PROMIS Pain Interference with 97.5% confidence intervals from hierarchical penalized spline mixed-effects model

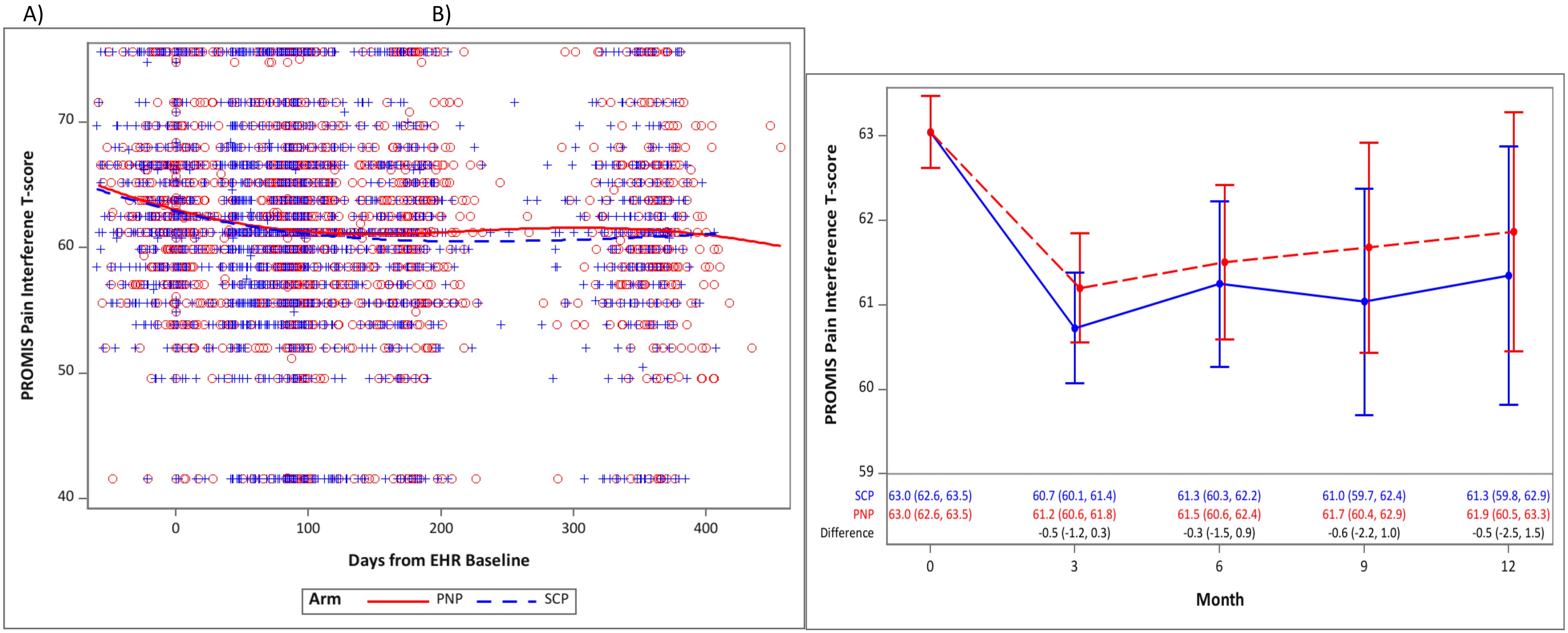

© 2026 George SZ et al. *JAMA Network Open.*

eFigure 2b. Enrolled Participants (n=1817) With All Time Points (EHR and Survey) for PROMIS-SF Physical Function A) Observed outcomes by days from EHR baseline visit with penalized B-Spline curves (blue SCP; red PNP; B) Estimated means (blue text SCP; red text PNP) and mean difference (black text) at follow-up time points for PROMIS Pain Interference with 97.5% confidence intervals from hierarchical penalized spline mixed-effects model

A) B)

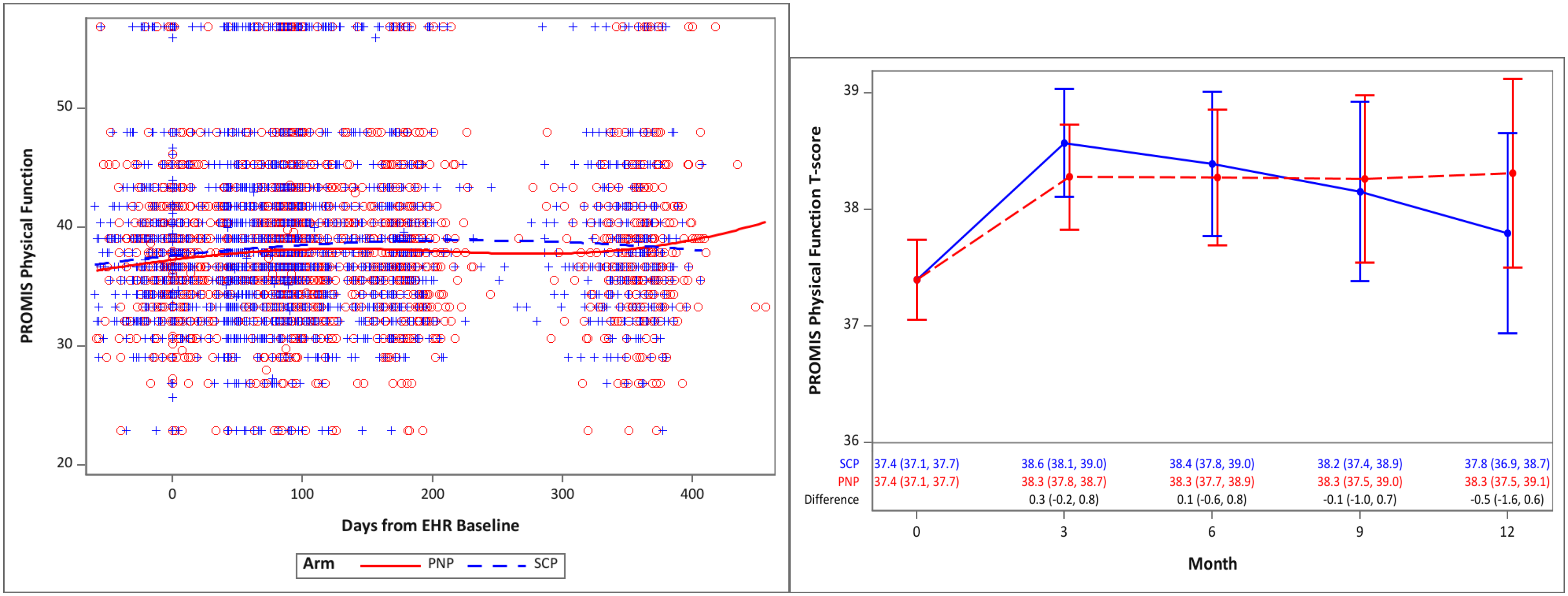

© 2026 George SZ et al. *JAMA Network Open.*

eFigure 3. Estimated Means (Blue Text SCP; Red Text PNP) and Mean Difference (Black Text) at Follow-Up Time Points for PROMIS Pain Interference and Physical Functions Outcomes and Associated 97.5% Confidence Intervals for Enrolled Participants (n=1817) From Multiply Imputed Data With Combined Estimates Across n=50 Imputation Fit to Hierarchical Linear Mixed Models

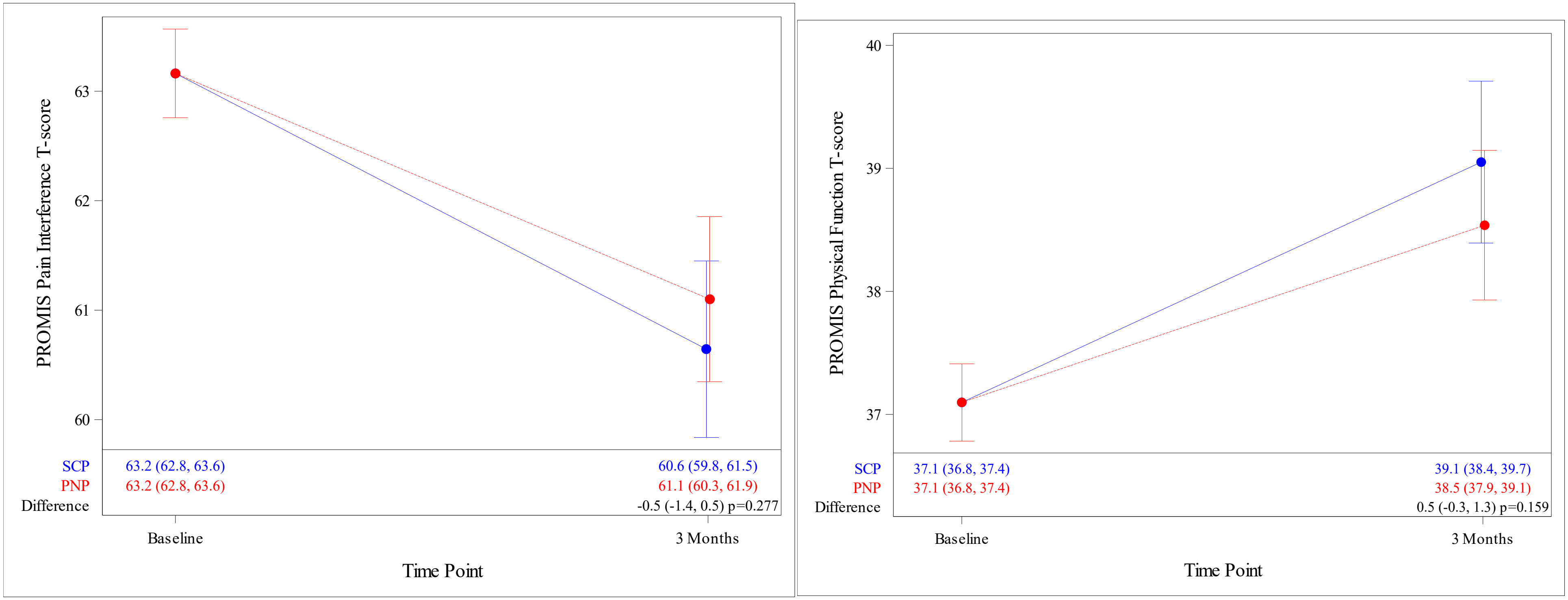

© 2026 George SZ et al. *JAMA Network Open.*

eFigure 4. Estimated Means (Blue Text SCP; Red Text PNP) and Mean Difference (Black Text) at Follow-Up Time Points for PROMIS Pain Interference and Physical Functions Outcomes and Associated 97.5% Confidence Intervals for Enrolled Participants (n=1817) From IPW Models Adjusted for Referral Bias Fit to Hierarchical Linear Mixed Models

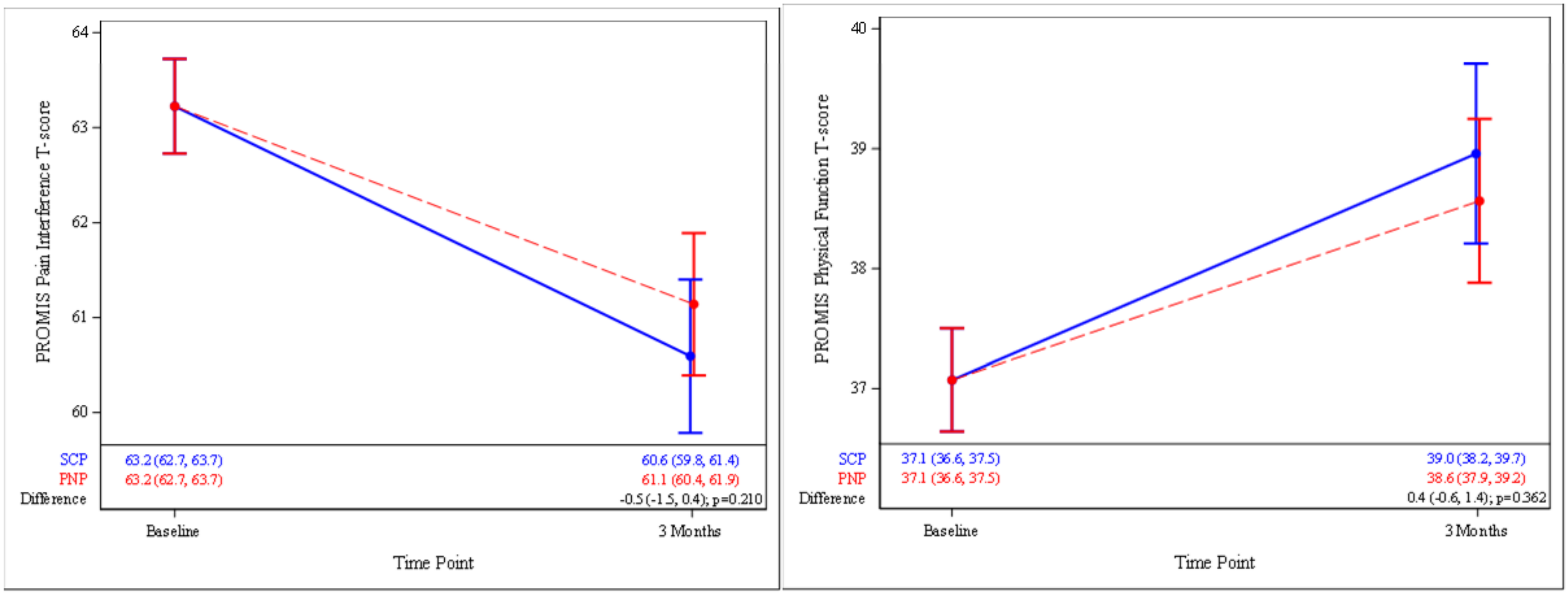

© 2026 George SZ et al. *JAMA Network Open.*

eFigure 5. Estimated Means (Blue Text SCP; Red Text PNP) and Mean Difference (Black Text) at Follow-Up Time Points for PROMIS Sleep Disturbance and NIH Pain Intensity Outcomes and Associated 95% Confidence Intervals for Enrolled Participants (Primary; n=1817) and Survey Participants (n=799) From Hierarchical Linear Mixed-Effects Models

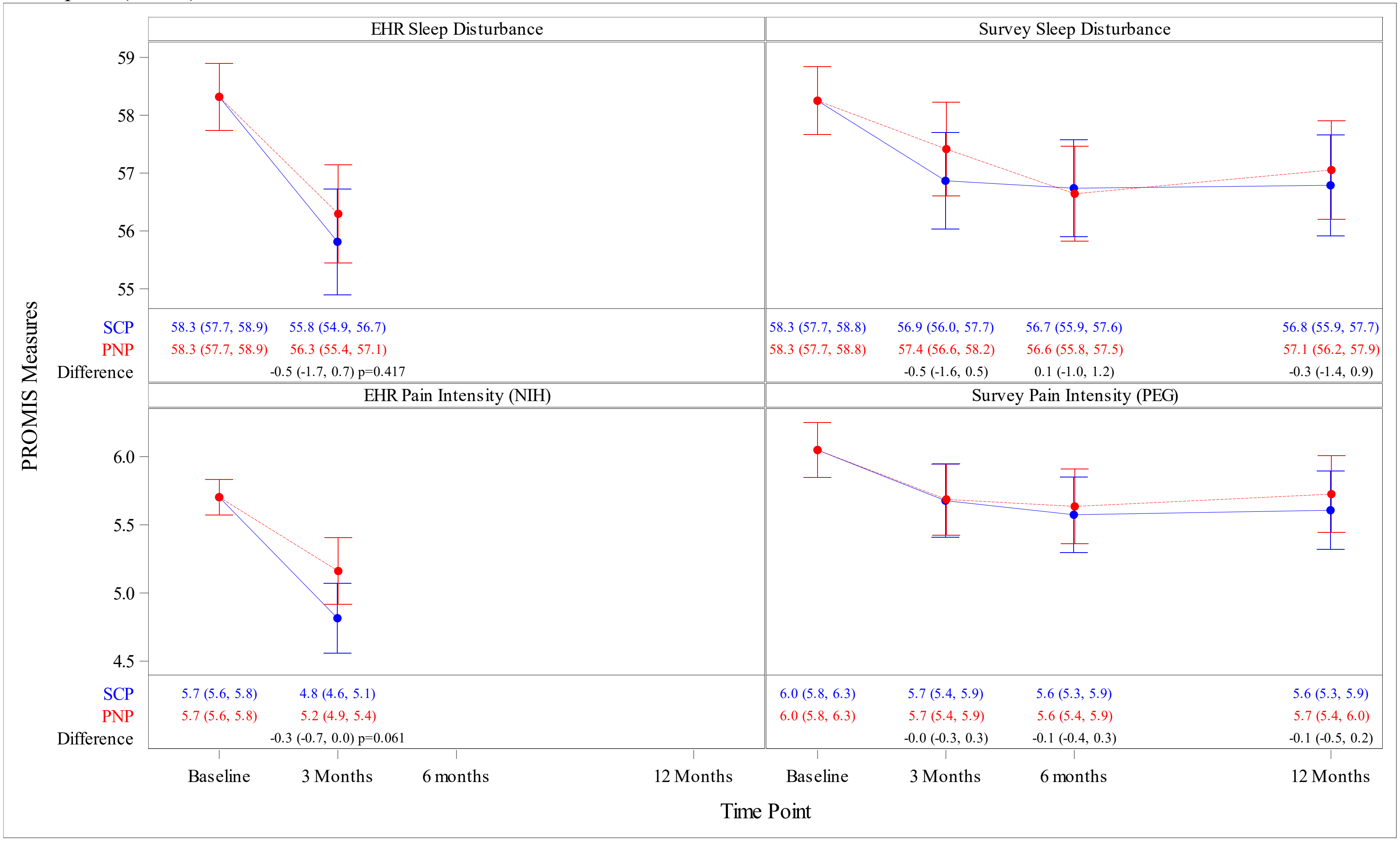

© 2026 George SZ et al. *JAMA Network Open.*

eTable 5. Estimated Means and Mean Differences at Follow-Up Time Points for Secondary Outcomes and Associated 95% Confidence Intervals for Survey Participants (n=799) From Hierarchical Linear Mixed-Effects Models

	Estimated Means			
**Outcome**	**Baseline**	**3-Month**	**6-Month**	**12-Month**
**Pain Intensity (PEG)**				
SCP	6.05(5.85, 6.25)	5.68(5.41,5.95)	5.57(5.30,5.85)	5.61(5.32,5.89)
PNP		5.69(5.42,5.95)	5.64(5.36,5.91)	5.73(5.44,6.01)
SCP-PNP Mean Difference	-	-0.01(-0.32,0.31)	-0.06(-0.39,0.27)	-0.12(-0.47,0.24)
**Pain Catastrophizing**				
SCP	0.83(0.77,0.90)	0.89(0.80,0.97)	0.91(0.82,0.99)	0.88(0.79,0.97)
PNP		0.93(0.84,1.01)	0.93(0.84,1.01)	0.94(0.85,1.03)
SCP-PNP Mean Difference	-	-0.04(-0.14,0.06)	-0.02(-0.13,0.09)	-0.06(-0.18,0.06)
**Self-Efficacy (PSEQ-2)**				
SCP	7.54(7.24,7.84)	7.55(7.16,7.94)	7.50(7.10,7.89)	7.40(7.00,7.80)
PNP		7.27(6.89,7.65)	7.31(6.92,7.70)	7.38(6.99,7.77)
SCP -PNP Mean Difference	-	0.28(-0.21,0.76)	0.19(-0.30,0.67)	0.02(-0.48,0.52)
**Quality of Life (EQ-5D-5L)**				
SCP	0.79(0.77,0.80)	0.76(0.74,0.79)	0.76(0.73,0.79)	0.76(0.74,0.79)
PNP		0.77(0.75,0.80)	0.76(0.73,0.79)	0.77(0.74,0.80)
SCP-PNP Mean Difference	-	-0.01(-0.04,0.03)	0.00(-0.04,0.04)	-0.01(-0.05,0.03)
**Depressed Mood (PHQ-2)**				
SCP	2.39(2.27,2.51)	2.45(2.29,2.62)	2.50(2.33,2.67)	2.35(2.18,2.52)
PNP		2.40(2.24,2.57)	2.34(2.17,2.50)	2.41(2.23,2.58)
SCP -PNP Mean Difference	-	0.05(0.17,0.26)	0.16(0.05,0.38)	-0.06(0.29,0.17)
**Alcohol Use (AUDIT-C)**				
SCP	1.97(1.75,2.19)	1.78(1.56,2.01)	1.81(1.58,2.04)	1.73(1.50,1.97)
PNP		1.86(1.64,2.09)	1.84(1.61,2.07)	1.81(1.58,2.05)
SCP-PNP Mean Difference	-	-0.08(-0.25,0.09)	-0.03(-0.21,0.16)	-0.08(-0.29,0.13)

© 2026 George SZ et al. *JAMA Network Open.*

eReferences.

1. Maroko AR, Doan TM, Arno PS, Hubel M, Yi S, Viola D. Integrating Social Determinants of Health With Treatment and Prevention: A New Tool to Assess Local Area Deprivation. *Prev Chronic Dis* 2016; **13**: 160221.

2. US Department of Veterans Affairs. Care Assessment needs (CAN) Primer). 2019.

3. Fitzmaurice GM, Laird NM, Ware JH. Applied Longitudinal Analysis [Internet]. 1st ed. Wiley; 2011 [cited 2025 Feb 26]. Available from: https://onlinelibrary.wiley.com/doi/book/10.1002/9781119513469

4. Buuren SV, Groothuis-Oudshoorn K. mice : Multivariate Imputation by Chained Equations in R. J Stat Soft [Internet] 2011 [cited 2025 Mar 10];45(3). Available from: http://www.jstatsoft.org/v45/i03/

5. Wijesuriya R, Moreno-Betancur M, Carlin JB, White IR, Quartagno M, Lee KJ. Multiple Imputation for Longitudinal Data: A Tutorial. Statistics in Medicine 2025;44(3–4):e10274.

6. Peskoe SB, Arterburn D, Coleman KJ, Herrinton LJ, Daniels MJ, Haneuse S. Adjusting for selection bias due to missing data in electronic health records-based research. Stat Methods Med Res 2021;30(10):2221–38.

7. Austin PC, Stuart EA. Moving towards best practice when using inverse probability of treatment weighting (IPTW) using the propensity score to estimate causal treatment effects in observational studies. Stat Med. 2015; 34:3661–3679.

© 2026 George SZ et al. *JAMA Network Open.*
