# Supplementary material for: Sequenced Care Pathway vs Pain Navigator Pathway for Veterans With Low Back Pain: The AIM-Back Cluster Randomized Clinical Trial
Source: JAMA Netw Open. 2026 Apr 2;9(4):e264421. doi: 10.1001/jamanetworkopen.2026.4421 (PMC13047465; doi:10.1001/jamanetworkopen.2026.4421)
Supplement: Supplement 3. — Data Sharing Statement [file jamanetwopen-e264421-s003.pdf]

Data Sharing Statement

George. Sequenced Care Pathway vs Pain Navigator Pathway for Veterans With Low Back Pain. *JAMA Netw Open*. Published April 02, 2026. doi:10.1001/jamanetworkopen.2026.4421

Data
Additional Information: clinicaltrials.gov (NCT04411420)
Data available: Yes
Data types: Deidentified participant data
How to access data: Deidentified data will be made available upon reasonable request and as allowable by current VA policies. Please make inquiries to vhaduraimback@va.gov.
When available: beginning date: 03-01-2026, end date: 03-01-2027

Supporting Documents
Document types: None

Additional Information
Who can access the data: Anyone requesting data
Types of analyses: for a specified purpose
Mechanisms of data availability: signed data access agreement
Any additional restrictions: Any of these activities will be made available following current VA policy on data sharing
